# A systematic review of the toxic potential of parabens in fish

**DOI:** 10.3389/ftox.2024.1399467

**Published:** 2024-10-07

**Authors:** Asok K. Dasmahapatra, Joydeep Chatterjee, Paul B. Tchounwou

**Affiliations:** ^1^ Department of BioMolecular Science, Environmental Toxicology Division, University of Mississippi, Oxford, MS, United States; ^2^ Department of Biology, University of Texas-Arlington, Arlington, TX, United States; ^3^ RCMI Center for Urban Health Disparities Research and Innovation, School of Computer, Mathematical and Natural Sciences, Morgan State University, Baltimore, MD, United States

**Keywords:** parabens, fish, oxidative stress, endocrine disruptors, behavior, neuromodulator

## Abstract

Parabens are the most prevalent ingredients in cosmetics and personal care products (PCPs). They are colorless and tasteless and exhibit good stability when combined with other components. Because of these unique physicochemical properties, they are extensively used as antimicrobial and antifungal agents. Their release into the aquatic ecosystem poses potential threats to aquatic organisms, including fish. We conducted an electronic search in PubMed (http://www.ncbi.nlm.nih.gov/pubmed) using the search term parabens and fish and sorted 93 articles consisting of methyl paraben (MTP), ethyl paraben (ETP), propyl paraben (PPP), butyl paraben (BTP), and benzyl paraben (BNP) in several fish species. Furthermore, we confined our search to six fish species (common carp, *Cyprinus carpio*; fathead minnows, *Pimephales promelas*; Japanese medaka, *Oryzias latipes*; rainbow trout, *Oncorhynchus mykiss*; Nile tilapia, *Oreochromis niloticus*; and zebrafish, *Danio rerio)* and four common parabens (MTP, ETP, PPP, and BTP) and sorted 48 articles for review. Our search indicates that among all six fish, zebrafish was the most studied fish and the MTP was the most tested paraben in fish. Moreover, depending on the alkyl chain length and linearity, long-chained parabens were more toxic than the parabens with short chains. Parabens can be considered endocrine disruptors (EDs), targeting estrogen-androgen-thyroid-steroidogenesis (EATS) pathways, blocking the development and growth of gametes, and causing intergenerational toxicity to impact the viability of offspring/larvae. Paraben exposure can also induce behavioral changes and nervous system disorders in fish. Although the USEPA and EU limit the use of parabens in cosmetics and pharmaceuticals, their prolonged persistence in the environment may pose an additional health risk to humans.

## 1 Introduction

**FIGURE 1:**
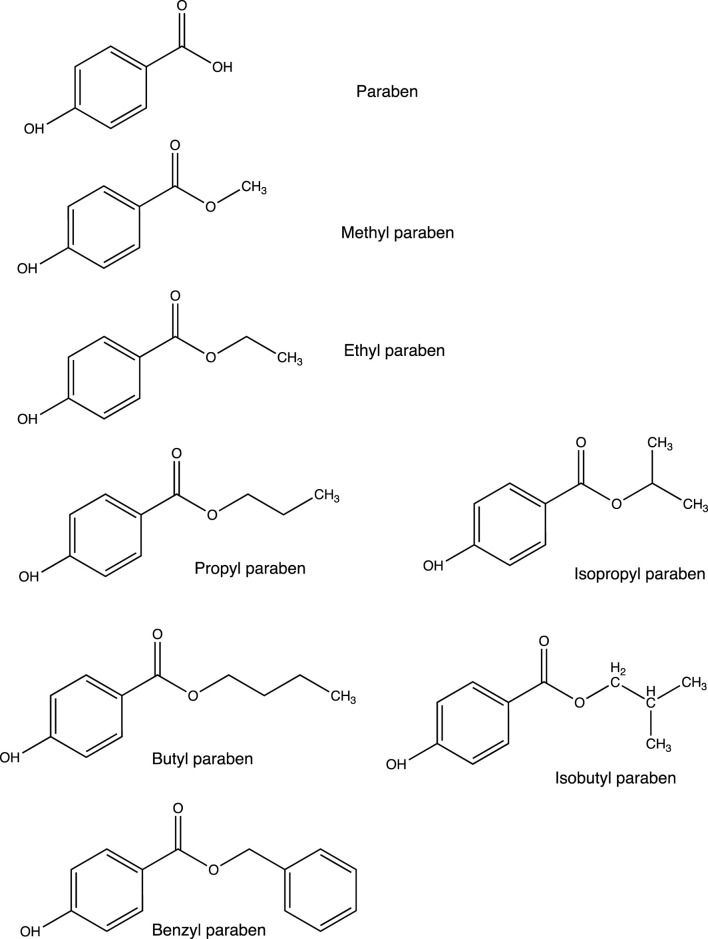
Chemical structures of the parabens used in our daily life.

Parabens consist of a group of artificial chemicals introduced in the mid-1920s (Liebert, 1984). Because of their antibacterial activities and high chemical stability, they are added to foodstuffs, pharmaceuticals, and personal care products as preservatives (Bledzka et al., 2014; Djatmika et al., 2016). Methyl paraben (MTP), ethyl paraben (ETP), propyl paraben (PPP), and butyl paraben (BTP) are the most commonly used parabens in commercial products (Junaid et al., 2019), with the maximum usage level of 0.4% for a single compound and 0.8% for mixtures (Nowak et al., 2021). In terms of chemical structures (Figure 1), they are esters of *p*-hydroxybenzoic acid with aryloxy or alkoxyl moieties of different lengths and side chains (Darbre et al., 2004; Hu et al., 2023b). With the increase in the length of the alkyl chain, the value of the octanol water-partition coefficient (usually expressed as log K_ow_) increases, resulting in decreased water solubility and increased lipophilicity (Golden et al., 2005; Artacho-Cordon et al., 2018).

Human exposure to parabens and the associated health risks have increased many-fold due to the increasing use of paraben-containing products (Ning et al., 2023). In the United States, ∼0.1% of parabens are used in bakery products, salad dressings, and pickles (Jay, 1995; Vandenberg et al., 2007). In Europe, paraben mixtures at concentrations of 0.4%–0.8% have been approved for use as a preservative in toiletries and cosmetic products (Andersen, 2008; Hill, 1995; Guru et al., 2021). The contents of parabens in processed products, such as fat, oil, seasonings, pickles, sauces, and soft drinks, ranged from 450 to 2000 mg/kg (Bolujoko et al., 2021; Ning et al., 2023). The daily intake of total parabens from cosmetics was estimated at 142.08 mg and 3.04 mg for adults and infants, respectively (Bledzka et al., 2014). The long-term intake of large amounts of parabens may be harmful to the human body. Because of their volatile or semi-volatile characteristics, six parabens were found in the indoor dust collected from China, with a total concentration ranging from 8.66 to 21,500 ng/g dw (Zhu et al., 2020). The parabens are also permeable to the blood–brain barrier, and MTP, ETP, and PPP have been detected in both the hypothalamus and white matter tissue in the human brain (van der Meer et al., 2017).

Parabens are also classified as endocrine-disrupting chemicals (EDCs) due to their ability to activate several nuclear receptors, causing changes in hormone-dependent signaling pathways (Schiller et al., 2013; Liang et al., 2023b; Dasmahapatra et al., 2023). Maternal exposure to parabens was reported to elevate the serum testosterone (T) concentration, impair the testicular structure, and affect sperm quality in male rats (Guerra et al., 2017). Studies indicated that parabens activate glucocorticoid receptor or peroxisome proliferator-activated receptor γ (PPAR-γ) in 3T3-L1 preadipocytes, showing the capacity to promote the adipogenic differentiation of the cells (Hu et al., 2013). Paraben exposure promoted the proliferation of MCF-7 cells, increased the luciferase activity in MVLN cells, and induced the vitellogenin (VTG) expression in zebrafish larvae, showing the typical estrogenic effects (Liang et al., 2023a).

The widespread application of parabens has caused considerable exposure risks to the environment and human beings (Zhu et al., 2024). Parabens can enter the environment through different sources and pathways (Cacua-Ortiz et al., 2020). Although wastewater treatment plants (WWTPs) can efficiently reduce the load of certain parabens, they cannot ensure the complete removal of all classes of these compounds (Golovko et al., 2021; Vale et al., 2022). Therefore, WWTP effluents are important sources of parabens in the aquatic environment, containing significant concentrations of these compounds and their metabolites (Li et al., 2015; Juksu et al., 2019). Inappropriate disposal and dumping of parabens have also resulted in the contamination of water bodies, affecting the aquatic environment and the health of the aquatic organisms (Xue et al., 2015; Berger et al., 2020; Leppert et al., 2020; Martins et al., 2023). As emerging pollutants, the incidence and behavior of parabens in the aquatic environment have yet to be fully studied. The aim of the present study is to review the effects of widely used parabens (MTP, ETP, PPP, and BTP) on fish using a wide range of endpoints in toxicology with a special focus on their developmental, reproductive, and neuro-behavioral effects.

## 2 Materials and methods

### 2.1 Literature search strategy

**FIGURE 2:**
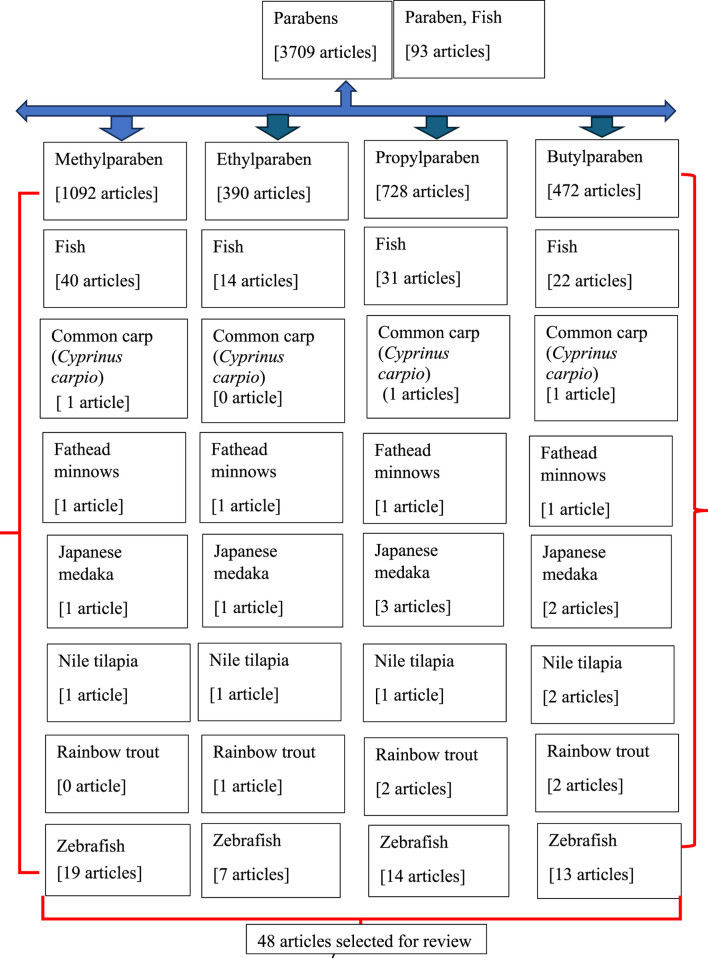
Flowchart of the articles searched in PubMed.

We performed a literature search to identify journal articles that reported the effects of parabens on fish, with a focus on development, reproduction, and neurobehavior. The electronic search was performed in PubMed (http://www.ncbi.nlm.nih.gov/pubmed) until 31 December 2023, using the search terms paraben, fish, the four common parabens (MTP, ETP, PPP, and BTP), and the common names of the six fish: common carp (*Cyprinus carpio*), fathead minnows *(Pimephales promelas*), Japanese medaka (*Oryzias latipes*), Nile tilapia (*Oreochromis niloticus*), rainbow trout (*Oncorhynchus mykiss*), and zebrafish (*Danio rerio*). PubMed was selected to identify journal articles as it is considered a main and reliable source of scientific information. Moreover, in this review, we have restricted our search mostly to bony fish, and these six fish species can represent well the class Osteichthyes. After a literature search, we found 48 peer-reviewed articles and 1 abstract (Mikula et al., 2006b) showing potential toxic, reproductive, neurological, and behavioral disorders induced by parabens on fish (Figure 2; Tables 1, 2). We have assembled all the information in Supplementary Table S1 and deposited it in a repository [Figshare (https://figshare.com/account/items/24565750/edit)] for reference.

**TABLE 1:** List of common fish exposed to common parabens (MTP, ETP, PPP, and BTP).

Fish	Paraben	Reference	Number of articles
Common carp	MTP	Medkova et al. (2023)	1
	ETP		0
	PPP	Medkova et al. (2023)	1
	BTP	Medkova et al. (2023)	1
Fathead minnows	MTP	Dobbins et al. (2009)	1
	ETP	Dobbins et al. (2009)	1
	PPP	Dobbins et al. (2009)	1
	BTP	Dobbins et al. (2009)	1
Japanese medaka	MTP	Yamamoto et al. (2011)	1
	ETP	Yamamoto et al. (2011)	1
	PPP	Inui et al. (2003); Yamamoto et al. (2011); Gonzalez-Doncel et al. (2014)	3
	BTP	Yamamoto et al. (2007; 2011)	2
Nile tilapia	MTP	Silva et al. (2018)	1
	ETP	Silva et al. (2018)	1
	PPP	Silva et al. (2018)	1
	BTP	Silva et al. (2018); Liu et al. (2023)	2
Rainbow trout	MTP	Grung et al. (2007) ^a^	0 + 1
	ETP	Pedersen et al. (2000)	1
	PPP	Pedersen et al. (2000); Bjerregaard et al. (2003)	2
	BTP	Pedersen et al. (2000); Alslev et al. (2005)	2
Zebrafish	MTP	Dambal et al. (2017); Hassanzadeh, (2017); Ates et al. (2018); Bereketoglu and Pradhan, (2019); Raja et al. (2019); Merola et al. (2020b); de Carvalho Penha et al. (2021); Merola et al. (2021); Hu et al. (2022a, b; 2023a; 2023b); Liang et al. (2022; 2023a; 2023b); Medkova et al. (2023); Shi et al. (2023); Thakkar et al. (2022); Tran et al. (2023)	19
	ETP	Merola et al. (2020a; 2021); Fan et al. (2022); Liang et al. (2022; 2023a; 2023b); Tran et al. (2023)	7
	PPP	Mikula et al. (2006a, 2006b^a^; 2009); Torres et al. (2016); Pisera-Fusyer et al. (2017); Bereketoglu and Bereketoglu and Pradhan, 2019; Perugini et al. (2020); Golovko et al. (2021)^a^; Liang et al. (2022; 2023a; 2023b); Lite et al. (2022); Medkova et al. (2023); Merola et al. (2022; 2024); Tran et al. (2023)	14 + 2^a^
	BTP	Brown et al. (2018); Merola et al. (2020a; 2021); Kim et al. (2022); Liang et al. (2022; 2023a; 2023b); Lite et al. (2022); Li et al. (2023); Liu et al. (2023) Medkova et al. (2023); Zhu et al. (2023); Singh et al. (2024)	13

Dobbins et al. (2009) also investigated the effects of i-PPP, i-BTP, and BNP on fathead minnow.

^a^ Golovko et al. (2021) studied the effects of PPP as a component in water from wastewater treatment plants using zebrafish embryos.

*Grung et al. (2007) studied the effects of a main sewer consisting of MTP in the primary culture of rainbow trout hepatocytes.

*Mikula et al. (2006b) published an abstract in Toxicology Letters 164s (2006), s1-s324, P11-12 (did not include during counting).

Yamamoto et al. (2007) also investigated the effects of BNP on Japanese medaka.

Yamamoto et al. (2011) also studied the effects of i-PPP, i-BTP, and BNP on Japanese medaka.

Silva et al. (2018) also studied the effects of BNP on Nile tilapia.

MTP, methyl paraben; ETP, ethyl paraben; i-PPP, isopropyl paraben; PPP, propyl paraben; i-BTP, isobutyl paraben; BTP, butyl paraben; BNP, benzyl paraben.

**TABLE 2:** List of authors who investigated the effects of parabens on the six selected fish.

Reference	Fish	MTP	ETP	PPP	BTP
Alslev et al. (2005)	Rainbow trout				
Ates et al. (2018)	Zebrafish				
Bereketoglu and Pradhan (2019)	Zebrafish				
Bjerregaard et al. (2003)	Rainbow trout				
Brown et al. (2018)	Zebrafish				
Dambal et al. (2017)	Zebrafish				
De Carvalho Penha et al. (2021)	Zebrafish				
Dobbins et al. (2009)	Fathead minnows				
Fan et al. (2022)	Zebrafish				
Golovko et al. (2021)	Zebrafish				
Gonzalez-Doncel et al. (2014)	Japanese medaka				
Hassanzadeh, (2017)	Zebrafish				
Hu et al. (2022a)	Zebrafish				
Hu et al. (2022b)	Zebrafish				
Hu et al. (2023b)	Zebrafish				
Hu et al. (2023a)	Zebrafish				
Inui et al. (2003)	Japanese medaka				
Kim et al. (2022)	Zebrafish				
Li et al. (2023)	Zebrafish				
Liang et al. (2022)	Zebrafish				
Liang et al. (2023a)	Zebrafish				
Liang et al. (2023b)	Zebrafish				
Lite et al. (2022)	Zebrafish				
Liu et al. (2023)	Nile tilapia				
Medkova et al. (2023)	Common carp				
Medkova et al. (2023)	Zebrafish				
Merola et al. (2020a)	Zebrafish				
Merola et al. (2020b)	Zebrafish				
Merola et al. (2021)	Zebrafish				
Merola et al. (2022)	Zebrafish				
Merola et al. (2024)	Zebrafish				
Mikula et al. (2006a, 2006b)	Zebrafish				
Mikula et al. (2009)	Zebrafish				
Pedersen et al. (2000)	Rainbow trout				
Perugini et al. (2020)	Zebrafish				
Pisera-Fuster et al. (2017)	Zebrafish				
Raja et al. (2019)	Zebrafish				
Shi et al. (2023)	Zebrafish				
Silva et al. (2018)	Nile tilapia				
Singh et al. (2024)	Zebrafish				
Thakkar et al. (2022)	Zebrafish				
Torres et al. (2016)	Zebrafish				
Tran et al. (2023)	Zebrafish				
Yamamoto et al. (2007)	Japanese medaka				
Yamamoto et al. (2011)	Japanese medaka				
Zhu et al. (2023)	Zebrafish				

## 3 Results

### 3.1 MTP

MTP is an ester of *p*-hydroxybenzoic acid (Figure 1) used as an antimicrobial preservative in cosmetics, drugs, and food (Dambal et al., 2017). In the United States, it is the most abundant paraben found in indoor dust, with a median concentration of 1,920 ng/L (Chen et al., 2018). In the urine of a pregnant woman from Greece, the concentration of MTP was found to be 67,461 μg/L (Myridakis et al., 2015). Because of its good aqueous solubility, it is the most detected paraben in the environment (Costa et al., 2017; Czarczynska-Goslinska et al., 2017; Hu et al., 2023). Moreover, MTP was found at concentrations of 242 ng/L in mineral and treated water (Marta-Sanchez et al., 2018). The concentration of MTP in the surface water in Osun State, Nigeria, was 163 μg/L, and in groundwater, it was 68 μg/L (Bolujoko et al., 2022). MTP (1,062 ng/L) was detected in the Xiangjiang River of China (Lu et al., 2018) and in the effluent of the main sewer in Zagreb, Croatia (Grung et al., 2007). A significant amount of MTP was detected in the body of the stripped catfish (*Pseudoplatystoma magdaleniatum*) found in the Cauca and Magdalena rivers of Colombia (Cacua-Ortiz et al., 2020). MTP, found in the sewage treatment work (STW) effluent of the city of Zagreb, Croatia, was shown to have estrogenic effects (VTG induction) in rainbow trout hepatocytes *in vitro* (Grung et al., 2007). To date, the most severe pollution level of MTP was recorded in Nigeria, where MTP concentrations reached 527 and 212 μg/L in surface and groundwater, respectively (Bolujoko et al., 2022).

#### 3.1.1 Common carp

In common carp, the toxic limit of MTP was evaluated only in embryos (embryo–larval toxicity tests) although the LC_50_ remained undermined (Tables 3, 4; Supplementary Table S1) (Medkova et al., 2023). All embryos died (100%) at a concentration of 1 mg/L of MTP. Moreover, delayed hatching was also observed in a concentration-dependent manner. Other endpoints (reproductive, neurological, and behavioral) using larvae and adults of the common carp are yet to be investigated.

**TABLE 3:** Lethal concentrations of parabens in fish.

Paraben	Fish (stage)	LC_50_ (mg/L)	LC_50_ (µM)	Benchmark dose (lower bound BMDL–upper bound BMDU)	NOEC	LOEC	Reference
MTP	Fathead minnows (1 dpf)	>160 mg/L (48 h)	>1,052 µM (48 h)			25 mg/L	Dobbins et al. (2009)
	Japanese medaka (10 days old)	63 mg/L (96 h)	414.06 µM (96 h)	(50–93 mg/L)	160 μg/L		Yamamoto et al. (2011)
	Nile tilapia (adult male)	67.11 mg/L (48 h)	441.08 µM (48 h)	5.38–11.83 mg/L		4.0 mg/L	Silva et al. (2018)
	Zebrafish (embryos)	50 mg/L (48 h)	328.62 µM (48 h)				Ates et al. (2018)
	Zebrafish (embryos)	65.1 mg/L (96 h)	428 µM (96 h)				Dambal et al. (2017)
	Zebrafish (embryos)	72.67 mg/L (96 h)	477.62 µM (96 h)	40.8–57.4 mg/L			Merola et al. (2020b)
	Zebrafish (embryos)	70.26 mg/L (120 h)	468.14 µM (120 h)	309.51–636.78 µM (47.1 mg/L–97 mg/L)			Tran et al. (2023)
	Zebrafish (larvae)	211.12 mg/L (168 h)	1,387.5 µM (168 h)		60 mg/L (394.34 µM) (168 h)		De Carvalho Penha et al. (2021)
	Zebrafish (adult male fish)	105.09 mg/L (96 h)	691.35 µM (96 h)		50 mg/L (128.62 µM) (96 h)		De Carvalho Penha et al. (2021)
	Zebrafish (adults)	1.102 mg/L (96 h)	7.24 µM (96 h)				Thakkar et al. (2022)
ETP	Fathead minnows (1 dpf)	34.3 mg/L (48 h)	86.05 µM (48 h)			17 mg/L	Dobbins et al. (2009)
	Japanese medaka (10 days old)	14 mg/L (96 h)	84.26 µM (96 h)	10–19 mg/L			Yamamoto et al. (2011)
	Nile tilapia (adult male)	24.08 mg/L (48 h)	144.91 µM	18.70–31.02 mg/L		4.0 mg/L	Silva et al. (2018)
	Zebrafish (embryos)	20.86 mg/L (96 h)	125.53 µM (96 h)	10.8–17.4 mg/L			Merola et al. (2020a)
	Zebrafish (embryos)	28.70 mg/L (96 h)	172.71 µM (96 h)				Fan et al. (2022)
	Zebrafish (embryos)	32.57 mg/L (120 h)	196 µM (120 h)	136.27–257.384 µM			Tran et al. (2023)
PPP	Fathead minnows (1 dpf)	9.7 mg/L (48 h) (iso-PPP = 17.5 mg/L)	53.83 µM (48 h) (iso-PPP = 97.11 µM)			2.5 mg/L [iso-PPP = 9.0 mg/L]	Dobbins et al. (2009)
	Japanese medaka (10 days old)	4.9 mg/L (96 h) (i-PPP = 4.5 mg/L; 96 h)	27.19 µM (96 h) (i-PPP = 24.97 µM; 96 h)	3.6–6.7 mg/L			Yamamoto et al. (2011)
	Nile tilapia (adult male)	17.36 mg/L (48 h)	96.34 µM (48 h)	14.63–20.61 mg/L		4.0 mg/L	Silva et al. (2018)
	Zebrafish (embryos)	NA	NA	NA	1 mg/L (80 h)	3.5 mg/L (80 h)	Torres et al. (2016)
	Zebrafish (embryos)	3.98 mg/L (96 h)	22.08 µM (96 h)				Perugini et al. (2020)
	Zebrafish (embryos)	11.14 mg/L (120 h)	61.8 µM (120 h)	39.5–84.2 µM (120 h)			Tran et al. (2023)
BTP	Fathead minnows (1 dph)	4.2 mg/L (48 h) (i-BTP = 6.9 mg/L)	21.62 µM (48 h) (iso-BTP = 35.52 µM)			1.0 mg/L [i-BTP = 3.5 mg/L]	Dobbins et al. (2009)
	Japanese medaka (10 days old)	3.1 mg/L (96 h) (i-BTP = 4.6 mg/L)	15.96 µM (96 h) (i-BTP = 23.68 µM)		40 μg/L (i-BTP = 20 μg/L)		Yamamoto et al. (2011)
	Japanese medaka (larvae 10 days old)	2.9 mg/L (96 h) (i-BTP = 4.6 mg/L; 96 h)	14.93 µM (96 h) (i-BTP = 23.68 µM)	3.5–4.8 mg/L (96 h) (i-BTP = 3.9–5.2 mg/L; 96 h			Yamamoto et al. (2007)
	Nile tilapia (adult male)	7.80 mg/L (48 h)	40.16 µM (48 h)	6.16–9.89 mg/L		4.0 mg/L	Silva et al. (2018)
	Zebrafish (embryos)	10.77 mg/L (24 h)	55.45 µM (24 h)				Li et al. (2023)
	Zebrafish (embryos)	4.208 mg/L (48 h)	21.66 µM (48 h)				Li et al. (2023)
	Zebrafish (embryos)	1.953 mg/L (72 h)	10.056 µM (24 h)				Li et al. (2023)
	Zebrafish (embryos)	2.74 mg/L (72 h)	14.11 µM (72 h)				Zhu et al. (2023)
	Zebrafish (embryos)	1.359 mg/L (96 h)	7 µM (96 h)				Li et al. (2023)
	Zebrafish (embryos)	2.34 mg/L (96 h)	12.04 µM (96 h)	0.91–1.92 mg/L (96 h)			Merola et al. (2020a)
	Zebrafish (embryos)	0.966 mg/L (120 h)	4.97 µM (120 h)				Li et al. (2023)
BNP	Fathead minnows (1 dph)	3.3 mg/L (48 h)	14.46 µM (48 h)			1.7 mg/L	Dobbins et al. (2009)
	Japanese medaka (larvae 10 days old)	0.73 mg/L (96 h)	3.2 µM (96 h)	3–4.3 mg/L (96 h)	20 μg/L		Yamamoto et al. (2007, 2011)
	Nile tilapia (adult male)	7.98 mg/L (48 h)	34.97 µM (48 h)	5.38–11.83 mg/L		4.0 mg/L	Silva et al. (2018)

MTP, methyl paraben; ETP, ethyl paraben; PPP, propyl paraben; BTP, butyl paraben; dph = day post-hatch.

**TABLE 4:** Effects of MTP on fish.

Fish	Author	Toxicological endpoint	Reproductive/endocrine-related endpoint	Neurobehavioral endpoint
Common carp (embryo)	Medkova et al. (2023)	i) Concentration-dependent mortality (embryo) ii) Hatching delay		
Fathead minnows (1 dph larvae)	Dobbins et al. (2009)	i) 48 h LC_50_ was > 160 mg/L ii) LOEC for larval growth = 25 mg/L iii) Hazard quotient for larval growth = 9 × 10^−^ ^5^		
Japanese medaka (10 dph larvae)	Yamamoto et al. (2011)	i) 96 h LC_50_ in 10 dph larvae = 63 mg/L	i) Plasma VTG enhanced in male fish ii) Upregulation of 13 genes, including *vtg2*, *chgL*, *chgH*, and *esr1* in the liver of male fish iii) 10 genes were downregulated in the liver of male fish	
Nile tilapia (adult male fish)	Silva et al. (2018)	i) 48 h LC_50_ in adult male fish was 67.11 mg/L ii) Lipid peroxidation (GSH and MDA contents) in the gills and liver lacks consistency (gills, unresponsive; liver GSH decreased after 6 days and increased after 12 days of exposure; MDA unresponsive) iii) Oxidative stress-related enzymes (CAT, GPx, GR, and SOD) in the gills and liver showed alteration, which is specific to the duration of exposure (6 and 12 days) and tissues (liver and gills)		
Zebrafish (embro-larval)	Dambal et al. (2017); Ates et al. (2018); Bereketoglu and Pradhan, (2019); Raja et al. (2019); Merola et al. (2020b); Merola et al. (2020b); De Carvalho Penha et al. (2021); Liang et al. (2022; 2023a 2023b); Medkova et al. (2023); Shi et al. (2023); Tran et al. (2023)	i) Depending on the duration and developmental stage, LC_50_ in embryos, larvae, and adults showed a wide range of variability ii) In embryos, the yolk (yolk sac edema), heart (pericardial edema and heart rates), blood (accumulation and circulation), and hatching processes were affected iii) The larvae showed bent spine and reduced body length, and pigmentation defects observed iv) Reduction in the GST activity and NO levels; however, MDA activity increased v) Upregulation of *ccdn1*, *myca*, and *pmoc* genes vi) Downregulation of *mgst*, *gst*, *gadd45a*, *il8*, *ldlr*, *lpl*, *ar*, *ttr*, *gr*, *mr*, *crhr2*, *hspl*, *and hsp90* vii) The expression of several genes, including *cat*, *sod3*, *hsp70*, *mt1 bax*, and *bcl2* remained unaltered	i) Upregulation of *vtg1* mRNA; downregulation of *ar,* and no alteration in *esr1* ii) Decrease in ACTH and increase in cortisol levels iii) Decrease in T3, T4, and T levels	i) Reduced swimming distance and mean velocity (inhibition of locomotor activity) ii) Induced anxiety-like behavior iii) No significant effects on thigmotaxis, startle response, and photic entrainment of locomotor activity
Zebrafish (adult)	Hassanzadeh, 2017; de Carvalho Penha et al. (2021); Thakkar et al. (2022); Hu et al. (2022a, 2022 b, 2023a, 2023b)	i) Short exposure (96 h) and comparatively higher concentration of MTP (50 mg/L) decreased EDOD activity and augmented LPO in the gills ii) Micronuclei in RBC enhanced in a concentration-dependent manner iii) Sex-specific growth (length and weight), enhanced in female fish not in male fish iv) Induced hepatocellular vacuolization v) Lipid metabolism in the gut, blood, and liver were dysregulated vi) Hepatic concentration of cortisol in the male liver increased vii) Inhibits the synthesis and conjugation of primary bile acid in liver of female fish viii) Degradation of retinoic acid and estradiol was enhanced in the liver ix) Concentration-dependent disruption of the gut microbiota x) Sex-specific effects in goblet cell density, expression of tight junction protein, and serotonin concentration in intestine (increased in male fish and decreased in female fish) xi) Parental exposure induced developmental deficits in offspring (increased mortality, precocious hatching, and elevated heart rates) xii) Downregulation of *hif-1α* (cell proliferation and apoptosis), *tnnt2*, *pax-6b*, *and ntrk2a* genes	i) Increased GSI; significant decrease in the blood cortisol level ii) Blocked both spermatogenesis and oogenesis iii) Imbalance in E2, T, and 11-KT in the circulating blood iv) In the male liver, downregulation of *esr2a* and *vtg1* mRNAs v) Upregulation of *hmgrb*, *cyp11a*, and *cyp17* in the testis vi) Downregulation of *star, 3βhsd*, *17βhsd*, and *cyp19a* in the testis vii) In the brain of female fish, upregulation of *esr2b* and downregulation of *gnrh3* occurred viii) In the ovary, downregulation of *star* and *cyp17* genes occurred ix) In the female liver, downregulation of *vtg1* mRNA occurred	i) Cranio-somatic index (CSI) was increased in female fish ii) AChE activity decreased in both sexes iii) In the male brain, a concentration-dependent increase was observed in ROS, followed by a decrease in GSH content and CAT activities iv) Sex-specific effect on the glutamate content in the brain (increase in male fish and decrease in female fish) v) Downregulation of *crh* and *crhbp* genes in both sexes vi) Sex-specific effects were observed in neurobehavior (distance traveled, swimming speed, and freezing time)

#### 3.1.2 Fathead minnows

In fathead minnows, 1-day post-hatch (dph) larvae were used for toxicological assessments (Tables 3, 4; Supplementary Table S1). The lowest observed effect concentration (LOEC) for larval growth was 25 mg/L (Dobbins et al., 2009); however, the 48 h LC_50_ is >160 mg/L (>1,052 µM). The hazard quotient for larval growth was 9 × 10^−5^. The effects of MTP on embryos and adults are yet to be investigated; other endpoints, like reproduction and neurobehavior, need to be studied in embryos, larvae, and adults.

#### 3.1.3 Japanese medaka

Both larvae (10 dph) and adult male fish (3 months old) were used for toxicological assessment (LC_50_) and reproductive endpoints (plasma VTG) (Tables 3 and 4, Supplementary Table S1). The 96 h LC_50_ value for larvae (10 dpf) was 63 mg/L (benchmark dose: 50–93 mg/L). The plasma VTG level in adult male fish (3 months old) enhanced after MTP exposure (14 days), indicating the estrogenic effect of MTP on medaka (Yamamoto et al., 2011). Gene expression analysis (10 μg/L) in the liver of male fish (14 days of exposure) showed upregulation of 13 genes including *vtg2*, *chgL*, *chgH*, and *esr1*, and downregulation of 10 genes (Yamamoto et al., 2011). The effects of MTP on embryo–larval development and neurobehavior of medaka are yet to be investigated.

#### 3.1.4 Nile tilapia

Only adult male fish were evaluated for the toxicological endpoints of MTP (Tables 3, 4; Supplementary Table S1) (Silva et al., 2018). The 48 h LC_50_ value was 67.11 mg/L (56.61–79.57 mg/L). Adult male fish were also used for the assessment of lipid peroxidation (LPO) (GSH and MDA content) and oxidative stress-related enzyme activities (CAT, GPx, GR, and SOD) in the gills and liver after exposure to MTP (4 mg/L) for 6 and 12 days. It was observed that GSH content in the gills remained unresponsive, while in the liver, it decreased after 6 days and increased after 12 days of exposure (Silva et al., 2018). MDA levels in both the liver and gills remained unresponsive to MTP (Supplementary Table S1). CAT activity in the gills remained unaltered, while it was enhanced only in fish exposed for 6 days (Table 4; Supplementary Table S1). GPx activity enhanced in the gills after 6 days (no alteration in the liver), while GR activity increased in the liver after 12 days of exposure (no effect on the gills). Studies on embryo-larval development, reproduction, and neurobehavioral assessment following MTP exposure are yet to be investigated.

#### 3.1.5 Zebrafish

The effects of MTP were investigated on embryos, larvae, and adult zebrafish (Tables 1–4; Supplementary Table S1). The concentration and duration of exposure of the embryos, larvae, and adults in MTP were widely variable. The calculated LC_50_ values for embryos (50–72.67 mg/L or 329–478 μM; duration of exposure: 48–120 hpf) (Dambal et al., 2017; Ates et al., 2018; Merola et al., 2020b; Tran et al., 2023); larvae (211.12 mg/L or 1,388 μM; duration of exposure: 7 days; de Carvalho Penha et al., 2021); and adults (1.102–105.09 mg/L or 7.24–691.35 µM; 96 h duration) (de Carvalho Penha et al., 2021; Thakkar et al., 2022) are not identical (Table 3), and in most cases, the embryos were found to be more sensitive than larvae and adults. In *ovo* observations related to the heart (pericardial edema and heart rate), yolk (yolk sac edema), and blood circulation (blood stasis, cardiac output, stroke volume, and blood clots) indicated toxic potentials of MTP that resulted in malformed larva. Moreover, hatching was delayed by MTP in a concentration-dependent manner, and the hatched larvae developed bent spines, axial malformations, and pigmentation defects. The toxic index (TOI) was 16–26.5 mg/L, and the teratogenic index (TEI) was 1.8. The effects on LPO (NO and MDA activity) and oxidative-stress enzyme (GST) indicated a significant alteration after MTP exposure (Table 4; Supplementary Table S1). Moreover, MTP showed potential endocrine-disrupting (ED) effects in larvae by reducing testosterone (T) and thyroid hormone (T3 and T4) contents/levels while increasing the cortisol level, followed by a decrease in the ACTH content. AChE activity was increased in embryos/larvae at 56 hours post-fertilization (hpf) (Shi et al., 2023) but decreased in larvae at 6 days post-fertilization (dpf) (Raja et al., 2019). MTP induced anxiety-like behavior inhibited locomotor activities in larvae; however, no effect was observed on photic entrainment of locomotor activities (Table 4). Several genes, including *vtg1* (estrogen-responsive), *ccnd1* (oncogene), *myca* (cell proliferation), and *pmoc* (HPI-axis), showed concentration-dependent upregulation, while *gadd45a* (growth arrest), *ldlr* (fatty acid metabolism), *ar* (HPG-axis), *ttr* (HPT-axis) *gr*, *mr*, and *crhr2* (HPI-axis), *hsp70l* (lens formation), and *hsp90* (differentiation of somatic muscle pioneer cells) were downregulated by MTP exposure (Table 4). The expression of *cat*, *sod3*, *hsp70*, and *mt1* (oxidative stress) and *bax* and *bcl2* (cell cycle and apoptosis) did not alter after MTP exposure (Table 4).

In adults, MTP exposure was slightly modified, either exposed to higher concentrations (30 µg/L-150 mg/L) with shorter durations (96 h) (de Carvalho Penha et al., 2021) or lower concentrations (0.001–10 μg/L) with longer durations (21–30 days) (Hassanzadeh, 2017; Hu et al., 2022a; Hu et al., 2022b; Hu et al., 2023a; Hu et al., 2023b; Thakkar et al., 2022). Despite its lethal effects (LC_50_), the toxic potential of MTP in adults was assessed by examining blood, gills, liver, intestine, gonads (testis and ovary), and brain, as well as evaluating the growth of the fish (length and weight) (Table 4; Supplementary Table S1). It was observed that MTP exposure led to an increased frequency of micronuclei in RBCs, decreased EROD activity in the gills, and enhanced LPO in the gills (50 mg/L; 96 h) (de Carvalho Penha et al., 2021). The growth of the fish (length and weight) with MTP (1–10 μg/L) was found to be sex-specific, with a significant increase observed only in female fish (Hassanzadeh, 2017; Hu et al., 2022b). Moreover, MTP induced hepatocellular vacuolization. Furthermore, the lipid metabolism dynamics across blood, liver, and gut were significantly dysregulated; in male fish, cortisol concentration in the liver was increased, while in female fish, synthesis and conjugation of primary bile acids were inhibited (Hu et al., 2022a). Moreover, MTP caused the degradation of E2 and retinoic acid occurred in the liver (Hu et al., 2022a). Although the gut microbiota was disrupted, sex-specific alteration was observed in the intestine related to goblet cell density, TJP2 expression, and serotonin concentrations, which were increased in male fish and decreased in female fish (Hu et al., 2022b). CAT activity in the intestine was activated after MTP exposure, which can contribute to the removal of oxidative free radicals generated due to stress (Hu et al., 2022b).

The ED effects of MTP were also investigated in both male and female zebrafish (Hu et al., 2023b). The GSI (gonad weight relative to body weight) was increased in both male and female fish after 4 weeks of MTP exposure in a concentration-dependent manner (Hu et al., 2023b). In contrast, a concentration-dependent decrease in GSI was observed in male fish after 21 days of exposure (Hassanzadeh, 2017). Gametogenesis in the testis and ovary was blocked by MTP (Table 4; Supplementary Table S1). Histological investigations indicated testicular atrophy, multinucleated gonocytes, impaired germ cells, spermatogonial proliferation, Leydig cell hyperplasia, interstitial fibrosis, and apoptosis of Sertoli cells (Hassanzadeh, 2017). Moreover, the toxic effects were intergenerationally transmitted to the embryos (enhanced mortality, precocious hatching, and elevated heart rates) when the parental zebrafish were exposed to MTP (Hu et al., 2023b). The sex hormones in the blood (E2, T, and 11-KT) were consistently lowered by MTP exposure. Gene expression analysis evidenced the disruption of several HPG-axis genes that have the potential to impair vitellogenesis during oogenesis (Hu et al., 2023b).

In adults, the neurotoxic effect of MTP was sex-specific (Hu et al., 2023a). The cranio-somatic index (CSI) in female fish was enhanced in a concentration-dependent manner (Table 4; Supplementary Table S1). In the male brain, concentration-dependent enhancement in glutamate content and upregulation of glutamate receptors occurred after MTP exposure. In female fish, the proteins responsible for synapse formation and regeneration were reduced by MTP, potentially blocking synaptic neurotransmission (Hu et al., 2023a). Moreover, in both male and female fish, the downregulation of corticotropic releasing hormone (*crh*) and corticotropic hormone binding protein (*crhbp*) genes decreased the cortisol concentration in the blood (Hu et al., 2023a). A significant increase in lipopolysaccharide (LPS) content in the male brain and upregulation of the blood–brain barrier proteins in the female brain further support the sex-specific neurotoxic effects of MTP in adult zebrafish (Table 4; Supplementary Table S1).

Overall, our systematic review on MTP was restricted to only 24 articles, with 79% (19 articles) focusing on studies involving zebrafish (Table 2). The other three fish species (common carp, fathead minnows, and Japanese medaka) are represented by one article each. Moreover, we did not find any *in vivo* study focused on the effects of MTP in rainbow trout; an *in vitro* study using the primary hepatocytes of rainbow trout was performed using fractionated effluents containing MTP (Grung et al., 2007). Furthermore, the effects of MTP were studied in the embryos, larvae, and adults (male and female) of zebrafish; in other fish species, the studies were restricted only to embryos (common carp), larvae (fathead minnows), or adults (Nile Tilapia and Japanese medaka). Despite limitations, our studies (systematic review) indicate that MTP has the potential to alter the crucial developmental and physiological landmarks in these fish that could affect the behavior of the animal.

### 3.2 ETP

ETP is one of the most important food preservatives used in various types of food, including fresh fruits, vegetables, flavoring, sauce, beverages, and pastry. Consequently, the highest concentration of ETP detected in surface water, soil, indoor air, and indoor dust is 147 ng/L, 5.10 ng/L, 4.0 ng/m^3^, and 3,110 ng/g, respectively (Rudel et al., 2003; Nunez et al., 2008; Ramaswamy et al., 2011). In humans, ETP is detected in urine (564 ng/mL), serum (20.8 ng/mL), and seminal fluid (5.65 ng/mL) (Frederiksen et al., 2011). Moreover, ETP has also been detected in cord blood and placental tissue, indicating its ability to cross the placental barrier and pose a risk to fetal development (Jimenez-Diaz et al., 2011; Pycke et al., 2015; Finot et al., 2017).

#### 3.2.1 Fathead minnow

The effects of ETP on fathead minnows were investigated by Dobbins et al. (2009). The 48 h LC_50_ value of the 1dpf larvae was 34.3 mg/L (86.05 µM). The LOEC for larval growth and hazard quotient were 17 mg/L and 7.8 × 10^−6^, respectively (Table 3). The toxic effects of ETP on embryos and adults of fathead minnows are yet to be investigated; moreover, the effects on reproduction, neurobehavior, and other physiological endpoints remained unexplored in embryos, larvae, and adults.

#### 3.2.2 Japanese medaka

The effects of ETP were studied in Japanese medaka larvae (Yamamoto et al., 2011). The 96 h LC_50_ value of the larvae (10 dph) was 14 mg/L (86.05 µM) (Table 3; Supplementary Table S1). However, the effects on embryo–larval development, reproduction, and neurobehavior are yet to be investigated.

#### 3.2.3 Nile tilapia

The study on the effects of ETP on Nile tilapia is very limited; only adult male fish were used for toxicological assessments. The 48 h LC_50_ value on adult male fish was 24.08 mg/L (144.91 µM) (Silva et al., 2018). Furthermore, adult male fish were exposed to ETP (4 mg/L) for 6 and 12 days, and the effects on LPO markers (GSH and MDA content) and oxidative stress-related enzymes (SOD, CAT, GPx, and GR) in the gill and liver were evaluated (Table 5; Supplementary Table S1). It was observed that GSH and MDA contents remained unaltered in the gills, while GSH decreased at 6 days and increased at 12 days in the liver, and MDA decreased in the liver only in fish exposed for 12 days (Silva et al., 2018). GPx activity increased in the gills after 6 and 12 days, while SOD activity increased in both liver and gills only at 12 days. CAT activity increased in the liver at 6 days, and GR activity increased in the liver after 12 days of exposure (Silva et al., 2018). The effects of ETP during embryo–larval development or in larvae and adult female fish are yet to be investigated. Moreover, the effects of ETP on the reproduction and neurobehavior of Nile tilapia need to be studied.

**TABLE 5:** Effects of ETP on fish.

Fish	Author	Toxicological endpoint	Reproductive/endocrine-related endpoint	Neurobehavioral endpoint
Fathead minnows (1 dph larvae)	Dobbins et al. (2009)	i) The 48 h LC_50_ value was 34.3 mg/L ii) LOEC for larval growth = 17 mg/L iii) Hazard quotient for larval growth = 7.8 × 10^−^ ^6^		
Japanese medaka (10 dph larvae)	Yamamoto et al. (2011)	i) The 96 h LC_50_ value in 10 dpf larvae was 14 mg/L		
Nile tilapia (adult male fish)	Silva et al. (2018)	i) The 48 h LC_50_ value in adults was 24.08 mg/L ii) Lipid peroxidation (GSH and MDA contents) in the gills and liver lacks consistency (gills, unresponsive; liver GSH increased after both 6 and 12 days; MDA remained unaltered in the gills and decreased in the liver after 12 days of exposure. The CAT and GR remained unaltered in the gills; GPx increased in gills after 6 and 12 days; SOD in the gills increased only after 12 days; in the liver, GPx and SOD did not alter; CAT increased after 6 days and GR increased after 12 days		
Rainbow trout (immature)	Pedersen et al. (2000)		i) Serum VTG induced by ETP in a dose-dependent manner	
Zebrafish (embryo-larval development) (exposure period, 96 h)	Merola et al. (2020a; 2021), Fan et al. (2022)	i) 96 h LC_50_ = 20.86–28.74 mg/L ii) TC = 20.63 mg/L iii) Developmental abnormalities like reduction in blood circulation, blood stasis, pericardial edema, deformed heart loop and irregular heartbeats, misshaped yolk, deformed notochord and pectoral fins, and lack of detachment of the tail bud from the yolk sac were observed in a concentration-dependent manner iv) Transcriptomic analysis indicated that 1,302 genes were upregulated and 2,482 genes were downregulated v) Disrupted retinoic acid signaling pathways and inhibited gene expression related to myocardial contraction and orientation disturbance in heart tubes		i) Abnormalities including trembling of the head, pectoral fins, and spinal cords and circling behavior were observed in larvae at 72 hpf exposed to 10 mg/L ETP ii) Increased thigmotaxis and did not influence the visual startle response and the photic synchronization of the circadian rhythm
Zebrafish (embryo-larval development) (exposure period, 120 hpf)	Liang et al. (2022; 2023a, 2023b); Tran et al. (2023)	i) LC_50_ = 32.57 mg/L ii) TEI = 1.3 iii) Cardiac edema and tail curvature increased significantly in a concentration-dependent manner iv) Gene expression analysis showed that 414 genes were DEGs, of which 352 genes were upregulated and 67 genes were downregulated	i) T3 and T4 concentrations reduced significantly ii) Several genes of the HPT-axis were upregulated (*tshβ* and *trα*), and others were downregulated (*pax* and *ttr*) iii) The vtg level increased, while the T level decreased iv) Several genes of the HPI-axis (*gr*, *mr*, *pmoc*, *crhbp*, and *crhr2*), HPG-axis (*gnrh1*, *gnrh2*, *fshr*, *lhβ*, and *ar*), and steroidogenesis pathways (*cyp11a*, *cyp19a*, *cyp19α*, *3βhsd*, and *17βhsd*) were affected after exposure to ETP	i) The neuroendocrine parameters including AChE activity, ACTH levels and cortisol levels, were altered after ETP exposure ii) Swimming behavior (total distance traveled and swimming velocity) reduced

#### 3.2.4 Rainbow trout

The effects of ETP were evaluated in juvenile rainbow trout (80–120 g) only after injecting 100 and 300 mg/kg of ETP to the fish on days 0 and 6 of the experiment. The serum VTG levels on days 0, 6, and 12 showed a dose-dependent increase compared to the control (Pedersen et al., 2000). The study showed that ETP is an estrogenic endocrine disruptor (EED) targeting estrogen-dependent mechanisms (Table 5 and Supplementary Table S1). Embryos and adult rainbow trout were not used in ETP studies. Moreover, studies on toxicological and neurobehavioral endpoints are yet to be investigated in this fish species.

#### 3.2.5 Zebrafish

The ETP effects on zebrafish were evaluated only during embryo–larval development (Table 5 and Supplementary Table S1) after exposing the embryos either for 96 h (0.1–100 mg/L) (Merola et al., 2020a; 2021; Fan et al., 2022) or 120 h (0.17–49.85 mg/L) (Liang et al., 2022; 2023a; 2023b; Tran et al., 2023). The 96 h LC_50_ value ranged from 20.86 to 28.70 mg/L (Merola et al., 2020a; Fan et al., 2022) although the BMD (EFSA Scientific Committee et al., 2017; Sant and Timme-Laragy, 2018) was 10.8–17.4 mg/L (Merola et al., 2020a) and the teratogenic concentration (TC) was 20.63 mg/L (Fan et al., 2022); 85% of the embryos died at 96 hpf if the concentration of ETP was 30 mg/L (Table 5; Supplementary Table S1). Hatching was concentration- and time-dependent (Merola et al., 2020a). Developmental abnormalities (reduction in blood circulation, blood stasis, pericardial edema, deformed heart loop, irregular heartbeats, misshaped yolk, deformed notochord and pectoral fins, and lack of detachment of the tail bud from the yolk sac) were induced in a time- and concentration-dependent manner (Merola et al., 2020a; Fan et al., 2022). Behavioral abnormalities (trembling of the head, pectoral fins, and spinal cords and circling behavior) were observed in larvae (72 hpf) exposed to 10 mg/L (Supplementary Table S1; Merola et al., 2020a). Other behavioral tests (thigmotaxis, visual startle response, and photic synchronization) on 4–6 dpf larvae (exposed to 0.05–5 mg/L ETP until 96 hpf) showed that ETP increased thigmotaxis in a concentration-dependent manner (Merola et al., 2021); however, no significant difference was observed in the visual startle response and the photic synchronization of the circadian rhythm (Table 5; Supplementary Table S1). Transcriptomics analysis indicated that ETP resulted in 1,302 upregulated and 2,482 downregulated genes (96 hpf). Further analysis indicated that ETP disrupted retinoic acid signaling pathways and inhibited gene expression related to myocardial contraction and orientation disturbance in heart tubes (Fan et al., 2022).

When embryos were exposed to ETP (0.17–48.85 mg/L) until 120 hpf, the LC_50_ value was 32.57 mg/L (Tran et al., 2023). The body length and heart rate were reduced, and the malformation rates (cardiac edema and tail curvature) were increased in a concentration-dependent manner. Furthermore, ETP (3.32–16.61 mg/L; 120 hpf) decreased T3 and T4 contents of the larvae in a concentration-dependent manner (Liang et al., 2022). Gene expression analysis related to the HPT axis indicated the upregulation of *tshβ* and *trα*, while *pax* and *ttr* contents were reduced (Liang et al., 2022). An analysis of estrogen-sensitive endpoints (VTG, E2, and T) (Liang et al., 2023a), the neuroendocrine parameters (AChE, ACTH, and cortisol), and the genes related to hypothalamus–pituitary–interrenal (HPI) axis indicated disrupting effects of ETP on ED and neurobehavior of the larvae (Liang et al., 2023b). Furthermore, the behavioral response (120 hf) resulted in hyperactivity (Supplementary Table S1); the total distance traveled and the mean velocity of the larvae (120 hpf) were reduced (Liang et al., 2023b). Gene expression analysis (ETP exposed for 120 h) showed that 414 genes were differentially expressed genes (DEGs); 352 genes were upregulated, and 64 genes were downregulated (Tran et al., 2023). Moreover, the expression of several HPI axis genes (*gr*, *mr*, *pmoc*, *crhbp*, and *crhr2*), HPG axis genes (*gnrh1*, *gnrh2*, *fshr*, *lhβ*, and *ar*), and steroidogenesis pathways (*cyp11a*, *cyp19a*, *3βhsd*, *and 17βhsd*) were disrupted by ETP (120 hpf).

Taken together, our studies on ETP were restricted to only 11 articles, of which 7 (63.63%) involved zebrafish (Table 2). Other four fish species (fathead minnows, Japanese medaka, Nile tilapia, and rainbow trout) were represented by one article each. Common carp was yet to be used for ETP studies. In zebrafish, only embryo–larval development was emphasized to toxicity (mortality and lethal effects), ED effects (HPG-, HPI-, and HPT-axis), and neurobehavioral disorders (movement disorders and AChE activity); in other fish, the studies were restricted to only larvae (fathead minnows and Japanese medaka), juveniles (rainbow trout), or adults (Nile Tilapia). Our studies showed that ETP has the potential to alter the crucial developmental and physiological landmarks that could disrupt the endocrine functions and the neurobehavior of the fish.

### 3.3 PPP

PPP, an n-propyl ester of p-hydroxybenzoic acid, is both a naturally occurring and industrially produced substance used as a preservative and widely detected in cosmetic formulations and often detected in aquatic environments (Gonzalez-Marino et al., 2011). According to the Danish Ministry of Environment, PPP was found in 38% of analyzed cosmetics and personal care products, with levels ranging from 0.01% to 0.32% (Rastogi et al., 1995; Wei et al., 2021). PPP has also been detected in human urine, breast milk, cord blood, placenta, seminal plasma, adipose tissue, and even in breast cancer tissues (Petric et al., 2021). It has bactericidal and fungicidal effects. Moreover, PPP can modify the seizure threshold in zebrafish when administered at low concentrations (Pisera-Fuster et al., 2017). Furthermore, in zebrafish, PPP at lower doses increased the latency to spams induced by pentylenetetrazole (PTZ) (Pisera-Fuster et al., 2017). Available reports indicate that PPP is present at a concentration of 20,000 ng/L in wastewaters, 3,142 ng/L in freshwater, and 23 ng/L in bottled drinking water (Carmona et al., 2014; Haman et al., 2015). The water samples from 11 different WWTPs in Sweden containing PPP (mean concentration of 0.11 ng/L) induced pericardial and yolk sac edema in zebrafish embryos (Golovko et al., 2021).

#### 3.3.1 Common carp

Embryos exposed to PPP (0.1–100,000 μg/L) for 96 h induced 100% mortality in 100,000 μg/L concentrations (Table 6; Supplementary Table S1). Hatching delay was observed in embryos exposed to 50–5,000 μg/L. Gene expression analysis indicated a significant downregulation of *cyp19b* and *gst1* mRNAs in the embryos exposed to 0.1 μg/L PPP, not in 100 μg/L PPP (Medkova et al., 2023). Although the study is restricted only to embryonic development, it indicates that PPP is toxic to common carp and can impair steroidogenesis and lipid peroxidation at higher concentrations. Studies on larvae and adults are yet to be investigated.

**TABLE 6:** Effects of PPP on fish.

Fish	Author	Toxicological endpoint	Reproductive /endocrine-related endpoints	Neurobehavioral endpoint
Common carp (embryos)	Medkova et al. (2023)	i) All embryos died in 100 mg/L concentrations at 96 hpf ii) Delayed hatching in a concentration-dependent manner iii) Expressions of *cyp19a* and *gst1* mRNAs were downregulated by PPP in a nonlinear fashion		
Fathead minnows (1 dph larvae)	Dobbins et al. (2009)	i) 48 h LC_50_ was 9.7 mg/L for PPP and 17.5 mg/L for i-PPP ii) LOEC for larval growth was 2.5 mg/L for PPP and 9.0 mg/L for i-PPP iii) The hazard quotient on the growth of the larvae was 3.1 × 10^−^ ^5^ for PPP and 2.8 × 10^−^ ^5^ for i-PPP		
Japanese medaka (10 dph larvae)	Inui et al. (2003); Yamamoto et al. (2011); Gonzalez-Doncel et al. (2014)	i) Survivability of the embryos was affected in a concentration-dependent manner. No effect was observed on embryos exposed to PPP <1 mg/L ii) Dilation of the gall bladder induced by PPP iii) EROD activity remained unaltered iv) The calculated 96 h LC_50_ value for n-PPP and i-PPP in 10 dpf larvae was 4.6 mg/L and 4.5 mg/L, respectively	v) Plasma VTG concentration increased *vi) vtg1*, *vtg2*, *chgL*, *chgH*, *esr1*, and *esr2* mRNAs in the liver were upregulated in a concentration-dependent manner vii) Androgen receptor mRNA (*ar*) remained unaltered	
Nile tilapia (adult male fish)	Silva et al. (2018)	i) 48 h LC_50_ in adults was 17.36 mg/L (14.63–20.61 mg/L) ii) Oxidative stress (enzyme activities of CAT, GPx, GR, and SOD) and LPO (GSH and MDA content) determined in the gill and liver of adult male fish after exposing to PPP (4 mg/L) for 6 and 12 days showed inconsistent and nonlinear alterations		
Rainbow trout (immature)	Pedersen et al. (2000); Bjerregaard et al. (2003)		i) Sexually immature rainbow trout exposed to PPP either orally, by immersion, or by intraperitoneal injection induced plasma VTG content in a concentration/dose-dependent manner ii) PPP accumulated in the liver and muscle of fish when exposed to PPP by immersion	
Zebrafish (embryo-larval development) (exposure period, 96 h)	Torres et al. (2016); Perugini et al. (2020); Lite et al. (2022); Medkova et al. (2023); Merola et al. (2024)	i) Calculated 96 h LC_50_ was 3.98 mg/L (22.08 µM) ii) Enlarged and misshaped yolk sac, reduction in head size and swim bladder; abnormal eyes and tail iii) Pericardial edema and concentration-dependent reduced heart rates iv) Decrease in neutral lipid metabolism from yolk and alteration in phospholipid metabolism v) 100% mortality of the embryos was observed at 10–100 mg/L vi) Concentration-dependent hatching delay vii) Significant increase in ROS and LPO levels with suppressed SOD, CAT, GPx, enzyme activity, and GSH content viii) Upregulation of *hsp70l*, *cyp17a1*, and *cyp19a1a* and downregulation of *hsp90* genes in a concentration-dependent manner; however, upregulation of *gstp2* was nonlinear		i) Trigger anxiety-like behavior in larvae (6 dpf) ii) Thigmotaxis was decreased in larvae (5/6 dpf) exposed to 1,000 μg/L during development and increased in juveniles exposed to 10 μg/L (30–60 dpf) iii) The expression of *shank3a* was repressed in larvae and juveniles (60 dpf), while *gad1b* was repressed only in larvae iv) AChE activity was inhibited, and NO production increased in larvae (6 dpf) v) Hyperexcitability
Zebrafish (embryo-larval development) (exposure period, 120 hpf)	Bereketoglu and Pradhan, (2019); Liang et al. (2021, 2023a, 2023b); Tran et al. (2023)	i) The 120 h LC_50_ value was 11. 14 mg/L (61.8 µM) ii) Concentration-dependent decrease in body length, heart rates, hatching rates, and malformation (spinal curvature, pericardial edema, and yolk sac edema) iii) 181 genes are upregulated and 134 genes are downregulated iv) Concentration-dependent downregulation of the expression of genes related to oxidative stress such as *nrf2*, *gst*, and *sod1* and upregulation of *hsp70* and *mt1*; however, *keap1* and *mgst* showed nonlinear downregulation v) Concentration-dependent upregulation of the genes related to apoptosis, cell proliferation, and DNA damages (*bax*, *p38*, *tnfα*, and *xpc*); downregulation of genes (*bcl2, casp3a*, *dap3*, *p21*, *rad51*, *apex1*, and *il8*), which showed a nonlinear trend vi) The concentration-dependent upregulated fatty acid metabolism-related genes are *fasn,* and *lipc*, while the downregulated genes are *apoab*, *apoeb*, *apoa4*, and *idlr*. Only *lpl* gene downregulated in a nonlinear fashion	i) Significant decrease in ACTH and increase in cortisol levels ii) Concentration-dependent nonlinear reduction in T and no significant alteration in E2 levels iii) Concentration-dependent decrease in T3 and T4 levels iv) Vtg enhancement occurred at lower concentrations (2 µM; no significant difference at 5 and 10 µM concentration) v) Nonlinear alteration in the expression of *17β-hsd*, *3β-hsd*, *cyp19a*, *cyp17*, *cyp11a*, *lhβ, fshr*, *fshb*, *gnrh2*, *gnrh3*, *gnrhr1*, *gnrhr4*, and *hmgrα* genes vi) Downregulation of mRNA levels of *gr, mr, crhbp*, and *crhr2* vii) Concentration-dependent upregulation of *esr2a* and downregulation of *ar* mRNAs viii) Nonlinear increase in the expression of *tshβ*, *tg*, *nis*, *dio1*, *nkx2.1*, *tir*, and *ugt1ab; trα and trβ* exhibited nonlinear downregulation in the expression of *pax8*	i) Total distance traveled and mean velocity of the larvae reduced in a concentration-dependent manner ii) No significant difference in the locomotor activity in the light and dark phases
Zebrafish (embryos; exposure, period 24 hpf)	Merola et al. (2022)	i) At 8 hpf, the 27-OH concentrations in PPP-exposed embryos (10 μg/L) were higher than those of controls ii) At 24 hpf, 24-hydroxycholesterol (24-OH) was higher in PPP-exposed embryos (10–1,000 μg/L) than in controls		
Zebrafish (20 dpf; juvenile)	Mikula et al. (2006a, b, 2009)	i) No effect on length and weight of the fish when administered orally for 45 days	i) Oral exposure (administered orally via food) for 20 days did not alter the vtg content in the whole body of the fish ii) Female-biased sex ratio after 45 days of feeding iii) Whole body vtg significantly reduced when exposed to PPP by immersion	
Zebrafish (3-month-old adults)	Pisera-Fuster et al. (2017)			i) As an antiepileptic compound, PPP (9 μg/L; 96 h) has the ability to modify seizure threshold (PTC-induced) in adult zebrafish

#### 3.3.2 Fathead minnows

The effect of PPP (n-PPP) and i-PPP on fathead minnows was investigated by Dobbins et al. (2009) using 1-dph larvae (Table 6; Supplementary Table S1). The 48 h LC_50_ value was 9.7 mg/L for PPP and 17.5 mg/L for i-PPP. The LOEC and hazard quotient were 2.5 mg/L and 3.1 × 10^−5^, respectively; for i-PPP, the LOEC was 9.0 mg/L and the hazard quotient was 2.8 × 10^−5^. Although the study showed that PPP is a more potent toxicological compound than i-PPP, other studies on embryo–larval development and in adults targeting ED or neurobehavior are yet to be investigated.

#### 3.3.3 Japanese medaka

The effects of PPP on Japanese medaka were evaluated in embryos, larvae, and adult male fish (Table 6; Supplementary Table S1). Moreover, the toxic potential of i-PPP was also evaluated in the larvae of this fish (Yamamoto et al., 2011). The embryos (24 hpf) were exposed to PPP (40–4,000 μg/L) for 10 dpf, and the effects during development as embryos (76–316 hpf), eleutheroembryos (13 dpf), and larvae (28 and 43 dpf) were evaluated. It was observed that the effects of PPP on general embryogenesis, embryonic ethoxyresorufin-O-deethylase (EROD) activity, gall bladder morphology, hatching, and swimming activity were dependent on the developmental stages of the fish (Gonzalez-Doncel et al., 2014). The PPP did not show any toxic effects on the embryos or larvae when exposed to <1,000 ng/L. However, survivability was affected by PPP when exposed to ≥4000 μg/L for eleutheroembryos and ≥1000 μg/L for larvae (Gonzalez-Doncel et al., 2014). PPP concentrations (40–4,000 μg/L) did not affect hatching or induce mortality in embryos during exposure although the dilation of gall bladder was observed in embryos throughout the development, even at lower concentrations of PPP (≥400 μg/L). EROD activity remained unaltered (Gonzalez-Doncel et al., 2014).

Larvae (10 dph) were exposed to n-PPP and i-PPP for 96 h, and the toxic potential of PPP was evaluated (Table 6; Supplementary Table S1). The 96 h LC_50_ value was 4.9 mg/L (27.19 µM) and 4.5 mg/L (24.97 µM) for PPP and i-PPP, respectively (Table 3). Moreover, adult male fish (2.5 months old) were exposed to PPP (0.055–55 mM) for 1 week and used for estimation of VTG in the plasma and the expression of several estrogen-sensitive genes, including *vtg1*, *vtg2*, *chgL*, *chgH*, *esr1*, *esr2*, and *ar*. It was observed that the VTG levels in plasma and mRNAs of the hepatic genes (*vtg1*, *vtg2*, *chgL*, *chgH*, *esr1*, *and esr2*) in the liver were enhanced in a concentration-dependent manner (Inui et al., 2003) although *ar* mRNA remained unaltered (Yamamoto et al., 2011). The effects of PPP on neurobehavior in Japanese medaka are yet to be investigated.

#### 3.3.4 Nile tilapia

Adult fish were exposed to PPP (3.1–24.8 mg/L) for 48 h, and the calculated LC_50_ value was 17.36 mg/L (Table-3). Furthermore, adult male Nile Tilapia were exposed to PPP (4 mg/L) and a mixture of MTP (6 mg/L) and PPP (1.7 mg/L) for 6 and 12 days and used for lipid peroxidation (GSH and MDA content) and oxidative stress-related enzyme (SOD, CAT, GPx, and GR) assays in the gills and liver (Table 6; Supplementary Table S1). GSH remained unaltered in the gills and decreased in the liver after 6 days but increased after 12 days. MDA remained unaltered in both the liver and gills after 6 and 12 days; for mixtures (6 mg/L MTP and 1.7 mg/L PPP), both GSH and MDA in the gills remained unaltered after 6 and 12 days, while in the liver, GSH decreased after 6 days and increased after 12 days, and MDA decreased in fish exposed to mixtures after 12 days (Silva et al., 2018). CAT activity remained unaltered in both the liver and gills (PPP 4 mg/L) after 6 or 12 days. SOD increased only in the gills after 12 days. GPx and GR activities increased only in the gills after 6 days. For mixtures, CAT activity remained unresponsive, while SOD activity increased in the gills after 6 days but decreased in the liver after 12 days. GPx activity increased in the gills after 12 days and in the liver after 6 days, and GR activity decreased in the gills after 12 days and increased in the liver after 12 days. The study indicates that PPP can mediate toxic stress by modulating oxidative stress in adult fish; however, the effects of PPP on embryo–larval development, reproduction, and neurobehavior of Nile tilapia are yet to be investigated.

#### 3.3.5 Rainbow trout

The effects of PPP were evaluated in sexually immature rainbow trout (Table 6; Supplementary Table S1). Juvenile rainbow trout (80–120 g) were injected with PPP (100 and 300 mg/kg), and the plasma was collected on days 0, 6, and 12 and used for VTG assays (Pedersen et al., 2000). A dose-dependent increase was observed in plasma VTG in fish injected with either 100 or 300 mg/kg of PPP (Table 6; Supplementary Table S1). In sexually immature rainbow trout, PPP was administered either orally with a concentration of 7–1830 mg/kg every other day until 10 days or by immersion (50–225 μg/L) for 12 days. The VTG in both experiments increased significantly in a dose-dependent manner (Bjerregaard et al., 2003). The ED_50_ values for increase in plasma VTG were 35, 31, and 22 mg/kg on days 3, 6, and 11, respectively. However, in immersion experiments, the increase in plasma VTG was concentration-dependent (Bjerregaard et al., 2003). Moreover, the accumulation of PPP in the liver was found to be 6,700 μg/g of the liver and 870 μg/g of muscle. The half-life for PPP in the liver of rainbow trout is 8.6 h, and in muscle, it is 1.5 h (Bjerregaard et al., 2003). Other than these studies, the effects of PPP in embryo-larval development, reproduction, and neurobehavior of rainbow trout are yet to be investigated.

#### 3.3.6 Zebrafish

In zebrafish, the embryo–larval development and the larvae were used for the assessment of the toxic potentials of PPP, while adults were used to evaluate PPP as an anticonvulsant compound (Table 6; Supplementary Table S1). The embryos were exposed to a wide range of PPP (0.1–100,000 μg/L) by immersion and evaluated at various time periods (8–60 dpf) targeting developmental (survivability, yolk, heart, eye, tail, hatching, body length, ROS, LPO, and apoptosis), endocrinological (HPG-, HPI-, and HPT-axis and vtg), and neurobehavioral (AChE, anxiety, and thigmotaxis) endpoints. The larvae (20 dph) were exposed to PPP (100–900 μg/L) either by immersion or orally via food (500–200 mg/kg) for 20 or 45 days, and VTG content (20 days exposure) and the sex ratio (45 days exposure) of the fish were evaluated (Mikula et al., 2006a; b; 2009). The adults (3-month-old) were used for the evaluation of PPP as an antiepileptic drug on a zebrafish model of PTZ-induced seizure (Pisera-Fuster et al., 2017).

The LC_50_ value was determined in embryo–larval assays (Table 3) after exposing the embryos either for 96 hpf or 120 hpf of development (Table 6; Supplementary Table S1). The 96 h LC_50_ value was 3.98 mg/L (22.08 µM), and the 120 h LC_50_ value was 11.14 mg/L (61.8 µM) (Table 3). The toxic effects of PPP (10 and 10,000 μg/L) were also evaluated in *ovo* using embryos prior to 96 hpf of development (8 hpf, gastrula; 24 hpf, prim5; 32 hpf, pharyngula; and 80 hpf larvae, protruding mouth) (Torres et al., 2016; Merola et al., 2022). PPP exposure enhanced mortality and induced abnormalities in the yolk, heart, head, eyes, and tail and delayed hatching in a concentration-dependent manner (Torres et al., 2016; Medkova et al., 2023). PPP (8.5 mg/L or higher) significantly affects embryos to achieve 75% epiboly at 8 hpf; embryos exposed to 10 mg/L at 32  hpf showed an increase in yolk sac and tail abnormalities and a decrease in heart rates (Table-6; Supplementary Table S1). Embryos exposed to PPP (10–1,000 μg/L) for 8–24 h did not show any significant alteration in the total oxysterol (OH) content, a metabolic byproduct of cholesterol, compared with control groups (Supplementary Table S1); however, the concentration of 27-hydroxysterol (27-OH) was higher in 8 hpf embryos (exposed to 10 μg/L), and there was an increase in 24-OH at 24 hpf (10–1,000 μg/L) (Merola et al., 2022). At 80 hpf, significant abnormalities are observed in the eyes, head, pericardial edema, tail, yolk sac, heart rates (reduced), body length (reduced), and hatching (delayed) (Table 6; Supplementary Table S1).

The most common observed effects in 96 hpf-exposed embryos were enlarged and misshaped yolk sac, hyperexcitability, reduction in head size and swim bladder, deformed neurocranium, reduced heart rates, delayed hatching, bent spine, and an alteration in lipid metabolism in body and yolk sac in a concentration-dependent manner (Table-6; Supplementary Table S1). The activities of CAT, GPx, GST, SOD, and GSH and LPO and induction of apoptosis in the head region of zebrafish indicate that PPP alters the defense mechanisms of zebrafish larvae (Lite et al., 2022). AChE activity reduced significantly and enhanced NO in larvae exposed to PPP (Table 6; Supplementary Table S1). Gene expression analysis (0.1–1,000 μg/L) in 96 hpf larvae showed upregulation of *hsp70l*, *gstp2*, *cyp17a1*, *cyp19a1a*, and *cyp19a1b* (increasing tendency) and downregulation of *hsp90* (only in 0.1 μg/L) and *shank3a* and *gad1b* (10 μg/L) in a concentration-dependent manner (Medkova et al., 2023; Merola et al., 2024). Furthermore, gene expressions and proteomic analysis in the brains of 60 dpf larvae (juvenile) showed that the expressions of *shank3a* and *gad1b* were repressed. Neurobehavioral analysis (open-field behavior, startle response, and circadian rhythmicity) was conducted on 4–6 dpf larvae, revealing anxiety-like neurobehavioral disorders (spending less time in light and a significant increase in the number of light–dark transitions) (Lite et al., 2022; Merola et al., 2024). Moreover, behavioral and cognitive impacts on sociability, cerebral functional asymmetry, and thigmotaxis were also examined in juvenile fish at 30 and 60 dpf of development after exposing embryos to PPP during embryogenesis (96 hpf) (Table 6; Supplementary Table S1). Moreover, the brains of the 60 dpf larvae were used for proteomics and gene expression analysis (Merola et al., 2024). It was observed that thigmotaxis was decreased in larvae exposed to 1,000 μg/L PPP during development (96 hpf) and increased with exposure to the low concentration of PPP (10 μg/L). However, the anxiety-like behavior increased in 4 dpf larvae, suggesting that PPP is an anxiogenic neuroactive compound affecting brain development in zebrafish (Lite et al., 2022). This also indicates that early-life exposure (96 hpf) to PPP promotes persistent developmental and neurobehavioral alterations (Merola et al., 2024).

Zebrafish embryos were exposed to a wide range of PPP (36–54,000 μg/L) for 120 h, and the toxic potentials on development, endocrine, and neurobehavior were evaluated (Table 6). As expected, the developmental disorders (mortality, yolk sac edema, cardiac edema, spinal defects, delay in hatching, reduction in body length, and heart rates) were disrupted by PPP in a concentration-dependent manner (Table 6; Supplementary Table S1). Moreover, the VTG content increased significantly in larvae exposed to PPP (2 µM), while T significantly decreased in larvae (2–10 µM) when compared with controls. The T3 and T4 concentrations were reduced (0.18–9.0 mg/L) (Liang et al., 2022). The behavioral analysis of 5 dpf larvae did not show any significant difference in either dark or light phases although the total distance covered by swimming and the swimming velocity of the larvae were reduced after PPP treatment (10 µM).

The gene expression analysis showed 315 DEGs, of which 181 genes were upregulated and 134 genes were downregulated (Tran et al., 2023). Among them, the genes involved in oxidative stress responses (*nrf2*, *keap1*, *gst*, *mgst*, and *sod1*) were downregulated; however, *hsp70* and *mt1* were upregulated in a concentration-dependent manner (Bereketoglu and Pradhan, 2019); the expression of genes belonged to cell cycle, DNA damage, and inflammation (*casp3a*, *dap3*, and *bcl2)* was downregulated, while *bax* mRNA levels increased (Table-6; Supplementary Table S1). Furthermore, the expressions of cyclin-dependent kinase inhibitor 1A (*p21*) and growth arrest and DNA damage-inducible alpha (*gadd45a*) were downregulated (10 µM PPP); the expression of *tnfα* (the gene belonging to the immune system) was increased in a concentration-dependent manner, while *il8* significantly reduced in larvae exposed to 1 µM PPP. The apolipoprotein genes (*apoab*, *apoeb*, and *apoa4*) were downregulated; the fatty acid synthesis gene (*fasn*) and the lipase gene *lipase, hepatic* (*lipc*) were upregulated in a concentration-dependent manner. Genes in the HPT-axis indicated that among 15 genes, upregulation (*tshβ*, *tg*, *nis*, *dio1*, *nkx2.1*, *ttr*, *trα*, *and trβ*) and downregulation (*pax8*) of several genes, although nonlinear, were modulated by PPP (Table 6; Supplementary Table S1). Genes of the HPG-axis indicated that among 19 genes, *gnrh2*, *gnrh3*, *gnrhr1*, *gnrhr4*, *fshβ*, *ar*, and *esr2a* mRNAs were downregulated, while *fshr* and *lhβ* mRNAs were upregulated (Bereketoglu and Pradhan, 2019; Liang et al., 2023a). Moreover, the expression of *3βhsd* and *17βhsd* showed an increasing expression although it was inconsistent (Liang et al., 2023a). Genes of the HPI-axis showed downregulation of *mr*, *crhbp*, *gr*, and *crhr2* mRNAs, while the expression of *pmoc* was inconsistent (Liang et al., 2023b). All these studies suggest that PPP has potential ED effects during the embryo-larval development of zebrafish (Bereketoglu and Pradhan, 2019).

Juvenile zebrafish (20 dph) were exposed to PPP (0.1–0.9 mg/L) for 20 days by immersion (Mikula et al., 2006a; Mikula et al., 2006b) or orally (500–2000 mg/kg) via food for 20 and 45 days (Mikula et al., 2009). PPP exposed through immersion reduced the VTG content in the whole body of the fish (Mikula et al., 2006a; Mikula et al., 2006b). When PPP was fed to the larvae, the VTG content of the whole body after 20 days of treatment remained unaltered (Table 6; Supplementary Table S1). However, the sex ratio of the fish fed with PPP for 45 days showed a female-biased sex ratio (significant only in the 500 mg/kg group).

The 3-month-old adult zebrafish were used for the evaluation of PPP as an anticonvulsant drug (Pisera-Fuster et al., 2017) using a PTZ-induced seizure model of adult zebrafish. After 4 days of exposure (9 μg/L), zebrafish (9 μg/L) were able to modify the seizure threshold by increasing the latency to spasms induced by PTZ without changing the duration of spasms, which suggests that PPP played a significant role in regulating the neurobehavior of zebrafish at adult stages.

Taken together, the systematic review on PPP was restricted to 21 articles and 1 abstract, of which 14 (66.66%) were assembled from zebrafish (Table 2). Among the other five fish species, common carp, fathead minnows, and Nile tilapia are represented by one article each. Rainbow trout is reviewed in two articles, and Japanese medaka was reviewed in three articles. In zebrafish, the effects of PPP were studied during embryo-larval development, with a focus on toxicity, ED effects, and neurobehavioral disorders, whereas the studies in larvae were restricted only to VTG and sex ratio; in adults, PPP was evaluated as an anticonvulsant substance. Among other fish species, in Japanese medaka, the embryos and larvae were used for toxicological assessments, whereas adults were evaluated for estrogen-dependent effects. In fathead minnows, the studies were conducted on larvae focusing on lethal concentrations, and in rainbow trout, juveniles were used for VTG studies; in Nile Tilapia, adults were used for toxicological endpoints, emphasizing oxidative stress and LPO. Despite similarities among various species on toxicological endpoints, the estrogenic effects (ED effects) of PPP in zebrafish larvae observed by Mikula et al. (2006a, b, 2009) were not in agreement with other fish species (Japanese medaka, rainbow trout, and zebrafish). Despite the differences observed, the systematic review indicates that PPP could be detrimental to fish when present in the aquatic environment.

### 3.4 BTP

Like other parabens, BTP has been used as a preservative in beverages, cosmetics, and pharmaceuticals (Guo and Kannan, 2013; Nowak et al., 2018). The antibacterial effects of BTP are mediated by disabling the transmembrane function of fungi and bacteria and inhibiting the production of ATPases and phosphotransferases in the mitochondria (Freese et al., 1973). The USFDA, in 2017, listed BTP as an additive that could be used directly in food (Hubbard et al., 2020). Maternal exposure to BTP during pregnancy significantly increases the risk of atopic dermatitis in children and is negatively correlated with head circumference, weight, and length of the newborns (Geer et al., 2017; Hajizadeh et al., 2021; Thurmann et al., 2021). Adult exposure to BTP ranges from 0.26 to 17760 mg/day, and infant exposure can reach up to 378 mg/day (Andersen, 2008). Studies have shown that BTP is detectable in human urine in concentrations ranging from 0.2 to 1,240 μg/L (Calfat et al., 2010). BTP has been detected in placental tissues, amniotic fluid, and umbilical cord blood (Jimenez-Diaz et al., 2011; Philippat et al., 2013; Towers et al., 2015). Although humans are exposed to BTP primarily via skin contact and oral ingestion, monitoring studies reported that the detected concentration of BTP was found in natural environments, including indoor dust, surface water, and marine systems (Peng et al., 2008; Xue et al., 2015; Fahimipour et al., 2018). In a river in Brazil, the BTP concentration ranged from 1.3 to 2.4 μg/L (Galinaro et al., 2015) and reached up to 14.8 μg/L in rivers and lakes of India (Kachhawaha et al., 2021).

#### 3.4.1 Common carp

Fertilized eggs of common carp were exposed to BTP (0.1–100,000 μg/L) for 96 h, resulting in 100% mortality at 100,000 μg/L (Table 7; Supplementary Table S1). The limited data (Medkova et al., 2023) are insufficient to conclude the full toxic potential of BTP in common carp. Further investigations on other endpoints (embryo–larval development, mortality, and neurobehavior) are also necessary.

**TABLE 7:** Effects of BTP on fish.

Fish	Author	Toxicological endpoint	Reproductive/endocrine-related endpoint	Neurobehavioral endpoint
Common carp (embryos)	Medkova et al. (2023)	i) All embryos died at 1,000–100,000 µg concentrations of BTP		
Fathead minnows (1 dph larvae)	Dobbins et al. (2009)	i) 48 h LC_50_ was 4.2 mg for BTP and 6.9 mg for i-BTP ii) LOEC for larval growth was 1 mg/L for BTP and 3.5 mg/L for i-BTP. iii) The hazard quotient for BTP was 6.5 × 10^−^ ^5^ and for i-BTP was 1.1 × 10^−^ ^4^		
Japanese medaka (10 dph larvae and 2.5 months adult male)	Yamamoto et al. (2007, 2011)	i) 96 h LC_50_ for 10 dpf larvae for BTP was 4.5 mg/L and for i-BTP was 3.1 mg/L ii) A concentration-dependent increase in the plasma VTG level in male adult fish was observed for both BTP (LOEC = 40 μg/L and i-BTP = 20 μg/L) after 14 days of exposure		
Nile tilapia (adults)	Silva et al. (2018); Liu et al. (2023)	i) The calculated 48 h LC_50_ value in adult male fish was 17.80 mg/L ii) Nonlinear alteration in the LPO (GSH and MDA content) and oxidative stress-related enzymes (CAT, GPx, GR, and SOD) in the gills and liver of adult male fish after 6 and 12 days of exposure iii) After 56 days exposure, the skin became darker with upregulation of *α-MSH* and downregulation of *asip2* iv) The GABA and dopamine content of the brain altered (56 days of exposure) v) Genes responsible for phototransduction pathways (*arr3a* and *arr3b*) were downregulated after 56 days of exposure		
Rainbow trout (immature)	Pedersen et al. (2000); Alslev et al. (2005)	i) Concentration-/dose-dependent enhancement of serum VTG after the administration of BTP by immersion, food, or IP injections ii) Accumulation of BTP in the liver was found to be more than that in the muscle		
Zebrafish (embryo-larval development) (exposure period, 72 h)	Zhu et al. (2023)	i) Induced cardiac apoptosis, endocardial and atrioventricular valve damage, insufficient myocardial energy, impaired calcium (Ca^2+^) homeostasis, and depletion in cardiac-resident macrophages ii) The expression of the endocardial flow response gene (*klf2a*) was downregulated, and natriuretic peptide A and B (*nppa* and *nppb*) genes were upregulated iii) The expression of the neutrophil chemotactic factor (*cxcl8a)* was inhibited, while no effect was observed in the expression of the macrophage chemotactic factor (*ccl2)*		
Zebrafish (embryo-larval development) (exposure period, 96 hpf)	Merola et al. (2020a; 2021); Lite et al. (2022); Li et al., 2023); Medkova et al. (2023)	i) LC_50_ was inversely proportional to the duration of exposure [(LC_50_ after 24 hpf = 10.77 mg/L (55.45 µM); 48 h LC_50_ = 4.208 mg (21.66 µM); 72 hpf LC_50_ = 1.953 mg/L (10.06 µM); 96 h LC_50_ = 1.36 mg/L (7 µM); and 120 hpf LC_50_ = 0.966 mg/L (4.973 µM)] ii) Reduction in blood circulation, blood stasis, pericardial edema, misshaped yolk and deformed notochord, bent tail, and deformed pectoral fins iii) Concentration- and time-dependent delay in hatching iv) SOD, GST, and GPx were significantly affected (reduced) v) AChE enzyme activity in the brain decreased, and NO increased vi) Gene expression analysis indicated upregulation of *hsp70* * * *l* (0.1–100 µg/) and *cyp19a1a* mRNA (0.1 μg/L) vii) Increased thigmotaxis in a concentration-dependent manner viii) No significant difference in the visual startle response and the photic synchronization		
Zebrafish (embryos; exposure period, 120 hpf)	Merola et al. (2022)	i) Reduced survivability, body length, heart rate, and hatching significantly in a concentration-dependent manner ii) The activities of CAT, SOD, and alkaline phosphatase reduced, and the MDA activity elevated significantly iii) The expression of several chondrocyte marker genes, including *sox9a, sox9b*, and *col 2a1a*, was downregulated iv) The T3 and T4 contents of the larvae were reduced v) The genes in the HPT-axis, including *crh*, *trh*, *tshβ*, *nkx2.1*, *hhex*, *ttr*, *dio1*, *dio2*, and *ugt1ab*, were downregulated, while *trα* was upregulated vi) The vtg level was increased significantly in larvae exposed to BTP (0.2–1 mg/L) during development. Moreover, E2 levels significantly increased in larvae; T levels significantly decreased in a concentration-dependent manner		
Transgenic zebrafish embryos [*TG (ins:GFP*)]	Brown et al. (2018)	i) Concentration-dependent increase in GSH content ii) Downregulation of *pdx1* and genes involved in GSH synthesis, while upregulation of GSH-disulfide reductase (*gsr*)	i) In the endocrine pancreas, beta (β) cell area increased and fragmentation of the islet cluster and ectopic expression of β cells	
Zebrafish larvae (4 dpf) (20 dpf; juvenile)	Singh et al. (2024)		i) Exhibited β-cell damage in endocrine pancreas	
Zebrafish (3-month-old adult male fish)	Kim et al. (2022)		i) Cortisol and cortisone levels increased, and allopregnanolone levels decreased	ii) Impaired neurobehavior in photosensitivity and memory in a concentration-dependent manner iii) Phototransduction, tight junctions, and neuroactive ligand receptor activity were significantly affected

#### 3.4.2 Fathead minnows

The effect of BTP (n-BTP) and i-BTP on fathead minnows was investigated on 1 dph larvae (Table 7; Supplementary Table S1). The 48 h LC_50_ value was 4.2 mg/L for BTP and 6.9 mg/L for i-BTP (Table 3). The LOEC and hazard quotient for larval growth were 1.0 mg/L and 6.5 × 10^−5^ for BTP and i-BTP, respectively (Table 7); the LOEC for larval growth was 3.5 mg/L, and the hazard quotient was 1.1 × 10^−4^ for i-BTP. Although the study (Dobbins et al., 2009) indicated that BTP was a more potent toxicological compound than i-BTP, other studies on embryo–larval development and in adult fish targeting ED or neurobehavior are yet to be investigated.

#### 3.4.3 Japanese medaka

Japanese medaka larvae (10 dph) were exposed to BTP (n-BTP) and i-BTP, and the 96 h LC_50_ value was 4.5 mg/L for BTP and 3.1 mg/L for i-BTP (Table 7; Supplementary Table S1). The adult male fish (2.5 months) were exposed to BTP (8–1,000 μg/L) and i-BTP (4–500 μg/L) for 14 days. A concentration-dependent enhancement was observed in the serum VTG level of Japanese medaka with an apparent no observed effect concentration (NOEC) value of 40 μg/L for BTP and 20 μg/L for i-BTP (Yamamoto et al., 2007; 2011). Although both BTP and i-BTP were able to induce serum VTG in Japanese medaka and i-BTP was more potent than BTP, studies related to neurobehavioral endpoints are yet to be investigated.

#### 3.4.4 Nile tilapia

Adult Nile tilapia male fish were exposed to BTP (2.7–21.5 mg/L) for 48 h, and the LC_50_ value was 17.80 mg/L (Table-3). Furthermore, adult male Nile tilapia were exposed to BTP (4 mg/L) for 6 and 12 days, and the lipid peroxidation (GSH and MDA content) and the activities of SOD, CAT, GPx, and GR were determined in the gill and liver tissues (Table 7; Supplementary Table S1). It was observed that GSH content increased in the gills only in 6-day fish, while in the liver, GSH content decreased after 6 days and increased after 12 days (Table 7). The MDA content in the gills remained unaltered, while in the liver, a significant decrease was observed after 12 days of exposure. CAT activity remained unaltered in both the liver and gills of fish after 6 and 12 days. SOD increased in the gills after 12 days and in the liver only after 6 days. GPx activity remained unaltered in the gills after 6 and 12 days, while in the liver, it increased after 12 days of exposure (Supplementary Table S1). GR activity remained unaltered in both the gills and liver either after 6 or 12 days (Silva et al., 2018).

Nile tilapia adults exposed to BTP (5–5,000 ng/L) for 56 days induced darker skin pigmentation, probably by increasing the melanin content of the skin (Table 7; Supplementary Table S1). Gene expression analysis showed that genes related to melanin synthesis, such as *α-MSH* (upregulated) and *asip2* (downregulated), altered significantly, and the level of dopamine and γ-aminobutyric acid (GABA) content in the brain reduced. Moreover, the genes related to the phototransduction pathway (*arr3a* and *arr3b*) were upregulated by BTP in a concentration-dependent manner, indicating interference of phototransduction from the retina to the brain of the fish (Liu et al., 2023).

#### 3.4.5 Rainbow trout

Juvenile rainbow trout injected with BTP (50–200 mg/L) significantly increased plasma VTG levels in a dose-dependent manner (Pedersen et al., 2000). The estrogenic effect of BTP was investigated in sexually immature rainbow trout exposed either orally (4–74 mg/kg/2d for 10 days) or by immersion (35 and 201 μg/L for 12 days). It was observed that the plasma VTG level was induced in fish in a time- and concentration-dependent manner (Alslev et al., 2005). BTP showed little tendency to bioaccumulate in the body of the rainbow trout (less than 1% was retained in the liver at the end of the experiment) (Table-7; Supplementary Table S1). A positive correlation was observed between the concentration of VTG and BTP in the plasma of the experimental fish (Alslev et al., 2005).

#### 3.4.6 Zebrafish

The toxic potential of BTP was observed in zebrafish during embryo–larval development and in adults. The embryos were exposed to a wide range of BTP (0.1–100,000 μg/L), and the toxic effects (developmental, endocrinological, and neurobehavioral endpoints) were evaluated at various time points (72 hpf–6 dpf). The larvae (96 hpf) were used to evaluate the damage induced by BTP (2,500 μg/L) on pancreatic β-cells of zebrafish (Singh et al., 2024). The adult male zebrafish were exposed to BTP (10–1,000 μg/L) for 28 days, and the neurobehavioral disorders of the fish were evaluated (Kim et al., 2022). The calculated LC_50_ value of BTP was inversely related to the duration of BTP exposure, with LC_50_ concentrations (LC_50_) decreasing as the duration of exposure increased (Li et al., 2023). Moreover, 100% mortality was observed in embryos exposed to 1,000–100,000 μg/L concentrations (Table-7; Supplementary Table S1).

Embryos were exposed to sublethal concentrations of BTP (0.6–1.8 mg/L) for 72 hpf, and morphological defects and dysfunctions of the heart were evaluated (Zhu et al., 2023). It was observed that BTP induced systolic heart failure through a multifactorial effect, including cardiac apoptosis, endocardial and atrioventricular valve damage, insufficient myocardial energy, impaired calcium (Ca^2+^) homeostasis, depletion in cardiac-resident macrophages, and oxidative stress (Table 7; Supplementary Table S1). Moreover, the expression of the endocardial flow response gene (*klf2a*) was downregulated, and natriuretic peptide A and B (*nppa* and *nppb*) genes were upregulated after BTP (1.8 mg/L) exposure (Zhu et al., 2023). The pro-inflammatory cytokine genes, *tnfα* and *il1β*, were upregulated after BTP exposure, while there was no recruitment of neutrophils in the cardiac region (Zhu et al., 2023). The gene expression analysis indicated that the expression of neutrophil chemotactic factor *cxcl8a* was inhibited, while the macrophage chemotactic factor *ccl2* showed no significant effect (Zhu et al., 2023).

Embryos exposed to BTP (0.1–100,000 μg/L) for an additional day (96 h) induced concentration-dependent mortality. Developmental abnormalities (reduction in blood circulation, blood stasis, pericardial edema, misshaped yolk, bent tail, deformed notochord and pectoral fins, and hatching delay) were induced by BTP in embryos in a time- and concentration-dependent manner (Table-7; Supplementary Table S1). The LPO enzymes (SOD, GST, and GPx) were significantly affected (reduced) by BTP exposure (Lite et al., 2022). The AChE activity in the brain was decreased by BPT, and NO was increased (Lite et al., 2022). Gene expression analysis indicated the upregulation of *hsp70 l* (0.1–100 µg/) and *cyp19a1a* mRNA (0.1 μg/L) (Medkova et al., 2023). The LPO and enhancement of apoptotic cells in the brain indicate that BTP interrupts the defense system of zebrafish. The behavioral tests (thigmotaxis, visual startle response, and photic synchronization) after exposing the zebrafish embryos to BTP (5–500 μg/L, 96 hpf) have been done on 4–6 dpf larvae (Merola et al., 2021). BTP exposure increased thigmotaxis in a concentration-dependent manner; however, no significant difference was observed in the visual startle response and photic synchronization.

The toxic potential in zebrafish was observed after exposing the embryos to BTP (0.1–16 mg/L) for 120 hpf and evaluating the deformities in craniofacial cartilages during development (Table 7; Supplementary Table S1). In addition, mortality, body length, periocular edema, cardiac dysplasia, delayed otolith development, tail curvature in larvae, oxidative enzymes (CAT, SOD, and MDA), hormone concentrations (T3, T4, E2, T, cortisol, and ACTH), and VTG content were evaluated (Liang et al., 2022; 2023a; 2023b; Li et al., 2023). As expected, BTP reduced survivability, body length, heart rate, and hatching significantly in a concentration-dependent manner. Moreover, the activities of CAT and SOD reduced, and MDA elevated significantly. The activity of alkaline phosphatase (responsible for osteoblast activity) was reduced (Table 7; Supplementary Table S1). Moreover, the expression of chondrocyte marker genes, including *sox9a*, *sox9b*, and *col 2a1a*, was downregulated (Li et al., 2023). The T3 and T4 contents of the larvae (120 hpf) were also reduced significantly by BTP (1–2 mg/L). The HPT-axis genes, including *crh*, *trh*, *tshβ*, *nkx2.1*, *hhex*, *ttr*, *dio1*, *dio2*, and *ugt1ab*, were downregulated, while *trα* was upregulated by BTP (2 mg/L) (Liang et al., 2022). The VTG level was increased significantly in larvae exposed to BTP (0.2–1 mg/L) during development. Moreover, the E2 level was also significantly increased in larvae exposed to BTP (0.2–0.4 mg/L), while T levels significantly decreased in a concentration-dependent manner (Liang et al., 2023a). AChE activity, ACTH, and cortisol levels were altered after BTP exposure (Liang et al., 2023b). The gene expression analysis indicated downregulation of *mr*, *crhbp*, and *crhr2*, while the expression of *gr* did not show any significant alterations. The swimming behavior (total distance covered by swimming and the swimming velocity) of the zebrafish larvae (120 hpf) was reduced after BTP (5 µM).

Transgenic zebrafish embryos [*TG (ins: GFP*)] exposed to BTP (48.5–583 μg/L; 250–3,000 nM) from 3 hpf until 7 dpf caused intestinal effusion, pericardial edema, and accelerated yolk utilization (Brown et al., 2018). Beta (β) cell area increased (250 nM), and fragmentation of the islet cluster and ectopic expression of β cells were observed (Supplementary Table S1). GSH content was increased in a concentration-dependent manner. Moreover, downregulation of *pdx1* and upregulation of GSH-disulfide reductase (*gsr*) were observed (Table 7; Supplementary Table S1). Other than embryos, larvae of zebrafish (96 hpf) exposed to BTP (2.5 mg/L) for 5 days exhibited β-cell damage; however, pretreatment with morin effectively reduced mortality and mitigated apoptosis and lipid peroxidation, thus protecting the β-cells from BTP-induced damage (Singh et al., 2024).

BTP was able to cross the blood–brain barrier in adult zebrafish exposed to BTP (0.01–1 mg/L) for 28 days and impaired neurobehavior and memory in a concentration-dependent manner (Table 7; Supplementary Table S1). RNA-seq analysis showed that phototransduction, tight junctions, and neuroactive ligand receptor activity were significantly affected. Among the neurosteroids, cortisol and cortisone levels were increased, and allopregnanolone levels were decreased in all tested concentrations (Kim et al., 2022).

Taken together, our systematic review on BTP was restricted to only 18 articles, of which 11 (61.11%) were assembled from studies with zebrafish (Table 2). Among the other five fish species, common carp and fathead minnows were represented by one article each. Nile tilapia, rainbow trout, and Japanese medaka were reviewed in two articles each. In zebrafish, the effects of BTP were studied during embryo–larval development, with an emphasis on toxicity (mortality and lethal effects), ED effects (HPG-, HPI-, and HPT-axis), and neurobehavioral disorders (movement disorders and AChE activity), whereas the studies in larvae were restricted only to pancreatic beta cells; in adults, BTP was evaluated as a neurobehavioral disruptor. Among other fish species, in Japanese medaka, the embryos and larvae were used for toxicological assessments, whereas adults were evaluated for estrogen-dependent effects. In fathead minnows, the studies were done on larvae, focusing on lethal concentrations, and in rainbow trout, juveniles were examined for VTG activity; Nile tilapia adults were focused on oxidative stress and lipid peroxidation. Despite the similarities among various species regarding toxicological, endocrinological, and behavioral endpoints, the review indicates that BTP, like other parabens, is detrimental to fish.

### 3.5 BNP

Although BNP is not frequently used, it is recognized as the most potent paraben in terms of estrogenic activity and was found to interfere with male reproductive functions (Routledge et al., 1998; Oishi, 2001). During the literature search, we did not focus our aim on BNP; however, few investigators have examined the effects of BNP on fish. We, therefore, briefly describe the effects of BNP in various fish species (fathead minnows, Japanese medaka, and Nile tilapia) found during the search process.

#### 3.5.1 Fathead minnows

Fathead minnows (1 dph larvae) were exposed to BNP, and the 48 h LC_50_ value was 3.3 mg/L (14.45 µM) (Table 3). The LOEC was 1.7 mg/L, and the hazard quotient was 2.1 × 10^−4^. No other information is available (Dobbins et al., 2009).

#### 3.5.2 Japanese medaka

Japanese medaka larvae (10 dph) were exposed to BNP, and the 96 h LC_50_ value was 0.73 mg/L (3.2 µM) (Table −3). Adult male medaka (2.5 months old) exposed to BNP (4–500 μg/L) for 14 days enhanced plasma VTG in a concentration-dependent manner (Supplementary Table S1). The NOEC was 20 μg/L (Yamamoto et al., 2007; 2011). DNA microarray analysis showed upregulation of *vtg1*, *vtg2*, *chgL*, *chgH*, and *esr1* in the liver of male fish in a concentration-dependent manner (6 genes in fish exposed to 4 μg/L and 41 genes in fish exposed to 500 μg/L); however, several genes were downregulated in a nonlinear fashion (Yamamoto et al., 2007). No other information is currently available.

#### 3.5.3 Nile tilapia

Adult Nile tilapia was exposed to BNP (2.1–16.9 mg/L) for 48 h. The 48 h LC_50_ value was 7.98 mg/L (34.96 µM) (Table 3). Furthermore, adult male Nile tilapia were exposed to BNP (4 mg/L) for 6 and 12 days, and the LPO (GSH and MDA content) and oxidative stress-related enzymes (SOD, CAT, GPx, and GR) in the gill and liver were assayed (Supplementary Table S1). It was observed that GSH remained unaltered in the gills and increased in the liver after 12 days of exposure. Moreover, MDA content remained unaltered in both the liver and gills after 6 and 12 days (Silva et al., 2018); CAT and GR activities remained unaltered in both the liver and gills; however, SOD increased in the gills after 6 and 12 days, and no alteration was observed in the liver. GPx showed enhanced activities in both the gill and liver of fish exposed to BNP (4 mg/L) for 12 days (Silva et al., 2018).

From these studies, we can summarize that among three species, Japanese medaka larvae were more sensitive than fathead minnow larvae (LC_50_ 3.2 µM vs 14.45 µM) although the duration of exposure (96 h vs. 48 h) and the age of the larvae (10 dpf vs. 1 dpf) were different. BNP showed ED effects in adult male medaka, probably mediated through oxidative stress.

## 4 Discussion

In the past few years, due to methodological and technological advancements, many emerging pollutants have been detected in aquatic ecosystems. Even at low concentrations, different environmental monitoring studies have reported the presence of pharmaceuticals and PCPs, including parabens, in some ecosystems at levels that can potentially lead to negative impacts on aquatic organisms (Golovko et al., 2021; Vale et al., 2022). Their occurrence in the environment raises concerns not only about human health but also about wildlife (Vale et al., 2022). Despite the state of knowledge, most emerging compounds are still poorly characterized in terms of their fate, behavior, toxicity, and impact on nontarget organisms.

Parabens are alkyl esters (group of *para*-hydroxybenzoic acid) (Figure 1), introduced in the mid-1920s (Liebert, 1984). They are now widely used as preservatives in foodstuffs, cosmetics, toiletries, PCPs, and pharmaceuticals and pose considerable exposure risks to the environment (Bledzka et al., 2014). In terms of chemical structure, they can mainly be classified as MTP, ETP, PPP, and BTP with prolonged alkyl substituents (Figure 1) (Bledzka et al., 2014). The alkyl chain length of paraben esters can positively correlate with their antimicrobial property (Doron et al., 2001; Gao et al., 2016). Parabens can be absorbed through intact skin and hydrolyzed by carboxylesterases in subcutaneous fatty tissues (Boberg et al., 2010). Studies showed that parabens are rapidly absorbed from the gastrointestinal tract and blood, hydrolyzed to *p*-hydroxybenzoic acid, conjugated, and then excreted in the urine, but some elimination can also occur through the bile and feces (Boberg et al., 2010; Vale et al., 2022).

The current literature survey reviewed the toxicological effects associated with the exposure of fish to parabens, focusing on development (embryos, larvae, and adults), oxidative stress (LPO and oxidative enzymes), EDs (HPG-, HPI-, and HPT-axis), and neurobehavioral disorders (AChE enzyme and movements). In this article, we have reviewed only 48 articles (47 full papers and 1 abstract), emphasizing the effects of 4 commonly used parabens (MTP, ETP, PPP, and BTP) on 6 fish species (common carp, fathead minnows, Japanese medaka, Nile tilapia, rainbow trout, and zebrafish) (Figure 2; Tables 1, 2). Among these articles, only one article (Medkova et al., 2023) included two fish models (common carp and zebrafish) as test species, while the remaining articles focused on only one fish model during investigations (Tables 1, 2). Moreover, study results on common carp were described in only one article, fathead minnows in one article, Japanese medaka in four articles, Nile tilapia in two articles, rainbow trout in three articles, and zebrafish in thirty-five (also one abstract) articles (Figure 2; Table 2). Furthermore, studies on MTP in rainbow trout primary hepatocytes cultures (Grung et al., 2007) and the effects of PPP as a component of effluents in zebrafish (Golovko et al., 2021) were included in this review. Moreover, studies were mostly focused on embryo-larval development (common carp, Japanese medaka, and zebrafish), larvae (fathead minnows and Japanese medaka), and adults (zebrafish, Japanese medaka, and Nile Tilapia). Among parabens, the effects of MTP were studied in 24 articles, ETP in 11, PPP in 24, and BTP in 21 articles (Tables 1, 2). Therefore, the articles reviewed in this review are mostly based on embryo-larval development of zebrafish. With the exception of ETP (11 articles), for other three parabens (MTP, PPP, BTP) more than 20 peer reviewed articles were included in these fish models (Figure 2; Table 2).

Our literature search on the lethal effects (LC_50_, NOEC, LOEC, and BMD) of parabens in fish was limited to only four fish species: zebrafish (embryos, larvae, and adults), fathead minnows (larvae), Japanese medaka (larvae), and Nile tilapia (adults) (Table 3); none of the studies reviewed the LC_50_ values of parabens in common carp and rainbow trout. Moreover, the calculated LC_50_ value of parabens for fish varies depending on species, developmental stage of the fish, chemical structure, and the duration of exposure of the fish to parabens (Table 3). The LC_50_ values were negatively correlated with the carbon chain length of the alkyl group (as the carbon chain length increases, the LC_50_ values decrease), and the toxicity order followed was BTP > PPP > ETP > MTP (Liang et al., 2022). Similar findings on paraben toxicity were observed in *Caenorhabditis elegans* (a nematode), *Daphnia magna* (an aquatic Cladocera), *Tigriopus japonicus* (a marine copepod), and unicellular green algae (*P. subcapitana*) (Terasaki et al., 2009a; Dobbins et al., 2009; Yamamoto et al., 2011; Lee et al., 2018; Kang et al., 2019; Nagar et al., 2020). Moreover, longer exposure (120 hpf) effects are observed at lower concentrations than at shorter duration (96 hpf). For example, in zebrafish embryos, the 24 hpf LC_50_ value of BTP was 10.77 mg/L, while the 120 hpf LC_50_ value was 0.966 mg/L (Li et al., 2023). Furthermore, the branching of the alkyl chains (*n*-PPP vs *i*-PPP; *n*-BTP vs *i*-BTP) has a significant impact on paraben toxicity (Obringer et al., 2021); however, our review found that it is not consistent within the fish species (Dobbins et al., 2009; Yamamoto et al., 2011). Moreover, in adult zebrafish, the 96 h LC_50_ value of MTP was 150.09 mg/L (de Carvalho Penha et al., 2021), which is significantly higher than the values (1.102 mg/L) reported by Thakkar et al. (2022). Therefore, the toxicity based on the carbon chain length of the alkyl group in parabens could be attributed to the lipophilicity of the test compound, which was directly related to the bioavailability and bioaccumulation potential of the organic pollutants (Bekele et al., 2019; Gao et al., 2016; Liang et al., 2022).

Our literature search highlighted the detrimental effects of parabens on the embryos (common carp, Japanese medaka, and zebrafish), larvae (Japanese medaka and zebrafish), juveniles (rainbow trout), and adult fish (Japanese medaka, Nile tilapia, and zebrafish) (Tables 3–7). Despite LC_50_ values (Table 3), the endpoints on toxicity were assessed on the yolk sac, heart, blood, tail, eye, pigmentation, hatching, and growth during embryo–larval development, as well as on growth (length and weight), and histological and biochemical parameters of the liver, gills, intestine, gonads, and brain in adult fish (Tables 4–7). It was observed that the early developmental stages of zebrafish are more sensitive and susceptible to the effects of xenobiotic exposure compared to the larval and adult stages (Zon and Peterson, 2005; He et al., 2014; Lantz-Mcpeak et al., 2015; Lite et al., 2022). Yolk sac edema is considered a sensitive toxicological endpoint for embryonic exposure studies. Although this phenotypic feature (yolk sac edema) is not observed during human embryonic development, clinical investigations have reported an increased incidence of yolk sac edema observed in pregnancies, leading to spontaneous abortion (Nogales et al., 1992; Sant and Timme-Laragy, 2018). The yolk sac functions as the main site from where the lipids (nutrient source) are transported to embryos and larvae, ensuring growth and survival. Zebrafish embryos exposed to sublethal concentrations of PPP for 96 h induced neurocranial defects, probably by decreasing neutral lipid mobilization from the yolk and impairments of phospholipid metabolism both in the body and in the yolk sac (Perugini et al., 2020). In Japanese medaka embryos exposed to sublethal concentrations of PPP, lesions included myoskeletal and cardiovascular damage, an enlarged peritoneal cavity, hepatic atrophy with a concomitant increase in gall bladder size, collapse of the airbladder, and disrupted renal histology; however, EROD activity (P4501A) did not reveal alteration (Gonzalez-Doncel et al., 2014). Thus, the paraben-induced yolk sac malformation will affect lipid metabolism, subsequently leading to developmental delay in exposed embryos and defects in the musculoskeletal system (Andersen, 2011; Gonzalez-Doncel et al., 2014; Zoupa and Machera, 2017; Li et al., 2023).

Pericardial edema is another physiological marker that reflects the functioning of the heart and its associated toxicity. Zebrafish embryos exposed to parabens induced abnormal cardiac functions and morphology by disrupting the expression of genes related to retinoic acid metabolic pathways, myocardial contractility, cardiac cell apoptosis, and heart tube development (Bereketoglu and Pradhan, 2019; Merola et al., 2020a; Fan et al., 2022). In addition, there are reports that the insufficient synthesis of cardiac troponin T contributes to pericardial edema in zebrafish embryos (Chen, 2013). The early embryonic exposure to polycyclic aromatic hydrocarbon mixtures affected cardiac conduction, which led to secondary consequences like pericardial edema and disrupted cardiac morphogenesis (Incardona et al., 2004). Embryos exposed to psychoactive substances such as caffeine and arecoline have induced bradycardia in zebrafish (Rana et al., 2010; Peng et al., 2015).

Hatching is a key point in the life cycle of fish. Hatching of the embryos occurs due to a combination of biochemical and physiological mechanisms. Enzymes including Zn-metalloproteases (high and low choriolytic enzymes) act on the chorion, followed by physical movement of twitching of the embryo, which contributes to the breaking of the chorion (De Gasper et al., 1999). Exposure of the embryos to parabens resulted in a decreased hatching rate with increasing concentrations, which may be due to the decrease in spontaneous movements of the embryos. The disruption of redox balance during early development has been studied to result in malformations and compromised growth parameters in fish (Newman et al., 2015). Paraben (PPP and BTP) exposure increased oxidative stress with suppressed antioxidant enzyme activity; thus, the overall collapse in the redox homeostasis and cellular apoptosis could be the reason for the developmental malformation observed in the zebrafish embryo–larval development (Lite et al., 2022; Li et al., 2023).

The toxicological effects of parabens suggest the potential hazardous risks incurred by intended or unintended exposure (Liang et al., 2022). The TEI values during embryonic development suggest higher teratogenicity rather than embryotoxicity (Tran et al., 2023). Moreover, developmental exposure of zebrafish and common carp embryos to parabens leads to disturbance in oncogene transcription (*myca* and *ccnd1*), which is associated with oxidative stress, DNA double strand breaks, apoptosis, and fatty acid metabolism (Dambal et al., 2017; Ates et al., 2018; Raja et al., 2019; Bereketoglu and Pradhan, 2019; Lite et al., 2022; Medkova et al., 2023). In adult zebrafish, sublethal MTP exposure increased the incidence of hepatic histopathology (swollen and vacuolated hepatocytes), which was sex-specific, with the condition being more severe in female fish than in male fish (Hu et al., 2022a). Exposure of male fish to MTP showed a significant reduction in the EROD activity in the gills and an increase in LPO (de Carvalho Penha et al., 2021). Lipid metabolism along the gut–liver axis was also remarkably disturbed, targeting nuclear receptor signaling and concentrations of key metabolites, primarily associated with fatty acyls, retinoids, and steroids (Hu et al., 2022a). Major concerns of MTP-induced hepatotoxicity were associated with cortisol-mediated stress response, blockage of primary bile acid synthesis, and enhanced degradation of bioactive molecules like retinoic acid and estradiol (Hu et al., 2022a). Moreover, MTP exposure, especially in female fish, induced dysbiosis in gut microbiota (Hu et al., 2022b). Although the length and weight of the female zebrafish increased significantly after 28 days of exposure to MTP (Hu et al., 2022b), the number of goblet cells in the intestine decreased in female fish and increased in male fish (Hu et al., 2022b). Consequently, the expression of TJP2 was upregulated in male fish and downregulated in female fish. The serotonin content in the gut is increased in male fish and decreased in female fish. Under stress, CAT was activated to scavenge free radicals in the intestine and reduce oxidative stress (Hu et al., 2022b). In Nile tilapia adults, male fish exposed to parabens induced alterations in the antioxidant enzyme activities and nonenzymatic antioxidant contents in the gill and liver of the fish, indicating an antioxidant adaptive response for the neutralization of ROS-generated oxidative stress (Silva et al., 2018). Zebrafish adults, exposed to MTP (1.1–111 μg/L) for 30 days, showed concentration-dependent downregulation in genes related to cardiac hypoxia and neuronal differentiation in female fish, while in male fish, the downregulation of these genes (nonlinear) occurred (Thakkar et al., 2022). Taken together, the toxic response of the parabens in fish, especially during embryo–larval development, is mediated by oxidative stress and LPO, cell cycle and DNA damage, inflammation, and fatty acid metabolism (Silva et al., 2018; Hu et al., 2022a; Hu et al., 2022b).

Since the early developmental stage is a highly regulated event, perturbation at the endocrine level can affect the transition from early stages to adulthood (Dambal et al., 2017; Spaan et al., 2019). Our review focused on the response of HPT-, HPG-, and HPI-axis of the fish to parabens (Tables 4–7; Supplementary Table S1). The decrease in T3 and T4 levels could cause a delay in the growth of the fish embryos, hatching rates, heartbeats, and the induction of malformations (Liang et al., 2022). Among the genes responsible for TH effects, *tg*, *tpo*, and *nis* are involved in TH synthesis, *nkx2.1*, *pax8*, and *hhex* regulate the development of the thyroid gland, and *ttr* is responsible for the transport of THs to target tissues (Wang et al., 2013a; Liu et al., 2019). *dio1* and *dio2* mediate the peripheral and circulating TH contents in fish by recovering iodine and removing the hormones or catalyzing T4 into T3 (Yang et al., 2019). Moreover, *ugt1ab* regulates the inactivation and excretion process of exogenous and endogenous compounds, and the expressions of THRs (*trα; trβ*) can determine the regulatory function of THs on the HPT-axis (Parsons et al., 2020). Molecular docking analysis testified that MTP, ETP, PPP, and BTP exhibited thyroid receptor agonistic activities (Liang et al., 2022). The expressions of many of the HPT-axis genes were decreased in zebrafish larvae after MTP, ETP, and BTP exposure (Liang et al., 2022), suggesting a significant regulatory role played by parabens in the HPT-axis. However, PPP increased the expression of *trα* and *trβ* and stimulated the expression of HPT-axis genes, which were different from the disruptions caused by other parabens. Despite the differences, disruption of TH signaling (Koeppe et al., 2013) modulated by PPP could contribute to the observed developmental malformations during embryo–larval development of fish (Bereketoglu and Pradhan, 2019; Kang et al., 2019; Liang et al., 2022).

The four common parabens also regulate the HPG-axis by disrupting steroidogenesis in fish. Studies showed that parabens are ER agonists (Terasaki et al., 2009b; Watanabe et al., 2013) and AR antagonists (Ding et al., 2017). Induction of VTG in male fish is a well-established endpoint for identifying the estrogenic activity of chemicals (Sumpter and Joblings, 1995; Chow et al., 2012; Wang et al., 2013b), and parabens showed the potential to induce VTG in fish (Tables 4–7; Supplementary Table S1). Our literature search found that only three fish species (Japanese medaka, rainbow trout, and zebrafish) were investigated for VTG induction by parabens (Tables 4–7). VTG is normally produced in the liver of adult female oviparous vertebrates, serving as a yolk protein precursor, and remains dormant in juvenile or male animals (Brion et al., 2002). When exposed to estrogen mimics, e.g., ethinyl estradiol (EE2), *vtg* and other genes are activated and show an increasing VTG expression (Muncke and Eggen, 2006; Soares et al., 2012). In zebrafish larvae, VTG was significantly increased after paraben exposure (Dambal et al., 2017; Liang et al., 2023a); however, zebrafish larvae (20 dph) exposed to PPP either by food (500–2000 mg/kg) or immersion (0.1–0.9 mg/L) for 20 days were unable to induce VTG content (whole body) (Mikula et al., 2006a; b; 2009). On the other hand, the sexually immature rainbow trout enhanced the VTG level in plasma when exposed to ETP, PPP, or BTP (Tables 5–7). Histopathological evaluation of adult zebrafish gonads after exposure to sublethal concentrations of MTP showed degenerative effects on testis histology (Hassanzadeh, 2017) and thus blocked gametogenesis, decreased E2, T, and 11-KT levels in the blood, and suppressed the hepatic VTG production (Hu et al., 2023b). The gene expression in the HPG-axis of zebrafish larvae (120 hpf) exposed to sublethal concentrations of MTP, ETP, and BTP disrupted the expression of many genes and their receptors (Liang et al., 2023a), indicating that parabens have the potential to modulate the endocrine functions during the embryo–larval development of zebrafish (Bereketoglu and Pradhan, 2019; Liang et al., 2023a). Zebrafish embryos exposed to 100 µM MTP induced *vtg1* mRNA in the whole body of the larvae on 96 hpf (Dambal et al., 2017). In adult zebrafish, MTP significantly interrupted the gene transcriptions involved in the feedback loop, steroidogenesis, and nuclear receptor signaling along the entire HPG-axis and liver (Hu et al., 2023b). In adult Japanese medaka male fish, exposed to sublethal concentrations of MTP and PPP, the expression of *vtg1*, *vtg2*, *chgL*, *chgH*, *esr1*, and *esr2* mRNAs was enhanced in a concentration-dependent manner (Inui et al., 2003; Yamamoto et al., 2011). Consequently, the level of plasma VTG in male fish was also enhanced by MTP and PPP, and the expression of *ar* mRNA remained unaltered (Supplementary Table S1). The binding affinities of parabens (PPP and BTP) to *esr1* and *esr2* were analyzed by molecular docking analysis. The results indicated considerable binding activities of these two parabens to the estrogen receptor isoforms, which were negatively correlated with the complexity of the chemical structure (Liang et al., 2023a). After considering transcriptional, hormonal, and histopathological data, MTP and other parabens inhibited the signaling transduction of FSH from the pituitary to the testis, which played a pivotal role in E2 production (Clelland and Peng, 2009). Accordingly, a series of enzymatic reactions that catalyzed steroidogenesis were disrupted by MTP (parabens), reduced the circulating E2 level in zebrafish (Liang et al., 2023a), and induced disorders in the HPG-axis. The variation in ED responses during embryo-larval development compared to adulthood underlines distinct modes of action, probably because the HPG-Axis in adult fish is fully functional and more active than the fish in embryo–larval stages.

Our review also observed that parental exposure to MTP induced developmental deficits in offspring (larvae) by increasing mortality, stimulating precocious hatching, and elevating heart rates although the malformation rates (yolk edema, pericardial edema, and bent spine) and the growth (length and weight) during embryo–larval development did not establish a significant difference (Hu et al., 2023b). This aspect is very important because the effects of MTP (parabens) can be intergenerationally transmitted to embryos/larvae from parental generation (P1) to the offspring and can disrupt embryo–larval development in zebrafish (Hu et al., 2023b). Although the study was restricted only to MTP, which has the lowest log *K*_*ow*_ value and the shortest alkyl side chain length compared to other parabens, this may have a significant impact on the bioaccumulation of the pollutant. To further understand this aspect, an in-depth investigation using other parabens (ETP, PPP, and BTP), which have higher log *K*_*ow*_ values and longer alkyl side chain lengths than MTP, is required. In addition, using other fish models or other test methods, like the fish full life cycle test (FFLC), is necessary.

The four parabens were used to evaluate the neuroendocrine system of the zebrafish larvae by investigating the behavior, ACTH and cortisol levels, AChE activity, and gene expressions in the HPI-axis (Liang et al., 2023b). Our literature search indicates that among six fish species, only the larvae (Merola et al., 2021; 2024; Liang et al., 2023b; Tran et al., 2023) and adults (Kim et al., 2022; Thakkar et al., 2022) of zebrafish were used for neurobehavioral tests. It was observed that all the tested parabens significantly reduced the swimming behavior; however, they did not influence the visual startle response or the photic synchronization of circadian rhythms (Merola et al., 2024). MTP exposure in embryos inhibited the AChE activity and induced anxiety-like behavior in larvae as a consequence of an increase in cortisol concentration (Raja et al., 2019). Moreover, ACTH levels decreased, while cortisol levels were increased by parabens (Liang et al., 2023b). Gene expression analysis indicated downregulation of the target genes, including *gr, mr*, and *crhr2*, in the HPI-axis. Parabens were able to bind to the glucocorticoid receptor and trigger its transactivation (Liang et al., 2023b). BTP was able to cross the blood–brain barrier in adult male zebrafish and impaired neurobehavior and photosensitivity. In Nile tilapia adults, BTP induced darker skin pigmentation by the neuroendocrine circuit, probably by inhibiting phototransduction from the retina to the brain (Liu et al., 2023). Therefore, alterations in the neurosteroid levels (cortisol and cortisone increased and allopregnanolone decreased) and neurotransmitters (histaminergic, cholinergic, dopaminergic, serotonergic, and GABAergic) affected phototransduction and abnormal responses to light stimulation, learning, and memory (Kim et al., 2022).

The AChE activity was used as a biomarker for the effects induced by neurotoxic compounds (Tao et al., 2022). AChE activity in zebrafish larvae showed concentration-dependent significant induction in MTP and ETP exposures. However, no obvious changes were observed in the PPP- and BTP-exposed groups (Liang et al., 2023a; Liang et al., 2023b). The elevation of AChE activity might result in the accelerated degradation of the neurotransmitter acetylcholine, thus probably decreasing the cognitive functions of the organism (Tougu and Kesvatera, 1966). The light–dark preference test considers the natural affinity of an animal to avoid or explore certain zones of a given environment, which is correlated with the suppression of the activity of the AChE levels; this suggests a potential involvement of cholinergic mechanisms in the altered neurobehavioral outcomes (Cho et al., 2012) and also highlights its role as a neurotoxic biomarker in aquatic organisms (Lite et al., 2022). Diazepam, an anxiolytic drug, and caffeine and pentylenetetrazole, which are demonstrated to be anxiogenic, have been shown to decrease or enhance thigmotaxis in zebrafish larvae (Schnorr et al., 2012; Lundegaard et al., 2015; Yang et al., 2017). BTP in zebrafish larvae (Lite et al., 2022) and MTP in adults showed more anxiety-like behavior in female fish than in male fish (Thakkar et al., 2022), followed by a concentration-dependent decrease in AChE activity and an increase in serotonin concentration in female fish while male fish exhibited a decrease in serotonin concentration (Thakkar et al., 2022).

Proteomics analysis of the brains in adult male and female fish exposed to MTP (1–10 μg/L, 28 days) revealed significant impacts on the nervous system (Wei et al., 2021). In adult zebrafish, a negative feedback mechanism was activated by the hypothalamus, subsequently blocking signaling cascades along the neuroendocrine axis, which in turn reduced blood cortisol concentrations and increased the infiltration of lipopolysaccharide (LPS) into the male brain (Hu et al., 2023a). Moreover, MTP exposure stimulated the synthesis of pro-inflammatory cytokines in the male brain, while in female fish, it enhanced the expression of BBB proteins, thus inhibiting the penetration of LPS into the brain (Hu et al., 2023a).

Collectively, parabens, as anxiogenic neuroactive compounds, are capable of inducing anxiety-like behavior through a mechanism involving oxidative stress-induced apoptosis. Moreover, transcriptomic analysis revealed that the hyperactivity was associated with disruptions in molecular pathways related to neurotoxic mechanisms, including signaling, transport, calcium ion binding, and metal binding (Tran et al., 2023).

## 5 Conclusion

Our systematic review of the available literature in PubMed provided the basic toxicological data on the effects of parabens on fish while also raising safety concerns regarding the use of parabens. Although the studies were mostly confined to the embryo–larval development of zebrafish, the chemical structure-dependent activities of parabens affecting development, the endocrine system, and neurobehavior provided new insights into their deleterious effects. Due to the differences in the maturity level of the hypothalamus–pituitary axis (HPT- HPG-, and HPI-axis), the responses of fish to parabens observed during embryo–larval development are not exactly identical to those observed in adults. Oxidative stress and lipid peroxidation, cell cycle and DNA damage, inflammation, and fatty acid metabolism played significant roles in paraben toxicity, especially during embryo-larval development. Although the observed effects of parabens in fish are mostly based on concentrations higher than those found in the environment, some of the effects , such as activation of the AR, ER, and TR genes, can be induced even at concentrations below those detected in aquatic environments. Moreover, since the modes of action of the parabens are common, combined exposure to more than one paraben in the environment, even when the individual concentrations are below the LD_50_ limits, can induce toxic effects in fish. Considering the widespread use of parabens in the world, it is essential to standardize their doses in food and cosmetics, especially for specific groups such as pregnant women, and the potential risk of parabens to human health needs to be carefully addressed. The present systematic review broadens our knowledge of the effects of parabens and can be helpful for the development of strategies to explore the molecular mechanisms of the underlying toxicity associated with parabens on human health.

## Figures and Tables

**Table:** 

**AChE**	Acetyl cholinesterase
**ACTH**	Adrenocorticotrophic hormone
**AR**	Androgen receptor
**BBB**	Blood–brain barrier
**BMD**	Benchmark dose
**BNP**	Benzyl paraben
**BTP**	Butyl paraben
**CAT**	Catalase
**CHG**	Choriogenin
**dph**	Days post hatch
**ED**	Endocrine disruption
**ETP**	Ethyl paraben
**E2**	Estrogen
**ERs**	Estrogen receptors
**EED**	Estrogen endocrine disruptors
**EROD**	Ethoxyresorufin O-deethylase
**FDA**	Food and Drug Administration
**GSH**	Glutathione
**GPx**	Glutathione peroxidase
**GST**	Glutathione S-transferase
**HPG axis**	Hypothalamus–pituitary–gonad axis
**HPI axis**	Hypothalamus–pituitary–interrenal gland axis
**hpf**	Hour post-fertilization
**i-BTP**	i-butyl paraben
**11-KT**	11-keto testosterone
**n-BTP**	n-butyl paraben
**LPO**	Lipid peroxidation
**LPS**	Lipopolysaccharide
**MDA**	Malondialdehyde
**MTP**	Methyl paraben
**NO**	Nitric oxide
**PTZ**	Pentylenetetrazole
**PCPs**	Personal care products
**PPP**	Propyl paraben
**ROS**	Reactive oxygen species
**SOD**	Super oxide dismutase
**T**	Testosterone
**TC**	Teratogenic concentration
**TEI**	Teratogenic index
**THs**	Thyroid hormones
**TEDs**	Thyroid endocrine disruptors
**THRs**	Thyroid hormone receptors
**TOI**	Toxicological index
**VTG**	Vitellogenin

## Data Availability

The datasets presented in this study can be found in online repositories. The names of the repository/repositories and accession number(s) can be found in the article/Supplementary Material.
