# Supplementary material for: A systematic review of the toxic potential of parabens in fish
Source: Front Toxicol. 2024 Oct 7;6:1399467. doi: 10.3389/ftox.2024.1399467 (PMC11491439; doi:10.3389/ftox.2024.1399467)
Supplement: Supplementary file 1 [file Table1.docx]

**Supplementary data**

Table; ST1

| Fish | Parabens | Developmental stage | Concentration  /duration | effects | references |
| --- | --- | --- | --- | --- | --- |
| Common carp (*Cyprinus carpio*) | MTP | Fertilized eggs | 0.5;50;500;5000;100,000 µg/L; 96 h | 1. 100% mortality at 100,000 µg/L concentration 2. No induced developmental abnormalities 3. Hatching delay in 50,500, and 5000 µg/L concentration was observed | Medkova et al., (2023) |
|  | PPP | Embryos | 0.1;10; 100; 1000; 100,000 µg/L; 96 h | 1. 100% mortality of the embryos observed when exposed to 100,000 µg/L concentration for 96 h 2. No effects on induction of developmental abnormalities 3. Concentration-dependent hatching delay (50-5000 µg/L) 4. The mRNA expression pattern of *cyp19b* and *gst1* was downregulated in embryos exposed to 0.5 µg/l not in 100 µg/L group | Medkova et al., (2023) |
|  | BTP | Embryos | 0.1;10; 100; 1000; 100,000 µg/L; 96 h | 1. 100% mortality of the embryos observed when exposed to 100,000 µg/L concentration for 96 h 2. No effects on induction of developmental abnormalities or hatching delay | Medkova et al., (2023) |
| Fathead minnows | MTP | Larvae (1 dpf) | 5 concentrations (7.1-136.5 mg/L  (48 h) | 1. Did not affect to cause significant mortality up to 160 mg/L. 2. 48h LC50 >160 mg/L 3. LOEC for larval growth is 25.0 mg/L 4. Hazard quotient for larval growth is 9X10^-5^ | Dobbins et al., (2009) |
|  | ETP | Larvae (1 dpf) | 5 concentrations  (5.5-43.9 mg/L)  (48h) | 1. LC50 (48h) 34.3 mg/L 2. LOEC for larval growth =17 mg/L   Hazard quotient for larval growth =7.8X10^-6^ | Dobbins et al., (2009) |
|  | i-PPP | Larvae (1 dpf) | 5 concentrations (48h) | 1. LC50=17.5 mg/L (48h) 2. LOEC for larval growth =9.0 mg/L 3. Hazard quotient for larval growth = 2.8X10^-5^ | Dobbins et al., (2009) |
|  | PPP | Larvae (1 dpf) | 5 concentrations (5.5-43.9 mg/L)  (48h) | 1. LC50= 9.7 mg/L (48h) 2. LOEC for larval growth =2.5 mg/L 3. Hazard quotient for larval growth = 3.1X10^-5^ | Dobbins et al., (2009) |
|  | i-BTP | Larvae (1 dpf) | 5 concentrations (48h) | 1. LC50=6.9 mg/L (48h) 2. LOEC for larval growth =3.5 mg/L 3. Hazard quotient for larval growth = 1.1X10^-4^ | Dobbins et al., (2009) |
|  | BTP | Larvae (1 dpf) | 5 concentrations  (3.1-24.2 mg/L)  (48h) | 1. LC50=4.2 mg/L (48h) 2. LOEC for larval growth = 1.0 mg/L 3. Hazard quotient for larval growth = 6.5X10^-5^ | Dobbins et al., (2009) |
|  | BNP | Larvae (1 dpf) | 5 concentrations  2.1-16.0 mg/L)  (48h) | 1. LC50=3.3 mg/L (48h) 2. LOEC for larval growth = 1.7 mg/L 3. Hazard quotient for larval growth = 2.3X10^-4^ | Dobbins et al., (2009) |
| Japanese medaka | MTP | Larvae (10 dpf) | 5 different concentrations; for 96 h | i) The 96 h LC50 is 63,000 µg/L (50,000-93,000) | Yamamoto et al., (2011) |
|  | MTP | Adult male (3-month-old) | 40, 200, 1000, 5000, and 25,000 µg/L for 14 days | 1. Plasma VTG level increased after exposure to 780 µg/L (experimental concentration was 1000 µg/L) 2. NOEC for VTG was 160 µg/L (experimental concentration was 200 µg/L) 3. Upregulation of 13 genes including *vtg2, chgL, chgH, esr1* and downregulation of 10 genes occurred in adult male fish exposed to 40 µg/L (actual concentration 10 µg/L) of MTB. | Yamamoto et al., (2011) |
|  | ETP | Larvae (10 dph) | Five different concentrations; 96 h | 1. 96 h LC50 is 14 mg/L | Yamamoto et al., (2011) |
|  | PPP | Embryos (stages 7-8) | 40, 400,1000, 4000 µg/L; exposure durations (stages 7-8 [3-4 hpf]-240 hpf) and evaluated at 76,124,196,244, 316 hpf for embryos. 13 dpf as eleutheroembryos, 28 and 43 dpf as larvae | Embryos   1. Survivability affected in a concentration-dependent manner (no effect in 1000µg/L but reduced in 4000 µg/L) 2. Concentration-dependent significant dilation of the gall bladder seen during embryonic development 3. No effect on p4501A activity while p4501A agonist (β-naphthoflavone) resulted synergistic response in ethoxyresorufin-O-deethylase (EROD) activity.   Larva:  Survivability affected in a concentration-dependent manner (<1000 µg/L) | Gonzalez-Doncel et al., (2014) |
|  | n-PPP | Larvae (10 dph) | Five different concentrations; 96 h | 1. 96 h LC50 is 4.6 mg/L | Yamamoto et al., (2011) |
|  | i-PPP | Larvae (10 dph) | Five different concentrations; 96 h | 1. 96h LC50 is 4.5 mg/L | Yamamoto et al., (2011) |
|  | PPP | Adult (male) | 0.055,0.55, 5.5, and 55 mM/one week | 1. Plasma VTG concentration increased 2. *vtg1, vtg2, chgL, chgH, esr1* and *esr2* mRNAs in liver were upregulated in a concentration-dependent manner.   Androgen receptor mRNA (*ar*) remained unaltered. | Inui et al., (2003) |
|  | *i-*BTP | Larvae  (10 dph) | Six different concentrations; 96 h | 1. 96h LC50 was 4.6 mg/L | Yamamoto et al., (2007) |
|  | *i*-BTP | Adult (2/5 months old males) | 4,20,100,500 µg/L/14 days | 1. Concentration-dependent increase in plasma VTG level in male fish | Yamamoto et al., (2007) |
|  | *n-*BTP | Larvae  (10 dph) | Six different concentrations; 96 h | 1. 96h LC_50_ (determined on 10-day larvae) is 2.9 mg/L | Yamamoto et al., (2007) |
|  | *n*-BTP | Adult (2/5 months old males) | 8,40,200, and 1000 µg/L/14 days | 1. Concentration-dependent increase in plasma VTG level in male fish | Yamamoto et al., (2007) |
|  | BNP | Larvae  (10 dph) | Six different concentrations; 96 h | 1. 96h LC_50_ (is 0.73 mg/L | Yamamoto et al., (2007) |
|  | BNP | Adult (2/5 months old males) | 4,20,100, and 500 µg/L; 14 days | 1. Concentration-dependent increase in plasma VTG level in male fish 2. The number of upregulated genes in male fish by BNP are concentration-dependent 3. The number of downregulated genes in male fish by BNP are nonlinear | Yamamoto et al., (2007) |
|  | BNP | Adult (2/5 months old males) | 4µg/L; 14 days | Gene expression of p53, P4503A40, and chgL increased | Yamamoto et al., (2007) |
| Nile Tilapia | MTP | Adult male (3-5 months old) | 17.1-136.5 mg/L;  48 h | 1. The 48 h LC50=67.11 mg/L | Silva et al., (2018) |
|  | MTP | Adult male (3-5 months old) | 4 mg/L exposed for 6 and 12 days. | 1. Superoxide dismutase (SOD) activity increased after 12 days of exposure 2. Catalase (CAT) activity increased in liver after 6 days exposure, not after 12 days. 3. Glutathione peroxidase (GPx) activity increased in gills after 6 days exposure 4. Glutathione reductase (GR) activity in liver increased after 12 days exposure 5. Glutathione (GSH) level remained unchanged in gills; however, in liver, decreased in 6 days and increased after 12 days exposure. 6. Malondialdehyde (MDA) content in liver and gills remained unaltered. | Silva et al., (2018) |
|  | ETP | Adult male (3-5 months old) | - - - 1. mg/L;  1. h | 1. The 48 h LC50 is 24.08 mg/L (18.70-31.02 mg/L) | Silva et al., (2018) |
|  | ETP | Adult male (3-5 months old) | 4 mg/L; 6 and 12 days | 1. SOD activity increased in the gills after 12 days exposure 2. The CAT activity in gills did not alter either in 6 or 12 days of exposure; in liver, CAT activity increased in 6 days, however, remained unaltered after 12 days of exposure 3. The GPx activity in gills increased in both 6- and 12-days exposure 4. The GR activity in liver increased after 12 days of exposure 5. The GSH content of the gills did not change; in liver, the GSH content increased in both 6 and 12 days of exposure. 6. MDA level did not alter in gills; however, in liver, a decrease was observed after 12 days exposure. | Silva et al., (2018) |
|  | PPP | Adult male (3-5 months old) | 3.1-24.8 mg/L;  48 h | 1. The 48 h LC50 is 17.36 mg/L (14.63-20.61) | Silva et al., (2018) |
|  | PPP | Adult male (3-5 months old) | 4 mg/L; exposed for 6 and 12 days | 1. SOD activity in gills increased after 12 days exposure; liver SOD remained unaltered in fish in both 6- and 12-days exposure periods 2. CAT activities in gills and liver remained unaltered in both 6 and 12 days of exposure 3. GPx activity increased only in gills after 6 days exposure; gills after 12 days of exposure and liver in both 6 and 12 days exposure, the GPx activity remained unaltered. 4. GR activity in gills and liver remained unaltered in fish exposed to PPP in both exposure days (6 and 12 days). 5. GSH content in gills remained unaltered in fish exposed to PPP in both treatment days (6 and 12 days); in liver, the GSH content was initially decreased in 6 days exposure groups, while increased after 12 days of exposure. 6. MDA level remained unaltered in both liver and gills after 6- and 12-days exposures. | Silva et al., (2018) |
|  | BTP | Adult male (3-5 months old) | 2.7-21.5 mg/L.  48 h | 1. LC 50 after 48 h of exposure was 7.80 mg/L (5.38-11.83 mg/L) | Silva et al., (2018) |
|  | BTP | Adult male (3-5 months old) | 4 mg/L; 6- and 12-days exposure | 1. SOD activity increased in gills only after 12 days exposure; in liver, enhancement was observed only in 6 days not in 12 days. 2. CAT activity in both gills and liver remained unresponsive to BTP both at 6- and 12-days exposures 3. GPx activity in gills remained unaltered after 6- and 12-days exposure; in liver, GPx activity increased only after 12 days exposure 4. GR activity remained unaltered in both gills and liver in 6 and 12 days 5. GSH content in gills increased in fish only after 6 days of exposure; however, in liver, GSH content was decreased initially in 6 days and then increased in fish exposed to BTP for 12 days. 6. MDA levels did not alter in gills in fish exposed for 6 or 12 days; in liver, a decrease in MDA content was observed in fish exposed for 12 days. | Silva et al., (2018) |
|  | BTP | Adults | 5,50,500, and 5000 ng/L: 56 days | 1. Increased darker skin pigmentation 2. Gene expression analysis showed that expression of *α-MSH (*upregulation*),* *asip2 (*downregulation*),* significantly changed 3. Reduced dopamine and γ-aminobutyric acid content in brain 4. Significant upregulation of the expression of *arr3a* and *arr3b* and downregulation of *opsin* in a concentration-dependent manner | Liu et al., (2023) |
|  | BNP | Adult male (3-5 months old) | 2.1-16.9 mg/L;  48 h | 1. 48 h LC50 is 7.98 mg/L (5.38-11.83 mg/L) | Silva et al., (2018) |
|  | BNP | Adult male (3-5 months old) | 4 mg/L; exposed for 6 and 12 days. | 1. SOD activity in gills increased after 6 days exposure and continued to increase after 12 days;; in liver, SOD activity remained unaltered in fish exposed to BNP in both 6 and 12 days 2. CAT and GR activities remained unalter in gills and livers of both 6- and 12-days exposed fish 3. GSH content in gills remained unaltered, while increased in liver after 12 days exposure 4. MDA content remained unaltered in both liver and gills in fish exposed to BNP in both 6 and 12 days. | Silva et al., (2018) |
| Rainbow trout | ETP | Juvenile (80-120g) | 100 and 300 mg/kg/ injected 0 and 6 days/ final assays on 12 days. | 1. Dose-dependent induction of serum VTG observed in 6- and 12-days exposure (induced only in 300 mg/kg not in 100 mg/kg). | Pedersen et al., (2000) |
|  | PPP | Juvenile rainbow trout (80-120 g) | 100 and 300 mg/Kg/ injected on 0 and 6 days of experiment/ assessed on 12^th^ day of experiment | 1. Dose-dependent increase in plasma VTG level both on 6 and 12 days of investigation. | Pedersen et al., (2000) |
|  | PPP | Sexually immature rainbow trout | 7-1830 µg/kg/every second day until 10 days orally | 1. Increase in plasma VTG levels 2. ED50 values for increase in VTG synthesis were 35,31, and 22 mg/kg/2day at 3, 6 and 11 day of treatment, respectively | Bjerregaard et al., (2003) |
|  | PPP | Sexually immature rainbow trout | 50 and 225 µg/kg/every second day until 12 days by immersion | 1. Increase in plasma VTG levels is concentration-dependent (increase in 225 µg/L; not in 50 µg/L) 2. Accumulation of PPP in liver and muscle of fish exposed to 225 µg/L for 12 days, was investigated. The accumulated PPP was 6700 µg/kg liver and 870 µg/kg muscle. 3. Half-lives of PPP in liver was 8.6 h and in muscle 1.5 h. | Bjerregaard et al., (2003) |
|  | BTP | Sexually immature rainbow trout | /4 -74 mg/kg/every second day until 10 days orally | 1. Dose and time-dependent increase in plasma VTG levels 2. ED50 for VTG response is 10.5 mg/kg/2d | Alslev et al., (2005) |
|  | BTP | Sexually immature rainbow trout | 35 and 201µg/L for 12 days by immersion | 1. Increase in plasma VTG levels is concentration-dependent (increase in 201 µg/L; not in 35 µg/L) 2. A positive correlation exits in the concentration of BTP and the VTG in the plasma 3. Uptake of BTP from the environment to the body of the fish was 13 mg/kg/day for fish exposed to 35 µg/L and 78 mg/kg/day in fish exposed to 201 µg/L | Alslev et al., (2005) |
|  | BTP |  | 5,50,500, 5000 ng/L/56 days | 1. Induced darker skin pigmentation in a concentration-dependent manner 2. The gene expression related to pigmentation (*α-MSH* and *Asip2*) changed 3. Reduced dopamine and γ-aminobutyric acid content in the brain (related to the synthesis of *α-MSH)* 4. Expression of *Arr3a* and *Arr3b* was upregulated, however, *Opsin* expression downregulated in a concentration-dependent manner 5. Inhibited phototransduction from the retina to the brain |  |
| Zebrafish | MTP | Embryos (4-6 hpf) | 100,200,400,800,1000 µM; 96 h | 1. 96 h LC50 value is 65 mg/L 2. Concentration-dependent decrease in heart rates 3. Concentration-dependent decrease in hatching rates 4. Morphological abnormalities (pericardial edema, bent spine, blood cell accumulation) increased at the concentration-dependent manner 5. *vtg1* expression enhanced | Dambal et al., (2017) |
|  | MTP | embryos | 50 mg/L; 5 days | 1. LC50= 50 mg/L 2. Tail defects, pericardial edema, and pigmentation defects observed (increased with MTP exposure) 3. Inhibition of locomotor activity 4. Reduced GST and NO levels 5. Lipid peroxidation (LPO) enhanced 6. MDA content enhanced 7. Increased expression of *ccdn1* (proto-oncogene) and *myca* (cellular proliferation) mRNAs | Ates et al., (2018) |
|  | MTP | Embryos  (2 hpf) | 1,10,25,50,100 and 200µM; 120 hpf | 1. No significant mortality was observed 2. Hatchig delay was not observed 3. Concentration-dependent malformation in embryos/larvae at 96 hpf (pericardial edema and spinal defects) 4. Downregulated stress-related microsomal glutathione S transferase (*mgs*t), and glutathione S transferase (*gst*) genes by MTP 5. *cat* and *sod3* did not show any significant change 6. *hsp70* and metallothionein (*mt1*) did not show any significant changes 7. no alteration in the expression of *bax* and *bcl2* 8. a decreased expression of growth arrest and DNA damage inducible alpha (*gadd45a*) 9. expression of *tnfα* and *il8* was affected by MTP exposure 10. downregulation of *ldlr* occurred. 11. Repressed the expression of *ar* | Bereketoglu and Pradhan (2019) |
|  | MTP | Embryos | 0.1, 1, 10, 100 ppb; until 6 dpf | 1. Concentration-dependent decline in hatching rates 2. Concentration-dependent decrease in heart rates (48 hpf) 3. Anxiety-like behavior significantly higher 4. No significant difference in scototaxis (light-dark preferences) 5. Concentration-dependent decrease in AChE activity 6. Whole body cortisol level increased in a concentration-dependent manner | Raja et al., (2019) |
|  | MTP | Fertilized eggs  (1-3 hpf) | 1, 10, 30, 60, 80 mg/L;  96 hpf | 1. 96 h LC50 is 72.67 mg/L (BMDL-BMDU=40.8-57.4 mg/L 2. Embryos exposed to 60-80 mg/L died by 24 h. 3. TI= 16-26.5 mg/L 4. Significant reduction in hatching rates (72 hpf) 5. Notochord curvature was increased significantly in a concentration-dependent manner 6. Reduced heartbeats, blood stasis, and reduction in blood circulation (48 h) in a concentration-dependent manner 7. Concentration- dependent development of pericardial edema and yolk sac edema (48hpf) | Merola et al., (2020b) |
|  | MTP | Embryos  (1 hpf) | 100, 1000, 10000 µg/L; 4-6 days | 1. No significant effect on thigmotaxis 2. No significant effect on startle response 3. Did not affect photic entrainment of locomotor activity | Merola et al., (2021) |
|  | MTP | Embryos (2 hpf) | 1,5, 20, 100, 200 µM;120h | 1. Significant reduction in body length and heartrates 2. Did not exert any significant influence on the survival rate of zebrafish larvae (120 hpf) 3. Concentration-dependent decrease in hatching rate (200 µM) 4. Concentration-dependent induction in malformations in cardiac edema and spinal curvature (100 and 200 µM) 5. Decrease in both T3 and T4 levels 6. Gene expression analysis indicated the transcriptional level of transthyretin (*ttr*) was significantly decreased in larvae exposed to 200 µM | Liang et al., (2022) |
|  | MTP | Embryos (2 hpf) | 20, 100, 200 µM/120h | 1. The vtg concentration in the larvae increased in a concentration-dependent manner (120 hpf) 2. The level of T was downregulated in all concentrations of MP (20-200 µM) used. | Liang et al., (2023a) |
|  | MTP | Embryos (2 hpf) | 20, 100, 200 µM/120 hpf | 1. Inhibited the total movement and mean velocity in a concentration-dependent manner (significantly differ from controls only in the larvae exposed to 200 µM) 2. Significant increase in AChE enzyme activity in a concentration-dependent manner 3. Concentration-dependent increase in the cortisol levels and decrease in the ACTH levels in all treatment groups. 4. The downregulation of *gr, mr* and *crhr2* genes and upregulation in *pmoc* genes in the HPI axis (hypothalamus-pituitary-interrenal gland) | Liang et al., (2023b) |
|  | MTP | Embryos | 0.5, 50, 500, 5000,100,000 µg/L;  96 hpf | 1. Caused 33% mortality at the concentrations 100,000 µg/L concentration 2. Significantly delayed hatching in a concentration-dependent manner 3. Downregulation of *hsp70l* and *hsp90* | Medkova et al., (2023) |
|  | MTP | Embryos (2-3 hpf) | 160 µM at 56 hpf | 1. Heart rate increased 2. Cardiac output remained unaltered 3. Stroke volume remained unaltered | Shi et al. (2023) |
|  | MTP | Embryos  (4hpf) | 5,10,20, 40, 80, 150, 300 µM; 120 hpf | 1. LC50 (120 hpf) was 468.14 µM (309.51-626.78 µM) 2. EC50 (120 hpf) =255.61 µm (220.7-290.4 µM) 3. Teratogenic index (TEI) 1.8 (120 hpf) 4. Mortality point of departure (M-POD) is 154.1 µM (4-120hpf) 5. Malformation-based point of departure (Mal-POD) was 196.6 µM 6. No significant difference in locomotor activity during both light and dark phases at all tested concentrations 7. Total differentially expressed genes (DEG) were 166 of which 119 downregulated and 47 upregulated genes were observed. | Tran et al., (2023) |
|  | MTP | Larvae and adults | 60, 102, 173,294, 500mg/l for larvae, exposed for 168 h.  Adults 30,48,67.5, 101 and 105 mg/L exposed for 96h | 1. The LC50 was 105.09 mg/L for adults and 211.12 mg/L for larvae. 2. In adults (50 mg/L), ethoxyresorufin O-deethylase activity (EROD) in gills significantly decreased. 3. LPO did not differ in liver, augmented in gills 4. The frequency of micronuclei in erythrocytes significantly increased (adults, exposed to 50 mg/L) 5. Gut microbiota did not vary | De Carvalho Penha et al., (2021) |
|  | MTP | Adult (male) | 0.001, 0.01,1.0, 10.0 mg/L: 21 days | 1. The length and weight of the fish remained unaltered 2. Concentration-dependent decrease in GSI 3. General atrophy, multinucleated gonocytes, impaired germ cells, spermatogonial proliferation, Leydig cell proliferation, interstitial fibrosis and apoptosis of Sertoli cells. | Hassanzadeh, (2017) |
|  | MTP | Adult (males and females) | 1,10, 110 ppb ;30 days | 1. LC 50 was 1.102 ppb for MTP in adult zebrafish (1.102 µg/L) 2. Increase in average distance travelled by both male and female fish on 15-day exposure; while significant decreased in average distance travelled by both male and female fish exposed for 30 days 3. Swimming speed in females increased in both 15 and 30 days in fish exposed to MTP (all concentrations); in males, increase in swimming speed was observed in lower concentration groups and decrease in highest concentrations on 15 days exposure; in 30 days exposure swimming speed in male fish deceased in all exposure groups when compared with controls 4. More anxiety-like behavior observed in females than male 5. Concentration-dependent reduction in AChE activity 6. Concentration-dependent increase in serotonin level in female fish while in male fish concentration dependent reduction in serotonin level 7. Expression of *hif-1α*, *tnn2,* *pax-6b*, *ntrk2a*, were altered (mostly downregulated in higher concentrations). | Thakkar et al., (2022) |
|  | MTP | Adults | 1,3, and 10 µg/L/ 28 days | 1. Disrupt the composition and diversity of gut microbial community 2. Increased the body length and weight of the female fish 3. In intestine of males, increase in goblet cell density, tight junction protein 2 (TJP2), and serotonin concentrations 4. In females lower density in intestinal goblet cells, inhibited expression of TJP2 and reduced the concentration of serotonin but upregulated the expression of proinflamatory cytokines. 5. Intestinal catalase activity enhanced. | Hu et al., (2022b) |
|  | MTP | Adults (4 months) | 1,3,10 µg/L/28 days | 1. Significant decrease in hepatosomatic index (HSI) in female fish exposed to 1 and 10 µg/L 2. Induced hepatocellular vacuolization 3. Significant inconsistent enhancement of alanine aminotransferase (ALT) activity (only in 1µg/L) in both males and females 4. Liver ROS in females significantly higher in fish exposed to 10 µg/L, which was accompanied by inhibition of catalase (CAT) activity (3 µg/L) 5. GSH content in male liver decreased in a concentration-dependent manner, however, GPx activity in both male and females enhanced in a nonlinear fashion 6. In male liver, upregulation of peroxisome proliferator-activated receptor gene (*pparα*) occurred in fish exposed to 3 and 10 µg/L 7. Farnesoid X receptor gene *nr1h4* was downregulated in male fish in a concentration-dependent manner 8. Inconsistent upregulation of sterol regulatory element binding proteins (srebp1) by MTP (1 and 10 µg/L) in male liver decreased in a concentration-dependent manner 9. The free fatty acid content in male liver 10. In female liver concentration-dependent downregulation of PARP, FAX and SREBP genes observed. 11. The intestinal tissue contains significantly higher concentrations of triglycerides (TG) but lower concentration of glycerol, and free fatty acids (FFA) in male fish exposed to MTP 12. In blood the concentration of TG, total cholesterol (TCHO) HDL-cholesterol (HDL-C), LDL-Cholesterol (LDL-C) decreased by MTP exposure 13. In females, concentrations of TG in intestine, blood, and liver tissues, were significantly decreased after MTP exposure; moreover, concentrations of TCHO and HDL-C were altered inconstantly 14. Higher level of cortisol was observed in male liver | Hu et al., (2022a) |
|  | MTP | Adults | 1,3,10 µg/L/28 days | Significant decrease in cranio-somatic index (CSI) in female fish exposed to 3 µg/L MTP   1. A concentration-dependent increase in ROS in male brain and a concurrent decrease in CAT activity 2. Males exposed to 1 µg/L reduced GSH content in brain and enhanced GPx activity 3. Lower extents of MDA and LPO were accumulated in both male and female brains 4. Neural proteins in male brains exposed to 10 µg/L (*isv2ca, syncripl, rims3, grik2, thrap3b*) and in females, (*neflb, gap43, olfm1b, syap1, chm1, nlgn2a, rs1a, thrap3b*) were altered 5. Enhanced the glutamate content in male brain but decreased in female brain 6. AChE activity remained unaltered 7. Downregulation of α1 tubulin transcripts in both male and female brains of zebrafish by MTP exposure 8. Decreased blood cortisol concentrations in both males and females 9. Downregulation of corticotropin releasing hormone (*crh*) and corticotropin-releasing hormone binding protein (*crhbp*) genes in the brain of both male and female zebrafish 10. disruption of immune system genes including *c4, igl4v8, cd79a, igbp1, cd59, c5* in brain of female fish 11. significant increase in blood-brain-barrier protein (BBB) in female brain by MTP (10 µg/L) exposure 12. significant increase in LPS endotoxin concentration in male brain by MTP exposure (10 µg/L), however decreased in females (3-10 µg/L) 13. transcription of *il1β* and *il6* were upregulated in male brain | Hu et al., (2023a) |
|  | MTP | Adults | 1.3, 10 µg/L/ 28 days | 1. increased GSI in a concentration-dependent manner 2. spermatogenesis and oogenesis were blocked 3. parental exposure induced developmental deficits in larvae by increasing mortality, stimulating precocious hatching, and elevating heart rates. 4. Blood concentrations of E2, T, and 11-keto testosterone were consistently lowered 5. Transcriptional results showed that disruption in the hypothalamus-pituitary-gonadal axis 6. Hepatic VTG downregulated | Hu et al., (2023b) |
|  | ETP | Embryos | 1,5,10,20, and 30 mg/L/96 h | 1. LC50 values at 96h was 20.86 mg/L; benchmark dose (BMD) was 10.8-17.4 mg/L 2. 85% mortality was observed in embryos exposed to 30 mg/L at 96 hpf 3. Significant reduction in hatching rate at 72 hpf 4. Concentration and time-dependent malformation induced reduction in blood stasis, pericardial edema, misshaped yolk sac and deformed notochord 5. Defects in pectoral fin development (concentration and time-dependent) 6. Concentration and time-dependent induction in behavioral abnormalities (treambling of head, pectoral fins, spinal cord, and circling behavior | Merola et al., (2020a) |
|  | ETP | Embryos (6 hpf) | 0.1, 0.5, 1, 2, 3, 4, 5, 6,10,15,18,20, 25,30,42,50,80, 100 mg/L/embryos 48-96hpf; | 1. Calculated LC50 is 28.70 mg/L 2. Teratogenic concentration (TC50) is 20.63 mg/L 3. Induced abnormal cardiac function (heart rates) and morphology (pericardial effusion) 4. Disrupts retinoic acid signaling pathway related to original cardiac catheter development 5. Inhibition of gene expression related to myocardial contraction 6. Orientation development disturbance of heart tube (oriented toward the right or middle side) 7. Gene expression analysis indicated that ETP (20 mg/L) upregulated 1302 genes and downregulated 2482 genes. 8. The key genes related to apoptosis (*casp3, casp9, afap111b ,gadd 45ga, cflts*) were upregulated after ETP (20 mg/L) exposure 9. Among the downregulated genes, the expression of *ttc36*, a gene associated with heart tube orientation, was also downregulated | Fan et al., (2022) |
|  | ETP | Embryos | 50, 500, 5000 µg/L/ exposed until 4 dpf/ behavioral analysis was on 4,5, and 6 dpf | 1. Increased anxiety-like behavior in a concentration-dependent manner 2. Did not influence visual startle response and the photic synchronization of circadian rhythms | Merola et al., (2021) |
|  | ETP | Embryos | 1, 5, 10, 20, 30mg/L;  96 hpf | 1. LC50 is 20.86 mg/L 2. Reduced heartbeat, reduction in blood circulation, blood stasis, pericardial edema, deformed notochord, misshaped yolk sac 3. Induced behavioral changes (trembling of head, pectoral fins and spinal cords) | Merola et al., (2020a) |
|  | ETP | Embryos (2hpf) | 1,5, 20,50, 100 µM,  120 hpf | 1. Body length and heart rate reduced in a concentration-dependent manner 2. Significant reduction in the survival rate of the embryos exposed to 200 µM. 3. Hatching was reduced in embryos exposed to 50-100 µM ETP 4. Malformation rate (cardiovascular edema and spinal curvature) was significantly increased in a concentration-dependent manner 5. Significant reduction in T3 and T4 contents of the larvae exposed to 50-100 µM ETP 6. Induced expression of *tshβ* in a larvae exposed to 100 µM ETP 7. Induced significant increase in trα (20-100 µM) ETP 8. Decreased in the expression of *pax8* (20-100 µM) and *ttr* (50-100 µM) | Liang et al., (2022) |
|  | ETP | Embryos (2 hpf) | 20,50, 100 µM/120 hpf | 1. Vtg content enhanced in 20 and 50 µM concentrations 2. The level of T was down regulated (2-100 µM) | Liang et al (2023a) |
|  | ETP | Embryos (2 hpf) | 20,50, 100 µm/120 hpf | 1. Significant inhibition in the total movement distance covered by larvae in lower concentrations (20 and 50 µM) 2. Significant increase in AChE activity in larvae (120 hpf) exposed to 50 µM ETP. 3. Significant concentration-dependent decrease in ACTH level 4. Significant concentration-dependent enhancement in the cortisol level 5. Significant inconsistent concentration-dependent downregulation in the mRNA levels of *mr, crh2, crhbp* (except *gr*) were observed. | Liang et al., (2023b) |
|  | ETP | Embryos (4hpf) | 5,10,20, 40, 80, 150, 300 µM; 120 hpf | 1. LC50= 196 µM (138.27-257.384 µM) (120 hpf) 2. EC50=146.8 µM   (123.4-170.27 µM)   1. TEI=1.3 (ratio of LC50/EC50) 2. Hyperactivity was observed in ETP (~72.6 µM) in the acclimation and reduction darkness phases 3. 414 genes were differentially expressed of which 352 genes were upregulated and 67 genes were downregulated. | Tran et al., (2023) |
|  | PPP | Embryos (2 hpf) | 1,2, 5, 10 and 20 µM.  120hpf | 1. Concentration-dependent decrease in body length and heart rates 2. Significant decrease in survival rate and hatching of the embryos 3. Morphological alteration resulted malformation in embryos/larvae (yolk sac edema/ tail curvature) increased (10 and 20 µM) 4. Significant reduction in the T3 and T4 levels in embryos (5-20 µM) 5. Upregulation of *tshβ* (10-20 µM) 6. Upregulation of *tg*, *nis*, and *dio1* (5-10 µM) 7. Upregulation of *nkx2.1*, *ttr*, and *ugt1ab* only in larvae exposed to 5µM group 8. Upregulation of *trα* was occurred in 10 µM group 9. Downregulation of *pax8* was occurred in larvae exposed to 5 µM group | Liang et al., (2022) |
|  | PPP | Embryos  (2-3 hpf) | 10 and 1000 µg/L; 24h | 1. No significant changes in total and individual oxysterols compared to control groups, however, 27-OH showed a reduction at 24h exposure | Merola et al., (2022) |
|  | PPP | Embryos (2 hpf) | 2,5, and 10 µM/120hpf | 1. Vtg content enhanced in 2 µM concentration 2. The level of T was down regulated (2-10µM) | Liang et al (2023a) |
|  | PPP | Embryos (2 hpf) | 2,5, and 10 µM/120hpf | 1. Significant concentration-dependent decrease in the total distance covered by the 120 hpf larvae by swimming and the mean velocity (significant reduction was observed in larvae exposed to 10 µM of PPP) 2. AChE activity remained unaltered. 3. Concentration-dependent decrease in the level of ACTH and enhancement in the level of cortisol 4. Inconsistent concentration-dependent down regulation of *gr, mr, crhr2, crhbp* in the HPI axis. No significant effect was observed in the mRNA expression level of *pmoc* gene. | Liang et al (2023b) |
|  | PPP | Embryos | 10, 100, 1000, 1500, 6000,8500, 10,000 µg/L/8, 32, and 80hpf | 1. NOEC=   1000 µg/L (Exposure to 10 mg/L is lethal)   1. Reduced heartrate in time and concentration-dependent manner 2. Concentration and time-dependent abnormalities observed in eyes, head, pericardial edema, yolk sac, and tail development | Torres et al., (2016) |
|  | PPP | Embryos (2 hpf) | 2hpf/1,10,25, 50,100, and 200 µM/2hpf-120hpf | 1. Concentration-dependent mortality (100% mortality in concentration above 10µM) 2. Hatching delay observed (10 µM) 3. Malformed embryos observed (pericardial edema and spinal defects at 10 µM concentration) 4. Gene expression analysis (1 and 10 µM) showed decrease expression of nuclear factor, *erythroid 2-like 2a (nrf2),* *kelch- ECH-associated protein 1 (keap1),* and sod1 5. Downregulation of microsomal glutathione S-transferase (*mgst*) and glutathione S-transferase (*gst*) 6. *Catalase* and *sod3* did not alter 7. Significant upregulation of *hsp70* and metallothionein 1 (*met1*); however, no alteration was observed in *met2* gene. 8. Downregulation of caspase 3a (*casp 3a*), death associated protein 3 (dap3), BCL2apoptosis regulator 2 (*bcl2*), while increased BCL2 associated X regulator (*bax*) 9. Downregulation of cyclin-dependent kinase inhibitor 1A (p21) and induction of mitogen activated protein kinase 14a (*p38*) was observed (10 µM) 10. Decreased expression of growth arrest and DNA- damage inducible alpha (*gadd45a*) was observed 11. Downregulation of RAD51 recombinase (*rad51*) and APEX nuclease (multifunctional DNA repair enzyme)1 (*apex 1*) and upregulation in xeroderma pigmentosum, complementation group C (*xpc*) by PPP was observed 12. The expression of *tnfα* and *il8* was altered significantly 13. Downregulation of apolipoprotein genes involved in fatty acid transport (*apoab, apoeb, apoa4*) and fatty acid synthesis (*fasn*) 14. Downregulation of low-density lipoprotein receptor (*ldlr*) by 10 µM PP 15. Upregulation of *lipase, hepatic (lipc*) was occurred (both 1 and 10 µM) 16. Repressed the expression of *ar* and upregulated the expression of *esr2a* 17. Increased expression of thyroid hormone receptor αa (*thraa*) and thyroid hormone receptor β (*thrb*) | Bereketoglu and Pradhan (2019) |
|  | PPP | Embryos | 1,2,4,6,8 mg/L.  96hpf | 1. LC50 is 3.98 mg/L 2. Enlarged and misshaped yolk sac 3. Reduction in hatching rates (evaluated at 72 hpf) 4. All embryos died at 96 hpf when exposed to 8 mg/L 5. malformation of the larvae (reduced heart beats, blood stasis, reduction in blood circulation, pericardial edema, enlarged yolk sac, deformed tail) induced in a concentration-dependent manner 6. Hyperexcitability 7. Reduction in head size and swim bladder 8. Decrease in neutral lipid metabolism in yolk 9. Alteration in phospholipid metabolism (reduction in PLA2 enzyme activity) both in body and yolk sac 10. Deformed neurocranium | Perugini et al., (2020) |
|  | PPP | Embryos (2 hpf) | 0.1,1, and 10 µg/L/exposed for 2-96 hpf. | 1. Hatching rate declined only at 48 hpf, not in 24hpf, 72 hpf and 96 hpf 2. Survivability of the embryos seem to be time and concentration-dependent 3. Concentration-dependent induction in the malformation (bent spine, yolk sac edema, pericardial edema, red blood cell accumulation) of the embryos occurred. 4. Significant concertation-dependent decrease in heart rate (observed on 96 hpf) 5. Trigger anxiety-like behavior in larvae (6dpf) (concentration-dependent reduction in the time spent in the light zone). 6. The exploratory behavior (the number of transitions between light and dark compartments) significantly reduced in a concentration-dependent manner) 7. Significant increase in intracellular ROS and LP 8. SOD, CAT, GPx, GST, GSH activity/concentration in the head region of the larvae decreased/suppressed 9. Suppression of AChE activity and increase in nitric oxide production (concentration-dependent) | Lite et al., (2022) |
|  | PPP | Embryos | 0.1;10;100;1000; 100,000 µg/L/96 hpf | 1. 100 % mortality was observed in embryos exposed to 100,000 µg/L 2. Did not induce any developmental abnormalities as observed at 72 hpf 3. Delayed hatching in a concentration-dependent manner (only in 1000 µg/L) 4. Upregulation of *hsp70l* mRNA exposed to 0.1 µg/L 5. Expression of gstp2 was upregulated after exposure to 0.1 µg/L 6. Upregulation of both *cyp17a1* and *cyp19a1a* embryos exposed to 0.1 and 100 µg/L | Medkova et al., (2023) |
|  | PPP | Embryos  (4hpf) | 5,10, 20, 0, 80, 150, 300 µM; 120 hpf | 1. Calculated 120h LC50= 61.8 µM (39.5-84.2 µM) 2. Calculated 120h EC50=40.8 µm (35.5-46.1 µM) 3. TI= 1.5 4. No significant differences in locomotor activities during both light and light phases at all tested concentrations. 5. 315 genes were differentially regulated of which 181 gene s were upregulated and 134 genes were downregulated | Tran et al., (2023) |
|  | PPP | Embryos and larvae | 10 and 1000 µg/L; 3hpf- 4 dpf | 1. Thigmotaxis was decreased in larvae exposed to 1000 µg/L during embryogenesis. 2. Thigmotaxis was increased in juveniles exposed to 10 µg/L embryologically 3. Expression of *shank3a* and *gad1b* was repressed by PPP (10-1000 µg/L) 4. Proteomics analysis indicated alterations related to brain development and lipid metabolism. | Merola et al., (2024) |
|  | PPP | Larvae (20 dph) | 0.1, 0.4, 0.9 mg/L; 20 days | 1. Decline in vitellogenin production (whole body) | Mikula et al., (2006a, 2006b) |
|  | PPP | Larvae (20 dph) | 500, 1000, and 2000 mg/kg via food; 20 days and 45 days | 1. Length and weight did not alter 2. No significant difference in whole body vtg content 3. Sex ratio female biased (significant only in 500 mg/kg dose) | Mikula et al., (2009) |
|  | BTP | Embryos | 0.1, 1, and 10 µg/L/ 2hpf- 96 hpf | 1. Hatching rate, survivability   and malformation varied.   1. Trigger anxiety-like behavior in larvae 2. Significant increase in intracellular ROS and LP 3. SOD, CAT, GPx, GST activity in the head region of the larvae suppressed 4. Suppression of AChE activity and increase in nitric oxide production | Lite et al., (2022) |
|  | BTP | Embryos (2 hpf) | 0.5, 1, 2, 5 and 10 µM/120 hpf | 1. Concentration-dependent decrease in body length and heartrate (5-10 µM) 2. Nonlethal concentration of BTP in zebrafish was 10 µM 3. Concentration-dependent decrease in hatching (72 hpf) and heart rates (120hpf) 4. Concentration-dependent enhancement in the malformation rate (cardiac edema and bent tail) induced in larvae (120 hpf) 5. Significant decrease in T3 (2-10 µM) and T4 (5-10 µM) levels by BTU (120 hpf) 6. Significant downregulation of *crh, trh, tshβ, nkx2.1, hhex, ttr, dio1, dio2*, and *ugt1ab* (2-10 µM) 7. Significant upregulation was observed in *trα* mRNA in larvae (120 hpf) exposed to 10 µM BTU. | Liang et al., (2022) |
|  | BTP | Embryos (2 hpf) | 1, 2 and 5 µM/120 hpf | 1. Vtg content enhanced in 1 and 2 µM concentration 2. The level of T was upregulated in 1 µM and down regulated in 2µM concentrations 3. The level of estrogen (E2) was elevated exposed to 1 and 2 µM concentrations | Liang et al (2023a) |
|  | BTP | Embryos (2 hpf) | 1, 2 and 5 µM.  120 hpf | 1. The total movement distance covered by the zebrafish larvae (120 hpf) and the mean velocity significantly reduced by BTP (only in 5 µM exposure group) 2. No obvious change in the AChE activity in the larvae (120 hpf) was exposed to any of the concentration of BTP used in this study. 3. No effect on the ACTH concentration, however, the cortisol level in BTP-exposed larvae (5 µM) reduced significantly 4. Inconsistent concentration-dependent down regulation of *gr, mr, crhr2, crhbp* in the HPI axis. No significant effect was observed in the mRNA expression level of *pmoc* gene. | Liang et al., (2023b) |
|  | BTP | Embryos | 0.25,0.5,1,2.5, and 5 mg/L; 96h | 1. LC50 is 2.34 mg/L: benchmark dose (BMD) 0.91-1.92 mg/L (BMD confidence interval) 2. 100 % embryos died after 48 hpf exposed to 5 mg/L BTP 3. Hatching rate altered in a nonlinear fashion. 4. Concentration-dependent reduction on heartbeats, blood circulation, blood stasis, pericardial edema, deformed notochord, and misshaped yolk was observed in embryos during 72-96 hpf of development 5. Defects in pectoral fin development 6. Induced behavioral changes (trembling of head, pectoral fins and spinal cords) | Merola et al., (2020a) |
|  | BTP | Embryos | 5, 50, 500 µg/L/ exposed until 4 dpf/behavioral analysis was on 4,5, and 6 dpf | 1. Increased anxiety-like behavior in a concentration-dependent manner 2. Did not influence visual startle response and the photic synchronization of circadian rhythms | Merola et al., (2021) |
|  | BTP | Embryos | 0.1; 10; 100; 1000; 100,000 µg/L; 96 h | 1. Induced 100% mortality in embryos exposed to 100,000 µg/L 2. Cardiac edema and blood clots were observed in embryos in a concentration-dependent manner (1000 and 100,000 µg/L) 3. Induced hatching delay in a concentration-dependent manner   (72 hpf)   1. Upregulation of hsp70l in embryos exposed to 0.1 and 100 µg/L 2. Upregulation of *cyp19a1a* was observed in embryos exposed only to 0.1 µg/L | Medkova et al., (2023) |
|  | BTP | embryos | 0.1,0.25.0.5, 0.75, 1,2,4,8, and 16 mg/L; 120 hpf | i) LC50 values decreases over time; (24h LC50=10.77 mg/L; 48 h LC50=4.208 mg/L, 72 h LC50=1.953 mg/L; 96 h LC50=1.359 mg/L; 120 h LC50=0.966 mg/L)  ii) Induced pericardial edema and ocular edema (0.5, 0.75, 1 mg/L) at 120 hpf.   1. The length, eyes, otoliths were significantly smaller 2. Hatching rate reduced significantly 3. Maxillofacial cartilage deformed severely deformed 4. Length of ceratohyal bone significantly reduced. 5. The activities of catalase (CAT), and superoxide dismutase (SOD) reduced significantly, however, the activity of malondialdehyde (MDA) significantly elevated 6. The activity of alkaline phosphatase is reduced significantly (marker enzyme of osteoblast activity) 7. Downregulation of the expression of *sox9a, sox9b* and *col2a1a* genes (chondrocyte marker genes) | Li et al., (2023) |
|  | BTP | Embryos  (6hph) | 0.6, 1.2, 1.8 mg/L; until 72 hpf | 1. LC50 is 2.74 mg/L (72hpf) 2. Embryos exhibited pathological effects including, spinal curvature, reduced eye size, and yolk sac edema, but no mortality 3. Significant decrease in heart rates 4. Concentrative-dependent pericardial edema and linearization was observed and showed cardiac impairment by BTP. 5. Impairs the cardiac contraction ability and cardiac output   in a concentration-dependent manner   1. Reduced blood cell counts in heart in a concentration-dependent manner and induced systolic heart failure 2. Decrease in the expression of *klf2a* (the endocardial flow response gene) 3. The heart failure genes, natriuretic peptides A and B (*nppa* and *nppb*) were significantly upregulated 4. Induced apoptosis in heart 5. Damaged endocardial and atrioventricular valves by activating the endogenous apoptotic pathways. 6. Impaired calcium homeostasis 7. Depleted cardiac-resident macrophages 8. Cardiac immune system became non-responsive 9. Induced oxidative stress. | Zhu et al., (2023) |
|  | BTP | Embryos  (*Tg(ins:GFP)*] (3hpf; mid-blastula transition) | 250, 500, 1000, 3000 nM;  3h-7 dpf | 1. LOEC for islet variant morphology was 250 nM and   NOEC was 125 nM   1. Islet area increased significantly 2. Beta (β) cell area of the islet appear more dispersed, fragmented beta cells,and ectopic beta cells emerge away from the primary islet. 3. Islet fragmentation decreased over time 4. Deformity index (pericardial edema, yolk sac utilization, intestinal effusion, craniofacial malformations and spinal malformations) increased in a concentration-dependent manner 5. No significant relationship was observed between BTP concentration and yolk sac area. 6. Body length of the embryos did not affect with the tested BTU concentrations 7. Swim bladder inflation was significantly decreased by BTP in a concentration-dependent manner. 8. GSH increased in a concentration-dependent manner 9. No alteration in the expression of *preproinsulin a (insa) glugagon a (gcga) ghrelin (ghrl),* and *somatostatin 2 (sst2).* However, pancreatic duodenal homeobox 1 (*pdx 1*) was down regulated after BTP exposure. 10. The expression of glutathione-S-transferases (*gstp* and *gsta1*) modestly altered (*gstp* upregulated slightly, *gsta1* downregulated) 11. No effect on glutamate-cysteine ligase (*gclm*), however *gclc* were modestly downregulated by BTP 12. Downregulation of glutathione synthase (*gss*) was observed by BTP exposure in a concentration-dependent manner. | Brown et al., (2018) |
|  | BTP | Adult (male) | 0.01, 0.1, 1.0 mg/L; 28 days | 1. BTP penetrated the   Blood brain barrier and impaired neurobehavior in photosensitivity and memory in a concentration-dependent manner.   1. RNA-seq analysis identified significant effect of BTP on phototransduction, tight junctions, neuroactive ligand receptor activity 2. Cortisol increased and allopregnenolone levels decreased after BTP exposure 3. Induced alterations in neurotransmitters belong to histaminergic, cholinergic, dopaminergic, serotonergic, and GABAergic systems. | Kim et al., (2022) |
