# Supplementary material for: A systematic review of the toxic potential of parabens in fish
Source: Front Toxicol. 2024 Oct 7;6:1399467. doi: 10.3389/ftox.2024.1399467 (PMC11491439; doi:10.3389/ftox.2024.1399467)
Supplement: Supplementary file 2 [file DataSheet1.pdf]

Supplementary data

Table; ST1

Fish	Parabens	Developmental stage	Concentration /duration	effects	references
Common carp (*Cyprinus carpio*)	MTP	Fertilized eggs	0.5;50;500;5000;100,000 µg/L; 96 h	i) 100% mortality at 100,000 µg/L concentration<br>ii) No induced developmental abnormalities<br>iii) Hatching delay in 50,500, and 5000 µg/L concentration was observed	Medkova et al., (2023)
	PPP	Embryos	0.1;10; 100; 1000; 100,000 µg/L; 96 h	i) 100% mortality of the embryos observed when exposed to 100,000 µg/L concentration for 96 h<br>ii) No effects on induction of	Medkova et al., (2023)

				developmental abnormalities<br>iii) Concentration-dependent hatching delay (50-5000 µg/L)<br>iv) The mRNA expression pattern of *cyp19b* and *gst1* was downregulated in embryos exposed to 0.5 µg/l not in 100 µg/L group	
	BTP	Embryos	0.1;10; 100; 1000; 100,000 µg/L; 96 h	i) 100% mortality of the embryos observed when exposed to 100,000 µg/L concentration for 96 h<br>ii) No effects on induction of developmental abnormalities or hatching delay	Medkova et al., (2023)

Fathead minnows	MTP	Larvae (1 dpf)	5 concentrations (7.1-136.5 mg/L<br>(48 h)	i) Did not affect to cause significant mortality up to 160 mg/L.<br>ii) 48h LC50 >160 mg/L<br>iii) LOEC for larval growth is 25.0 mg/L<br>i) Hazard quotient for larval growth is $9X10^{-5}$	Dobbins et al., (2009)
	ETP	Larvae (1 dpf)	5 concentrations (5.5-43.9 mg/L)<br>(48h)	i) LC50 (48h) 34.3 mg/L<br>ii) LOEC for larval growth =17 mg/L<br>Hazard quotient for larval growth $=7.8X10^{-6}$	Dobbins et al., (2009)
	i-PPP	Larvae (1 dpf)	5 concentrations (48h)	iii) LC50=17.5 mg/L (48h)<br>iv) LOEC for larval growth =9.0 mg/L<br>i) Hazard quotient for larval growth = $2.8X10^{-5}$	Dobbins et al., (2009)

	PPP	Larvae (1 dpf)	5 concentrations (5.5-43.9 mg/L) (48h)	i) LC50= 9.7 mg/L (48h)<br>ii) LOEC for larval growth =2.5 mg/L<br>i) Hazard quotient for larval growth = $3.1X10^{-5}$	Dobbins et al., (2009)
	i-BTP	Larvae (1 dpf)	5 concentrations (48h)	i) LC50=6.9 mg/L (48h)<br>ii) LOEC for larval growth =3.5 mg/L<br>i) Hazard quotient for larval growth = $1.1X10^{-4}$	Dobbins et al., (2009)
	BTP	Larvae (1 dpf)	5 concentrations (3.1-24.2 mg/L) (48h)	i) LC50=4.2 mg/L (48h)<br>ii) LOEC for larval growth = 1.0 mg/L<br>i) Hazard quotient for larval growth = $6.5X10^{-5}$	Dobbins et al., (2009)
	BNP	Larvae (1 dpf)	5 concentrations 2.1-16.0 mg/L) (48h)	i) LC50=3.3 mg/L (48h)<br>ii) LOEC for larval growth = 1.7 mg/L	Dobbins et al., (2009)

				i) Hazard quotient for larval growth = $2.3X10^{-4}$	
Japanese medaka	MTP	Larvae (10 dpf)	5 different concentrations; for 96 h	i) The 96 h LC50 is 63,000 µg/L (50,000-93,000)	Yamamoto et al., (2011)
	MTP	Adult male (3-month-old)	40, 200, 1000, 5000, and 25,000 µg/L for 14 days	iii) Plasma VTG level increased after exposure to 780 µg/L (experimental concentration was 1000 µg/L)<br>iv) NOEC for VTG was 160 µg/L (experimental concentration was 200 µg/L)<br>v) Upregulation of 13 genes including *vtg2, chgL, chgH, esr1* and downregulation of 10 genes occurred in adult male fish exposed to 40 µg/L	Yamamoto et al., (2011)

				(actual concentration 10 μg/L) of MTB.	
	ETP	Larvae (10 dph)	Five different concentrations; 96 h	i) 96 h LC50 is 14 mg/L	Yamamoto et al., (2011)
	PPP	Embryos (stages 7-8)	40, 400,1000, 4000 μg/L; exposure durations (stages 7-8 [3-4 hpf]-240 hpf) and evaluated at 76,124,196,244, 316 hpf for embryos. 13 dpf as eleutheroembryos, 28 and 43 dpf as larvae	Embryos<br>i) Survivability affected in a concentration-dependent manner (no effect in 1000μg/L but reduced in 4000 μg/L)<br>ii) Concentration-dependent significant dilation of the gall bladder seen during embryonic development<br>iii) No effect on p4501A activity while p4501A agonist (β-naphthoflavone) resulted synergistic response in	Gonzalez-Doncel et al., (2014)

					ethoxyresorufin-O-deethylase (EROD) activity.	
				Larva:	Survivability affected in a concentration-dependent manner (<1000 µg/L)	
	n-PPP	Larvae (10 dph)	Five different concentrations; 96 h	vi)	96 h LC50 is 4.6 mg/L	Yamamoto et al., (2011)
	i-PPP	Larvae (10 dph)	Five different concentrations; 96 h	vii)	96h LC50 is 4.5 mg/L	Yamamoto et al., (2011)
	PPP	Adult (male)	0.055,0.55, 5.5, and 55 mM/one week	viii)	Plasma VTG concentration increased	Inui et al., (2003)
				ix)	*vtg1, vtg2, chgL, chgH, esr1* and *esr2* mRNAs in liver were upregulated in a concentration-dependent manner. Androgen receptor mRNA (*ar*) remained unaltered.	

	i-BTP	Larvae (10 dph)	Six different concentrations; 96 h	i) 96h LC50 was 4.6 mg/L	Yamamoto et al., (2007)
	i-BTP	Adult (2/5 months old males)	4,20,100,500 µg/L/14 days	i) Concentration-dependent increase in plasma VTG level in male fish	Yamamoto et al., (2007)
	n-BTP	Larvae (10 dph)	Six different concentrations; 96 h	i) 96h LC_{50} (determined on 10-day larvae) is 2.9 mg/L	Yamamoto et al., (2007)
	n-BTP	Adult (2/5 months old males)	8,40,200, and 1000 µg/L/14 days	i) Concentration-dependent increase in plasma VTG level in male fish	Yamamoto et al., (2007)
	BNP	Larvae (10 dph)	Six different concentrations; 96 h	i) 96h LC_{50} (is 0.73 mg/L	Yamamoto et al., (2007)
	BNP	Adult (2/5 months old males)	4,20,100, and 500 µg/L; 14 days	ii) Concentration-dependent increase in plasma VTG level in male fish<br>iii) The number of upregulated genes in male fish by BNP are	Yamamoto et al., (2007)

				concentration-dependent<br>iv) The number of downregulated genes in male fish by BNP are nonlinear	
	BNP	Adult (2/5 months old males)	4µg/L; 14 days	Gene expression of p53, P4503A40, and chgL increased	Yamamoto et al., (2007)
Nile Tilapia	MTP	Adult male (3-5 months old)	17.1-136.5 mg/L; 48 h	i) The 48 h LC50=67.11 mg/L	Silva et al., (2018)
	MTP	Adult male (3-5 months old)	4 mg/L exposed for 6 and 12 days.	i) Superoxide dismutase (SOD) activity increased after 12 days of exposure<br>ii) Catalase (CAT) activity increased in liver after 6 days exposure, not after 12 days.<br>iii) Glutathione peroxidase (GPx) activity increased in	Silva et al., (2018)

				gills after 6 days exposure<br>iv) Glutathione reductase (GR) activity in liver increased after 12 days exposure<br>v) Glutathione (GSH) level remained unchanged in gills; however, in liver, decreased in 6 days and increased after 12 days exposure.<br>vi) Malondialdehyde (MDA) content in liver and gills remained unaltered.	
	ETP	Adult male (3-5 months old)	5.5-43.9mg/L; 48 h	i) The 48 h LC50 is 24.08 mg/L (18.70-31.02 mg/L)	Silva et al., (2018)
	ETP	Adult male (3-5 months old)	4 mg/L; 6 and 12 days	i) SOD activity increased in the gills after 12 days exposure	Silva et al., (2018)

				ii) The CAT activity in gills did not alter either in 6 or 12 days of exposure; in liver, CAT activity increased in 6 days, however, remained unaltered after 12 days of exposure<br>iii) The GPx activity in gills increased in both 6- and 12-days exposure<br>iv) The GR activity in liver increased after 12 days of exposure<br>v) The GSH content of the gills did not change; in liver, the GSH content increased in both 6 and 12 days of exposure.<br>vi) MDA level did not alter in gills;	

				however, in liver, a decrease was observed after 12 days exposure.	
	PPP	Adult male (3-5 months old)	3.1-24.8 mg/L; 48 h	i) The 48 h LC50 is 17.36 mg/L (14.63-20.61)	Silva et al., (2018)
	PPP	Adult male (3-5 months old)	4 mg/L; exposed for 6 and 12 days	i) SOD activity in gills increased after 12 days exposure; liver SOD remained unaltered in fish in both 6- and 12-days exposure periods<br>ii) CAT activities in gills and liver remained unaltered in both 6 and 12 days of exposure<br>iii) GPx activity increased only in gills after 6 days exposure; gills after 12 days of exposure and liver in both 6	Silva et al., (2018)

				and 12 days exposure, the GPx activity remained unaltered.<br>iv) GR activity in gills and liver remained unaltered in fish exposed to PPP in both exposure days (6 and 12 days).<br>v) GSH content in gills remained unaltered in fish exposed to PPP in both treatment days (6 and 12 days); in liver, the GSH content was initially decreased in 6 days exposure groups, while increased after 12 days of exposure.<br>vi) MDA level remained unaltered in both liver and gills after 6-	

				and 12-days exposures.	
	BTP	Adult male (3-5 months old)	2.7-21.5 mg/L.<br>48 h	i) LC 50 after 48 h of exposure was 7.80 mg/L (5.38-11.83 mg/L)	Silva et al., (2018)
	BTP	Adult male (3-5 months old)	4 mg/L; 6- and 12-days exposure	i) SOD activity increased in gills only after 12 days exposure; in liver, enhancement was observed only in 6 days not in 12 days.<br>ii) CAT activity in both gills and liver remained unresponsive to BTP both at 6- and 12-days exposures<br>iii) GPx activity in gills remained unaltered after 6- and 12-days exposure; in liver, GPx activity	Silva et al., (2018)

				increased only after 12 days exposure<br>iv) GR activity remained unaltered in both gills and liver in 6 and 12 days<br>v) GSH content in gills increased in fish only after 6 days of exposure; however, in liver, GSH content was decreased initially in 6 days and then increased in fish exposed to BTP for 12 days.<br>vi) MDA levels did not alter in gills in fish exposed for 6 or 12 days; in liver, a decrease in MDA content was observed in fish exposed for 12 days.	

	BTP	Adults	5,50,500, and 5000 ng/L: 56 days	i) Increased darker skin pigmentation<br>vii) Gene expression analysis showed that expression of *α-MSH (*upregulation*), asip2 (*downregulation*),* significantly changed<br>viii) Reduced dopamine and γ-aminobutyric acid content in brain<br>ix) Significant upregulation of the expression of *arr3a* and *arr3b* and downregulation of *opsin* in a concentration-dependent manner	Liu et al., (2023)
	BNP	Adult male (3-5 months old)	2.1-16.9 mg/L; 48 h	i) 48 h LC50 is 7.98 mg/L (5.38-11.83 mg/L)	Silva et al., (2018)
	BNP	Adult male (3-5 months old)	4 mg/L; exposed for 6 and 12 days.	i) SOD activity in gills increased after 6 days exposure and	Silva et al., (2018)

				continued to increase after 12 days;; in liver, SOD activity remained unaltered in fish exposed to BNP in both 6 and 12 days<br>ii) CAT and GR activities remained unalter in gills and livers of both 6- and 12-days exposed fish<br>iii) GSH content in gills remained unaltered, while increased in liver after 12 days exposure<br>iv) MDA content remained unaltered in both liver and gills in fish exposed to BNP in both 6 and 12 days.	
Rainbow trout	ETP	Juvenile (80-120g)	100 and 300 mg/kg/ injected 0 and 6 days/ final assays on 12 days.	x) Dose-dependent induction of serum VTG observed in 6-	Pedersen et al., (2000)

					and 12-days exposure (induced only in 300 mg/kg not in 100 mg/kg).	
	PPP	Juvenile rainbow trout (80-120 g)	100 and 300 mg/Kg/ injected on 0 and 6 days of experiment/ assessed on 12th day of experiment	i)	Dose-dependent increase in plasma VTG level both on 6 and 12 days of investigation.	Pedersen et al., (2000)
	PPP	Sexually immature rainbow trout	7-1830 µg/kg/every second day until 10 days orally	ii)<br><br>iii)	Increase in plasma VTG levels<br>ED50 values for increase in VTG synthesis were 35,31, and 22 mg/kg/2day at 3, 6 and 11 day of treatment, respectively	Bjerregaard et al., (2003)
	PPP	Sexually immature rainbow trout	50 and 225 µg/kg/every second day until 12 days by immersion	i)	Increase in plasma VTG levels is concentration-dependent (increase in 225 µg/L; not in 50 µg/L)	Bjerregaard et al., (2003)

				ii) Accumulation of PPP in liver and muscle of fish exposed to 225 µg/L for 12 days, was investigated. The accumulated PPP was 6700 µg/kg liver and 870 µg/kg muscle.<br>v) Half-lives of PPP in liver was 8.6 h and in muscle 1.5 h.	
	BTP	Sexually immature rainbow trout	/4 -74 mg/kg/every second day until 10 days orally	i) Dose and time-dependent increase in plasma VTG levels<br>ii) ED50 for VTG response is 10.5 mg/kg/2d<br>vi)	Alslev et al., (2005)
	BTP	Sexually immature rainbow trout	35 and 201µg/L for 12 days by immersion	i) Increase in plasma VTG levels is concentration-dependent (increase in 201 µg/L; not in 35 µg/L)	Alslev et al., (2005)

				ii) A positive correlation exits in the concentration of BTP and the VTG in the plasma<br>vii) Uptake of BTP from the environment to the body of the fish was 13 mg/kg/day for fish exposed to 35 µg/L and 78 mg/kg/day in fish exposed to 201 µg/L	
	BTP		5,50,500, 5000 ng/L/56 days	i) Induced darker skin pigmentation in a concentration-dependent manner<br>ii) The gene expression related to pigmentation (*α-MSH* and *Asip2*) changed<br>iii) Reduced dopamine and γ-aminobutyric acid content in the	

				brain (related to the synthesis of *α-MSH)*<br>iv) Expression of *Arr3a* and *Arr3b* was upregulated, however, *Opsin* expression downregulated in a concentration-dependent manner<br>viii) Inhibited phototransduction from the retina to the brain	
Zebrafish	MTP	Embryos (4-6 hpf)	100,200,400,800,1000 μM; 96 h	i) 96 h LC50 value is 65 mg/L<br>ii) Concentration-dependent decrease in heart rates<br>iii) Concentration-dependent decrease in hatching rates<br>iv) Morphological abnormalities (pericardial edema,	Dambal et al., (2017)

				bent spine, blood cell accumulation) increased at the concentration-dependent manner<br>v) *vtg1* expression enhanced	
	MTP	embryos	50 mg/L; 5 days	i) LC50= 50 mg/L<br>ii) Tail defects, pericardial edema, and pigmentation defects observed (increased with MTP exposure)<br>iii) Inhibition of locomotor activity<br>iv) Reduced GST and NO levels<br>v) Lipid peroxidation (LPO) enhanced<br>vi) MDA content enhanced<br>vii) Increased expression of *ccdn1* (proto-oncogene) and *myca*	Ates et al., (2018)

				(cellular proliferation) mRNAs	
	MTP	Embryos (2 hpf)	1,10,25,50,100 and 200µM; 120 hpf	i) No significant mortality was observed<br>ii) Hatchig delay was not observed<br>iii) Concentration-dependent malformation in embryos/larvae at 96 hpf (pericardial edema and spinal defects)<br>iv) Downregulated stress-related microsomal glutathione S transferase (*mgs*t), and glutathione S transferase (*gst*) genes by MTP	Bereketoglu and Pradhan (2019)

				v) *cat* and *sod3* did not show any significant change<br>vi) *hsp70* and metallothionein (*mt1*) did not show any significant changes<br>vii) no alteration in the expression of *bax* and *bcl2*<br>viii) a decreased expression of growth arrest and DNA damage inducible alpha (*gadd45a*)<br>ix) expression of *tnfα* and *il8* was affected by MTP exposure<br>x) downregulation of *ldlr* occurred.<br>xi) Repressed the expression of *ar*	

	MTP	Embryos	0.1, 1, 10, 100 ppb; until 6 dpf	i) Concentration-dependent decline in hatching rates<br>ii) Concentration-dependent decrease in heart rates (48 hpf)<br>iii) Anxiety-like behavior significantly higher<br>iv) No significant difference in scototaxis (light-dark preferences)<br>v) Concentration-dependent decrease in AChE activity<br>vi) Whole body cortisol level increased in a concentration-dependent manner	Raja et al., (2019)
	MTP	Fertilized eggs (1-3 hpf)	1, 10, 30, 60, 80 mg/L; 96 hpf	i) 96 h LC50 is 72.67 mg/L (BMDL-BMDU=40.8-57.4 mg/L	Merola et al., (2020b)

				ii) Embryos exposed to 60-80 mg/L died by 24 h.<br>iii) TI= 16-26.5 mg/L<br>iv) Significant reduction in hatching rates (72 hpf)<br>v) Notochord curvature was increased significantly in a concentration-dependent manner<br>vi) Reduced heartbeats, blood stasis, and reduction in blood circulation (48 h) in a concentration-dependent manner<br>vii) Concentration-dependent development of pericardial edema and yolk sac edema (48hpf)	

	MTP	Embryos (1 hpf)	100, 1000, 10000 µg/L; 4-6 days	i) No significant effect on thigmotaxis<br>ii) No significant effect on startle response<br>iii) Did not affect photic entrainment of locomotor activity	Merola et al., (2021)
	MTP	Embryos (2 hpf)	1,5, 20, 100, 200 µM;120h	i) Significant reduction in body length and heartrates<br>ix) Did not exert any significant influence on the survival rate of zebrafish larvae (120 hpf)<br>x) Concentration-dependent decrease in hatching rate (200 µM)<br>xi) Concentration-dependent induction in malformations in cardiac edema and spinal curvature (100 and 200 µM)	Liang et al., (2022)

				xii) Decrease in both T3 and T4 levels<br>xiii) Gene expression analysis indicated the transcriptional level of transthyretin (*ttr*) was significantly decreased in larvae exposed to 200 µM	
	MTP	Embryos (2 hpf)	20, 100, 200 µM/120h	ii) The vtg concentration in the larvae increased in a concentration-dependent manner (120 hpf)<br>iii) The level of T was downregulated in all concentrations of MP (20-200 µM) used.	Liang et al., (2023a)
	MTP	Embryos (2 hpf)	20, 100, 200 µM/120 hpf	i) Inhibited the total movement and mean velocity in a concentration-dependent manner (significantly differ	Liang et al., (2023b)

				from controls only in the larvae exposed to 200 μM)<br>ii) Significant increase in AChE enzyme activity in a concentration-dependent manner<br>iii) Concentration-dependent increase in the cortisol levels and decrease in the ACTH levels in all treatment groups.<br>iv) The downregulation of *gr, mr* and *crhr2* genes and upregulation in *pmoc* genes in the HPI axis (hypothalamus-pituitary-interrenal gland)	
	MTP	Embryos	0.5, 50, 500, 5000,100,000 μg/L;	i) Caused 33% mortality at the	Medkova et al., (2023)

			96 hpf	concentrations 100,000 µg/L concentration<br>ii) Significantly delayed hatching in a concentration-dependent manner<br>iii) Downregulation of *hsp70l* and *hsp90*	
	MTP	Embryos (2-3 hpf)	160 µM at 56 hpf	i) Heart rate increased<br>ii) Cardiac output remained unaltered<br>iii) Stroke volume remained unaltered	Shi et al. (2023)
	MTP	Embryos (4hpf)	5,10,20, 40, 80, 150, 300 µM; 120 hpf	i) LC50 (120 hpf) was 468.14 µM (309.51-626.78 µM)<br>ii) EC50 (120 hpf) =255.61 µm (220.7-290.4 µM)<br>iii) Teratogenic index (TEI) 1.8 (120 hpf)<br>iv) Mortality point of departure (M-POD)	Tran et al., (2023)

				is 154.1 µM (4-120hpf)<br>v) Malformation-based point of departure (Mal-POD) was 196.6 µM<br>vi) No significant difference in locomotor activity during both light and dark phases at all tested concentrations<br>vii) Total differentially expressed genes (DEG) were 166 of which 119 downregulated and 47 upregulated genes were observed.	
	MTP	Larvae and adults	60, 102, 173,294, 500mg/l for larvae, exposed for 168 h. Adults 30,48,67.5, 101 and 105 mg/L exposed for 96h	i) The LC50 was 105.09 mg/L for adults and 211.12 mg/L for larvae.<br>ii) In adults (50 mg/L), ethoxyresorufin O-	De Carvalho Penha et al., (2021)

				deethylase activity (EROD) in gills significantly decreased.<br>iii) LPO did not differ in liver, augmented in gills<br>iv) The frequency of micronuclei in erythrocytes significantly increased (adults, exposed to 50 mg/L)<br>v) Gut microbiota did not vary	
	MTP	Adult (male)	0.001, 0.01,1.0, 10.0 mg/L: 21 days	i) The length and weight of the fish remained unaltered<br>ii) Concentration-dependent decrease in GSI<br>iii) General atrophy, multinucleated gonocytes, impaired	Hassanzadeh, (2017)

				germ cells, spermatogonial proliferation, Leydig cell proliferation, interstitial fibrosis and apoptosis of Sertoli cells.	
	MTP	Adult (males and females)	1,10, 110 ppb ;30 days	i) LC 50 was 1.102 ppb for MTP in adult zebrafish (1.102 µg/L)<br>ii) Increase in average distance travelled by both male and female fish on 15-day exposure; while significant decreased in average distance travelled by both male and female fish exposed for 30 days<br>iii) Swimming speed in females increased in both 15 and 30 days in fish exposed to	Thakkar et al., (2022)

				MTP (all concentrations); in males, increase in swimming speed was observed in lower concentration groups and decrease in highest concentrations on 15 days exposure; in 30 days exposure swimming speed in male fish deceased in all exposure groups when compared with controls<br>iv) More anxiety-like behavior observed in females than male<br>ii) Concentration-dependent reduction in AChE activity<br>iii) Concentration-dependent increase in serotonin level in	

				female fish while in male fish concentration dependent reduction in serotonin level<br>iv) Expression of *hif-1α*, *tnn2, pax-6b*, *ntrk2a*, were altered (mostly downregulated in higher concentrations).	
	MTP	Adults	1,3, and 10 µg/L/ 28 days	i) Disrupt the composition and diversity of gut microbial community<br>ii) Increased the body length and weight of the female fish<br>iii) In intestine of males, increase in goblet cell density, tight junction protein 2 (TJP2), and serotonin concentrations	Hu et al., (2022b)

				iv) In females lower density in intestinal goblet cells, inhibited expression of TJP2 and reduced the concentration of serotonin but upregulated the expression of proinflamatory cytokines.<br>i) Intestinal catalase activity enhanced.	
	MTP	Adults (4 months)	1,3,10 µg/L/28 days	i) Significant decrease in hepatosomatic index (HSI) in female fish exposed to 1 and 10 µg/L<br>ii) Induced hepatocellular vacuolization<br>iii) Significant inconsistent enhancement of alanine	Hu et al., (2022a)

				aminotransferase (ALT) activity (only in 1μg/L) in both males and females<br>iv) Liver ROS in females significantly higher in fish exposed to 10 μg/L, which was accompanied by inhibition of catalase (CAT) activity (3 μg/L)<br>v) GSH content in male liver decreased in a concentration-dependent manner, however, GPx activity in both male and females enhanced in a nonlinear fashion<br>vi) In male liver, upregulation of peroxisome	

				proliferator-activated receptor gene (*pparα*) occurred in fish exposed to 3 and 10 μg/L<br>vii) Farnesoid X receptor gene *nr1h4* was downregulated in male fish in a concentration-dependent manner<br>viii) Inconsistent upregulation of sterol regulatory element binding proteins (srebp1) by MTP (1 and 10 μg/L) in male liver decreased in a concentration-dependent manner<br>ix) The free fatty acid content in male liver<br>x) In female liver concentration-dependent	

				downregulation of PARP, FAX and SREBP genes observed.<br>xi) The intestinal tissue contains significantly higher concentrations of triglycerides (TG) but lower concentration of glycerol, and free fatty acids (FFA) in male fish exposed to MTP<br>xii) In blood the concentration of TG, total cholesterol (TCHO) HDL-cholesterol (HDL-C), LDL-Cholesterol (LDL-C) decreased by MTP exposure<br>xiii) In females, concentrations of TG in intestine, blood,	

				and liver tissues, were significantly decreased after MTP exposure; moreover, concentrations of TCHO and HDL-C were altered inconstantly<br>xiv) Higher level of cortisol was observed in male liver	
	MTP	Adults	1,3,10 µg/L/28 days	Significant decrease in cranio-somatic index (CSI) in female fish exposed to 3 µg/L MTP<br>ii) A concentration-dependent increase in ROS in male brain and a concurrent decrease in CAT activity<br>iii) Males exposed to 1 µg/L reduced GSH	Hu et al., (2023a)

				content in brain and enhanced GPx activity<br>iv) Lower extents of MDA and LPO were accumulated in both male and female brains<br>v) Neural proteins in male brains exposed to 10 µg/L (*isv2ca, syncripl, rims3, grik2, thrap3b*) and in females, (*neflb, gap43, olfm1b, syap1, chm1, nlgn2a, rs1a, thrap3b*) were altered<br>vi) Enhanced the glutamate content in male brain but decreased in female brain<br>vii) AChE activity remained unaltered	

				viii) Downregulation of α1 tubulin transcripts in both male and female brains of zebrafish by MTP exposure<br>ix) Decreased blood cortisol concentrations in both males and females<br>x) Downregulation of corticotropin releasing hormone (*crh*) and corticotropin-releasing hormone binding protein (*crhbp*) genes in the brain of both male and female zebrafish<br>xi) disruption of immune system genes including *c4, igl4v8, cd79a, igbp1, cd59,*	

				c5 in brain of female fish<br>xii) significant increase in blood-brain-barrier protein (BBB) in female brain by MTP (10 µg/L) exposure<br>xiii) significant increase in LPS endotoxin concentration in male brain by MTP exposure (10 µg/L), however decreased in females (3-10 µg/L)<br>xiv) transcription of *il1β* and *il6* were upregulated in male brain	
	MTP	Adults	1.3, 10 µg/L/ 28 days	i) increased GSI in a concentration-dependent manner<br>ii) spermatogenesis and oogenesis were blocked	Hu et al., (2023b)

				iii) parental exposure induced developmental deficits in larvae by increasing mortality, stimulating precocious hatching, and elevating heart rates.<br>iv) Blood concentrations of E2, T, and 11-keto testosterone were consistently lowered<br>v) Transcriptional results showed that disruption in the hypothalamus-pituitary-gonadal axis<br>vi) Hepatic VTG downregulated	
	ETP	Embryos	1,5,10,20, and 30 mg/L/96 h	vii) LC50 values at 96h was 20.86 mg/L; benchmark dose	Merola et al., (2020a)

				(BMD) was 10.8-17.4 mg/L<br>viii) 85% mortality was observed in embryos exposed to 30 mg/L at 96 hpf<br>ix) Significant reduction in hatching rate at 72 hpf<br>x) Concentration and time-dependent malformation induced reduction in blood stasis, pericardial edema, misshaped yolk sac and deformed notochord<br>xi) Defects in pectoral fin development (concentration and time-dependent)<br>xii) Concentration and time-dependent induction in	

				behavioral abnormalities (treambling of head, pectoral fins, spinal cord, and circling behavior	
	ETP	Embryos (6 hpf)	0.1, 0.5, 1, 2, 3, 4, 5, 6,10,15,18,20, 25,30,42,50,80, 100 mg/L/embryos 48-96hpf;	i) Calculated LC50 is 28.70 mg/L<br>xv) Teratogenic concentration (TC50) is 20.63 mg/L<br>xvi) Induced abnormal cardiac function (heart rates) and morphology (pericardial effusion)<br>xvii) Disrupts retinoic acid signaling pathway related to original cardiac catheter development<br>xviii) Inhibition of gene expression related to myocardial contraction	Fan et al., (2022)

				xix) Orientation development disturbance of heart tube (oriented toward the right or middle side)<br>xx) Gene expression analysis indicated that ETP (20 mg/L) upregulated 1302 genes and downregulated 2482 genes.<br>xxi) The key genes related to apoptosis (*casp3, casp9, afap111b ,gadd 45ga, cflts*) were upregulated after ETP (20 mg/L) exposure<br>xxii) Among the downregulated genes, the expression of *ttc36*, a gene	

				associated with heart tube orientation, was also downregulated	
	ETP	Embryos	50, 500, 5000 µg/L/ exposed until 4 dpf/ behavioral analysis was on 4,5, and 6 dpf	i) Increased anxiety-like behavior in a concentration-dependent manner<br>i) Did not influence visual startle response and the photic synchronization of circadian rhythms	Merola et al., (2021)
	ETP	Embryos	1, 5, 10, 20, 30mg/L; 96 hpf	ii) LC50 is 20.86 mg/L<br>iii) Reduced heartbeat, reduction in blood circulation, blood stasis, pericardial edema, deformed notochord, misshaped yolk sac<br>i) Induced behavioral changes (trembling of head, pectoral fins and spinal cords)	Merola et al., (2020a)

	ETP	Embryos (2hpf)	1,5, 20,50, 100 µM, 120 hpf	i) Body length and heart rate reduced in a concentration-dependent manner<br>ii) Significant reduction in the survival rate of the embryos exposed to 200 µM.<br>iii) Hatching was reduced in embryos exposed to 50-100 µM ETP<br>iv) Malformation rate (cardiovascular edema and spinal curvature) was significantly increased in a concentration-dependent manner<br>v) Significant reduction in T3 and T4 contents of the larvae exposed to 50-100 µM ETP	Liang et al., (2022)

				vi) Induced expression of *tshβ* in a larvae exposed to 100 µM ETP<br>vii) Induced significant increase in trα (20-100 µM) ETP<br>viii) Decreased in the expression of *pax8* (20-100 µM) and *ttr* (50-100 µM)	
	ETP	Embryos (2 hpf)	20,50, 100 µM/120 hpf	i) Vtg content enhanced in 20 and 50 µM concentrations<br>ii) The level of T was down regulated (2-100 µM)	Liang et al (2023a)
	ETP	Embryos (2 hpf)	20,50, 100 µm/120 hpf	i) Significant inhibition in the total movement distance covered by larvae in lower concentrations (20 and 50 µM)<br>ii) Significant increase in AChE activity in	Liang et al., (2023b)

				larvae (120 hpf) exposed to 50 µM ETP.<br>iii) Significant concentration-dependent decrease in ACTH level<br>iv) Significant concentration-dependent enhancement in the cortisol level<br>v) Significant inconsistent concentration-dependent downregulation in the mRNA levels of *mr, crh2, crhbp* (except *gr*) were observed.	
	ETP	Embryos (4hpf)	5,10,20, 40, 80, 150, 300 µM; 120 hpf	i) LC50= 196 µM (138.27-257.384 µM) (120 hpf)<br>ii) EC50=146.8 µM	Tran et al., (2023)

				(123.4-170.27 µM)<br>iii) TEI=1.3 (ratio of LC50/EC50)<br>iv) Hyperactivity was observed in ETP (~72.6 µM) in the acclimation and reduction darkness phases<br>v) 414 genes were differentially expressed of which 352 genes were upregulated and 67 genes were downregulated.	
	PPP	Embryos (2 hpf)	1,2, 5, 10 and 20 µM. 120hpf	i) Concentration-dependent decrease in body length and heart rates<br>ii) Significant decrease in survival rate and hatching of the embryos	Liang et al., (2022)

				iii) Morphological alteration resulted malformation in embryos/larvae (yolk sac edema/ tail curvature) increased (10 and 20 μM)<br>iv) Significant reduction in the T3 and T4 levels in embryos (5-20 μM)<br>v) Upregulation of *tshβ* (10-20 μM)<br>vi) Upregulation of *tg*, *nis*, and *dio1* (5-10 μM)<br>vii) Upregulation of *nkx2.1*, *ttr*, and *ugt1ab* only in larvae exposed to 5μM group<br>viii) Upregulation of *trα* was occurred in 10 μM group	

				ix) Downregulation of *pax8* was occurred in larvae exposed to 5 μM group	
	PPP	Embryos (2-3 hpf)	10 and 1000 μg/L; 24h	i) No significant changes in total and individual oxysterols compared to control groups, however, 27-OH showed a reduction at 24h exposure	Merola et al., (2022)
	PPP	Embryos (2 hpf)	2,5, and 10 μM/120hpf	ii) Vtg content enhanced in 2 μM concentration<br>iii) The level of T was down regulated (2-10μM)	Liang et al (2023a)
	PPP	Embryos (2 hpf)	2,5, and 10 μM/120hpf	i) Significant concentration-dependent decrease in the total distance covered by the 120 hpf larvae by swimming and the	Liang et al (2023b)

				mean velocity (significant reduction was observed in larvae exposed to 10 µM of PPP)<br>ii) AChE activity remained unaltered.<br>iii) Concentration-dependent decrease in the level of ACTH and enhancement in the level of cortisol<br>iv) Inconsistent concentration-dependent down regulation of *gr, mr, crhr2, crhbp* in the HPI axis. No significant effect was observed in the mRNA expression level of *pmoc* gene.	
	PPP	Embryos	10, 100, 1000, 1500, 6000,8500, 10,000 µg/L/8, 32, and 80hpf	i) NOEC= 1000 µg/L (Exposure to 10 mg/L is lethal)	Torres et al., (2016)

				ii) Reduced heartrate in time and concentration-dependent manner<br>iii) Concentration and time-dependent abnormalities observed in eyes, head, pericardial edema, yolk sac, and tail development	
	PPP	Embryos (2 hpf)	2hpf/1,10,25, 50,100, and 200 µM/2hpf-120hpf	i) Concentration-dependent mortality (100% mortality in concentration above 10µM)<br>ii) Hatching delay observed (10 µM)<br>iii) Malformed embryos observed (pericardial edema and spinal defects at 10 µM concentration)	Bereketoglu and Pradhan (2019)

				iv) Gene expression analysis (1 and 10 µM) showed decrease expression of nuclear factor, *erythroid 2-like 2a (nrf2), kelch- ECH-associated protein 1 (keap1),* and sod1<br>v) Downregulation of microsomal glutathione S-transferase (*mgst*) and glutathione S-transferase (*gst*)<br>vi) *Catalase* and *sod3* did not alter<br>vii) Significant upregulation of *hsp70* and metallothionein 1 (*met1*); however, no alteration was observed in *met2* gene.	

				viii) Downregulation of caspase 3a (*casp 3a*), death associated protein 3 (dap3), BCL2apoptosis regulator 2 (*bcl2*), while increased BCL2 associated X regulator (*bax*)<br>ix) Downregulation of cyclin-dependent kinase inhibitor 1A (p21) and induction of mitogen activated protein kinase 14a (*p38*) was observed (10 μM)<br>x) Decreased expression of growth arrest and DNA- damage inducible alpha (*gadd45a*) was observed<br>xi) Downregulation of RAD51 recombinase	

				(*rad51*) and APEX nuclease (multifunctional DNA repair enzyme)1 (*apex 1*) and upregulation in xeroderma pigmentosum, complementation group C (*xpc*) by PPP was observed<br>xii) The expression of *tnfα* and *il8* was altered significantly<br>xiii) Downregulation of apolipoprotein genes involved in fatty acid transport (*apoab, apoeb, apoa4*) and fatty acid synthesis (*fasn*)<br>xiv) Downregulation of low-density lipoprotein receptor (*ldlr*) by 10 μM PP	

				xv) Upregulation of *lipase, hepatic (lipc*) was occurred (both 1 and 10 μM)<br>xvi) Repressed the expression of *ar* and upregulated the expression of *esr2a*<br>xvii) Increased expression of thyroid hormone receptor αa (*thraa*) and thyroid hormone receptor β (*thrb*)	
	PPP	Embryos	1,2,4,6,8 mg/L. 96hpf	i) LC50 is 3.98 mg/L<br>ii) Enlarged and misshaped yolk sac<br>iii) Reduction in hatching rates (evaluated at 72 hpf)<br>iv) All embryos died at 96 hpf when exposed to 8 mg/L<br>v) malformation of the larvae (reduced heart beats, blood stasis,	Perugini et al., (2020)

				reduction in blood circulation, pericardial edema, enlarged yolk sac, deformed tail) induced in a concentration-dependent manner<br>vi) Hyperexcitability<br>vii) Reduction in head size and swim bladder<br>viii) Decrease in neutral lipid metabolism in yolk<br>ix) Alteration in phospholipid metabolism (reduction in PLA2 enzyme activity) both in body and yolk sac<br>x) Deformed neurocranium	

	PPP	Embryos (2 hpf)	0.1,1, and 10 µg/L/exposed for 2-96 hpf.	i) Hatching rate declined only at 48 hpf, not in 24hpf, 72 hpf and 96 hpf<br>ii) Survivability of the embryos seem to be time and concentration-dependent<br>iii) Concentration-dependent induction in the malformation (bent spine, yolk sac edema, pericardial edema, red blood cell accumulation) of the embryos occurred.<br>iv) Significant concertation-dependent decrease in heart rate (observed on 96 hpf)<br>v) Trigger anxiety-like behavior in larvae (6dpf)	Lite et al., (2022)

				(concentration-dependent reduction in the time spent in the light zone).<br>vi) The exploratory behavior (the number of transitions between light and dark compartments) significantly reduced in a concentration-dependent manner)<br>vii) Significant increase in intracellular ROS and LP<br>viii) SOD, CAT, GPx, GST, GSH activity/concentration in the head region of the larvae decreased/suppressed<br>xi) Suppression of AChE activity and increase in nitric oxide production	

				(concentration-dependent)	
	PPP	Embryos	0.1;10;100;1000; 100,000 µg/L/96 hpf	i) 100 % mortality was observed in embryos exposed to 100,000 µg/L<br>ii) Did not induce any developmental abnormalities as observed at 72 hpf<br>iii) Delayed hatching in a concentration-dependent manner (only in 1000 µg/L)<br>iv) Upregulation of *hsp70l* mRNA exposed to 0.1 µg/L<br>v) Expression of gstp2 was upregulated after exposure to 0.1 µg/L<br>vi) Upregulation of both *cyp17a1* and *cyp19a1a* embryos exposed to 0.1 and 100 µg/L	Medkova et al., (2023)

	PPP	Embryos (4hpf)	5,10, 20, 0, 80, 150, 300 µM; 120 hpf	i) Calculated 120h LC50= 61.8 µM (39.5-84.2 µM)<br>ii) Calculated 120h EC50=40.8 µm (35.5-46.1 µM)<br>iii) TI= 1.5<br>iv) No significant differences in locomotor activities during both light and light phases at all tested concentrations.<br>v) 315 genes were differentially regulated of which 181 gene s were upregulated and 134 genes were downregulated	Tran et al., (2023)
	PPP	Embryos and larvae	10 and 1000 µg/L; 3hpf-4 dpf	i) Thigmotaxis was decreased in larvae exposed to 1000 µg/L during embryogenesis.	Merola et al., (2024)

				ii) Thigmotaxis was increased in juveniles exposed to 10 µg/L embryologically<br>iii) Expression of *shank3a* and *gad1b* was repressed by PPP (10-1000 µg/L)<br>iv) Proteomics analysis indicated alterations related to brain development and lipid metabolism.	
	PPP	Larvae (20 dph)	0.1, 0.4, 0.9 mg/L; 20 days	i) Decline in vitellogenin production (whole body)	Mikula et al., (2006a, 2006b)
	PPP	Larvae (20 dph)	500, 1000, and 2000 mg/kg via food; 20 days and 45 days	i) Length and weight did not alter<br>ii) No significant difference in whole body vtg content<br>iii) Sex ratio female biased (significant	Mikula et al., (2009)

				only in 500 mg/kg dose)	
	BTP	Embryos	0.1, 1, and 10 μg/L/ 2hpf- 96 hpf	iv) Hatching rate, survivability and malformation varied.<br>xii) Trigger anxiety-like behavior in larvae<br>xiii) Significant increase in intracellular ROS and LP<br>xiv) SOD, CAT, GPx, GST activity in the head region of the larvae suppressed<br>xv) Suppression of AChE activity and increase in nitric oxide production	Lite et al., (2022)
	BTP	Embryos (2 hpf)	0.5, 1, 2, 5 and 10 μM/120 hpf	i) Concentration-dependent decrease in body length and heartrate (5-10 μM)<br>ii) Nonlethal concentration of BTP	Liang et al., (2022)

				in zebrafish was 10 μM<br>iii) Concentration-dependent decrease in hatching (72 hpf) and heart rates (120hpf)<br>iv) Concentration-dependent enhancement in the malformation rate (cardiac edema and bent tail) induced in larvae (120 hpf)<br>v) Significant decrease in T3 (2-10 μM) and T4 (5-10 μM) levels by BTU (120 hpf)<br>vi) Significant downregulation of *crh, trh, tshβ, nkx2.1, hhex, ttr, dio1, dio2*, and *ugt1ab* (2-10 μM)	

				vii) Significant upregulation was observed in *trα* mRNA in larvae (120 hpf) exposed to 10 µM BTU.	
	BTP	Embryos (2 hpf)	1, 2 and 5 µM/120 hpf	i) Vtg content enhanced in 1 and 2 µM concentration<br>ii) The level of T was upregulated in 1 µM and down regulated in 2µM concentrations<br>iii) The level of estrogen (E2) was elevated exposed to 1 and 2 µM concentrations	Liang et al (2023a)
	BTP	Embryos (2 hpf)	1, 2 and 5 µM.<br>120 hpf	i) The total movement distance covered by the zebrafish larvae (120 hpf) and the mean velocity significantly reduced	Liang et al., (2023b)

				by BTP (only in 5 μM exposure group)<br>ii) No obvious change in the AChE activity in the larvae (120 hpf) was exposed to any of the concentration of BTP used in this study.<br>iii) No effect on the ACTH concentration, however, the cortisol level in BTP-exposed larvae (5 μM) reduced significantly<br>iv) Inconsistent concentration-dependent down regulation of *gr, mr, crhr2, crhbp* in the HPI axis. No significant effect was observed in the mRNA expression level of *pmoc* gene.	

	BTP	Embryos	0.25,0.5,1,2.5, and 5 mg/L; 96h	i) LC50 is 2.34 mg/L: benchmark dose (BMD) 0.91-1.92 mg/L (BMD confidence interval)<br>ii) 100 % embryos died after 48 hpf exposed to 5 mg/L BTP<br>iii) Hatching rate altered in a nonlinear fashion.<br>iv) Concentration-dependent reduction on heartbeats, blood circulation, blood stasis, pericardial edema, deformed notochord, and misshaped yolk was observed in embryos during 72-96 hpf of development<br>v) Defects in pectoral fin development	Merola et al., (2020a)

				vi) Induced behavioral changes (trembling of head, pectoral fins and spinal cords)	
	BTP	Embryos	5, 50, 500 µg/L/ exposed until 4 dpf/behavioral analysis was on 4,5, and 6 dpf	i) Increased anxiety-like behavior in a concentration-dependent manner<br>ii) Did not influence visual startle response and the photic synchronization of circadian rhythms	Merola et al., (2021)
	BTP	Embryos	0.1; 10; 100; 1000; 100,000 µg/L; 96 h	i) Induced 100% mortality in embryos exposed to 100,000 µg/L<br>ii) Cardiac edema and blood clots were observed in embryos in a concentration-dependent manner (1000 and 100,000 µg/L)	Medkova et al., (2023)

				iii) Induced hatching delay in a concentration-dependent manner (72 hpf)<br>iv) Upregulation of hsp70l in embryos exposed to 0.1 and 100 µg/L<br>v) Upregulation of *cyp19a1a* was observed in embryos exposed only to 0.1 µg/L	
	BTP	embryos	0.1,0.25.0.5, 0.75, 1,2,4,8, and 16 mg/L; 120 hpf	i) LC50 values decreases over time; (24h LC50=10.77 mg/L; 48 h LC50=4.208 mg/L, 72 h LC50=1.953 mg/L; 96 h LC50=1.359 mg/L; 120 h LC50=0.966 mg/L)<br>ii) Induced pericardial edema and ocular edema (0.5, 0.75, 1 mg/L) at 120 hpf.	Li et al., (2023)

				iii) The length, eyes, otoliths were significantly smaller<br>iv) Hatching rate reduced significantly<br>v) Maxillofacial cartilage deformed severely deformed<br>vi) Length of ceratohyal bone significantly reduced.<br>vii) The activities of catalase (CAT), and superoxide dismutase (SOD) reduced significantly, however, the activity of malondialdehyde (MDA) significantly elevated<br>viii) The activity of alkaline phosphatase is reduced significantly (marker	

				enzyme of osteoblast activity)<br>ix) Downregulation of the expression of *sox9a, sox9b* and *col2a1a* genes (chondrocyte marker genes)	
	BTP	Embryos (6hph)	0.6, 1.2, 1.8 mg/L; until 72 hpf	i) LC50 is 2.74 mg/L (72hpf)<br>ii) Embryos exhibited pathological effects including, spinal curvature, reduced eye size, and yolk sac edema, but no mortality<br>iii) Significant decrease in heart rates<br>iv) Concentrative-dependent pericardial edema and linearization was observed and showed	Zhu et al., (2023)

				cardiac impairment by BTP.<br>v) Impairs the cardiac contraction ability and cardiac output in a concentration-dependent manner<br>vi) Reduced blood cell counts in heart in a concentration-dependent manner and induced systolic heart failure<br>vii) Decrease in the expression of *klf2a* (the endocardial flow response gene)<br>viii) The heart failure genes, natriuretic peptides A and B (*nppa* and *nppb*) were significantly upregulated<br>ix) Induced apoptosis in heart	

				x) Damaged endocardial and atrioventricular valves by activating the endogenous apoptotic pathways.<br>xi) Impaired calcium homeostasis<br>xii) Depleted cardiac-resident macrophages<br>xiii) Cardiac immune system became non-responsive<br>xiv) Induced oxidative stress.	
	BTP	Embryos (*Tg(ins:GFP)*] (3hpf; mid-blastula transition)	250, 500, 1000, 3000 nM;<br>3h-7 dpf	i) LOEC for islet variant morphology was 250 nM and NOEC was 125 nM<br>ii) Islet area increased significantly<br>iii) Beta (β) cell area of the islet appear more dispersed, fragmented beta	Brown et al., (2018)

				cells,and ectopic beta cells emerge away from the primary islet.<br>iv) Islet fragmentation decreased over time<br>v) Deformity index (pericardial edema, yolk sac utilization, intestinal effusion, craniofacial malformations and spinal malformations) increased in a concentration-dependent manner<br>vi) No significant relationship was observed between BTP concentration and yolk sac area.<br>vii) Body length of the embryos did not	

				affect with the tested BTU concentrations<br>viii) Swim bladder inflation was significantly decreased by BTP in a concentration-dependent manner.<br>ix) GSH increased in a concentration-dependent manner<br>x) No alteration in the expression of *preproinsulin a (insa) glugagon a (gcga) ghrelin (ghrl),* and *somatostatin 2 (sst2)*. However, pancreatic duodenal homeobox 1 (*pdx 1*) was down regulated after BTP exposure.<br>xi) The expression of glutathione-S-transferases (*gstp* and	

				gsta1) modestly altered (*gstp* upregulated slightly, *gsta1* downregulated)<br>xii) No effect on glutamate-cysteine ligase (*gclm*), however *gclc* were modestly downregulated by BTP<br>xiii) Downregulation of glutathione synthase (*gss*) was observed by BTP exposure in a concentration-dependent manner.	
	BTP	Adult (male)	0.01, 0.1, 1.0 mg/L; 28 days	i) BTP penetrated the Blood brain barrier and impaired neurobehavior in photosensitivity and memory in a	Kim et al., (2022)

				concentration-dependent manner.<br>ii) RNA-seq analysis identified significant effect of BTP on phototransduction, tight junctions, neuroactive ligand receptor activity<br>iii) Cortisol increased and allopregnenolone levels decreased after BTP exposure<br>iv) Induced alterations in neurotransmitters belong to histaminergic, cholinergic, dopaminergic, serotonergic, and GABAergic systems.	
